# Comparative analysis of NSP5/VP2-induced viroplasm-like structures in rotavirus species A to J

**DOI:** 10.1128/jvi.00990-25

**Published:** 2025-10-14

**Authors:** Ariana Cosic, Melissa Lee, Kurt Tobler, Claudio Aguilar, Cornel Fraefel, Catherine Eichwald

**Affiliations:** 1 Institute of Virology, University of Zurich 30842, Zurich, Switzerland; University of Michigan Medical School, Ann Arbor, Michigan, USA

**Keywords:** rotavirus, species, viroplasm, NSP5, VP2, NSP2

## Abstract

Rotavirus (RV) is classified into nine species, A–D and F–J, with RV species A (RVA) being the most extensively studied. While RVA infects infants and young animals, non-RVA species infect adult humans, various mammals, and birds. However, the lack of appropriate research tools has limited our understanding of non-RVA life cycles. RVA replication and assembly occur in cytosolic inclusions termed viroplasms. We recently identified viroplasm-like structures (VLS) composed of NSP5 and NSP2 in non-RVA. In this context, globular VLS induced by NSP2 formed in RVA, RVB, RVD, RVF, RVG, and RVI, but not in RVC, RVH, and RVJ. Additionally, in RVA, VLS can also be formed through the co-expression of NSP5 with VP2. Here, we report that VP2-induced VLS formed in RV species A to J, with notable formation in RVH and RVJ, where NSP2 RVH or RVJ was also recruited into VLSs. The NSP5 C-terminal region in non-RVA is required for association with VP2 and forming VLS. Mutation of conserved VP2-L124 in RVA to alanine disrupts viroplasm formation, impairing RV replication. Equivalent residues within the same predicted VP2 region disrupt VLS formation across non-RVA. We also observed interspecies VLS formation, most notably between the closely related pairs RVA–RVC, RVH–RVJ, and RVD–RVF. Interestingly, substituting the N-terminal region of VP2 from RVB with that of VP2 from RVG supported VLS formation with NSP5 from RVB in avian cells. Elucidating the formation of viroplasms is essential for developing strategies to halt infection across RV species A to J.

**IMPORTANCE:**

Rotaviruses (RV) are a group of viruses classified into species A through J, with species A being the best understood. Other RV species infecting animals and humans are less studied due to limited research tools. In RVA, the virus replicates in specialized compartments called viroplasms formed in the cytoplasm by viral proteins, including NSP5, NSP2, and VP2. In this study, we explored how similar structures, termed viroplasm-like structures (VLS), are formed by proteins of RV species A–J. We found that for all tested RV species, NSP5 and VP2 form VLSs. We also identified key regions in the VP2 protein that are essential for forming these structures. Understanding how viroplasms form across different RV species may help develop new strategies to block infection in humans and animals.

## INTRODUCTION

Rotavirus (RV) is an etiological agent belonging to the *Sedoreoviridae* family and is responsible for severe gastroenteritis and dehydration. According to the International Committee on the Taxonomy of Viruses (ICTV), RVs are currently grouped into nine species, from *Rotavirus alphagastroenteritidis* to *Rotavirus jotagastroenteritidis* (1). For simplicity, the RV species will be termed herewith as A–D and F–J. Although two additional RV species, K and L, have been recently described and recognized by the ICTV (2, 3), the RV species A (RVA) is the prevailing RV species among infants and young children, killing approximately 128,000 children per year, mainly in low- and middle-income countries (4). RVA also has a broad spectrum of strains primarily infecting young mammals like piglets and calves (5, 6). The non-RVA species have been isolated from diverse hosts, including mammals and avians. Outbreaks from RVB, RVC, and RVH are the leading cause of diarrhea among the adult human population in several countries (7–11). In the US, RV infections are the second most common cause of diarrhea in adults after norovirus (12–14). From a veterinary perspective, RV infections significantly impact livestock worldwide. RV accounts for 80% of diarrhea cases in piglets in the USA, Canada, and Mexico, with potential zoonotic implications in humans (15). RV species D, F, and G have only been detected in avian species, affecting the poultry industry by impacting the feed conversion ratio and resulting in substantial economic losses (16). All the information compiled on the RV replication cycle is based on RVA. Studying the replication of non-RVA species is challenging, and as a result, their biology remains poorly understood. The few isolated viruses of non-RVA species do not replicate in tissue culture (17–19), and tools recognizing their specific proteins, like specific antibodies, are unavailable.

RV has 11 double-stranded (ds) RNA genome segments encoding six structural proteins (VP1, VP2, VP3, VP4, VP6, and VP7) and five non-structural proteins (NSP1, NSP2, NSP3, NSP4, and NSP5). In certain RVA strains, genome segment 11, in addition to NSP5, also encodes an out-of-frame protein called NSP6. The RVA virion is a non-enveloped icosahedral triple-layered particle that encloses the 11 genome segments and 12 copies of the replication intermediates, which include RNA-dependent RNA polymerase (RdRp) VP1 and the guanylmethyltransferase VP3, inside a core shell made of 12 decamers of VP2 (*T* = 1) (20, 21). Surrounding the core shell, the middle layer consists of 260 trimers of the structural protein VP6 (*T* = 13), forming transcriptionally active double-layered particles (DLPs) (22, 23). The outer layer is made of trimers of glycoprotein VP7 arranged in icosahedral symmetry (*T* = 13), standing on VP6 trimeric subunits. The spike protein VP4 is anchored in a trimeric formation at each of the fivefold axes of the virion (24–27).

During RVA infection, the external layer is lost after virion internalization, and transcriptionally active DLPs are released into the cytosol (28). The newly released transcripts initiate the synthesis of viral proteins necessary for viral replication. Among those proteins, the nonstructural proteins NSP2 and NSP5 and the structural proteins VP1, VP2, VP3, and VP6 comprise part of the RV viral factories termed viroplasms (29). The viroplasms correspond to electron-dense membrane-less globular cytosolic inclusions where viral genome transcription, replication, and the packaging of the newly synthesized pre-genomic RNA segments into the viral cores occur. The viroplasms are highly dynamic, being able to coalesce between them and migrate to the juxtanuclear region of the cell at later stages post-infection (30–32). Furthermore, despite not yet being well defined, several host factors have been identified as necessary for viroplasm formation and maintenance (33–36). For RVA, the initiation process for viroplasm formation requires a scaffold of lipid droplets that incorporates perilipin-1 (37, 38). The host cytoskeleton, including actin filaments and microtubules (MT), supports the formation and behavior of the viroplasms (31, 39, 40). NSP2 octamers directly associate with MTs, promoting viroplasm coalescence (31, 41–44), while VP2 enables perinuclear motion (31). These characteristics align with viroplasms considered as liquid-liquid phase-separated structures (45). Interestingly, the co-expression of NSP5 with either NSP2 or VP2 leads to the formation of cytosolic inclusions named viroplasm-like structures (VLS), which are morphologically similar to viroplasms but unable to yield viral progeny (30, 31, 46–49). When associating with NSP2 or VP2, NSP5 is primed at serine-67 by the casein kinase 1 alpha, triggering NSP5 hyperphosphorylation (46, 50–52). The NSP5 S67A mutation prevents viroplasm formation (53). The NSP5 phosphorylation is consistent with a trait for recently described liquid-liquid phase separation conditions of the viroplasms (45). NSP5 is not only required for viroplasm formation and virus replication (53–55) but also plays a multifunctional role in the RV life cycle, interacting with NSP6 (49), NSP2 (30), VP1 (56), VP2 (57, 58), and unspecifically with dsRNA (59). These attributes are consistent with its predicted disordered nature (60–62). Interestingly, the C-terminal ordered region (henceforth tail) of NSP5 is needed for its self-oligomerization (49, 50), to associate with other RV proteins (30, 49, 56, 58), and to form the viroplasms (53). NSP5 is sumoylated (63), presumably a prerequisite for interacting with viral or host components. Overall, NSP5 plays a crucial role in the replication of RV.

RVA octameric NSP2 is associated with several enzymatic functions, including nucleoside diphosphate kinase-like activity (64), RNA-helix destabilization (64), and nucleoside triphosphatase activity (42), all of which are consistent with molecular motor properties (42, 65). Moreover, NSP2 phosphorylation and its association have been linked to viroplasm formation and dynamics (30, 43, 66). In this context, NSP2 octamers are directly associated with MTs to promote viroplasm coalescence (31, 41–44). Interestingly, the flexible C-terminal region of NSP2 enhances viroplasm morphology (67) and RNA chaperone activity (41). Notably, NSP2 binds both to VP1 and viral RNA (68, 69), implicating it as a key component of replication intermediates within the viroplasms.

Likewise, the core-shell protein VP2, in addition to its structural role in safeguarding the RVA genome, can activate and regulate the RdRp VP1, allowing for genome replication. VP2 forms asymmetric decameric structures converging in the fivefold axis, which cannot be dissociated (21, 24, 62, 70, 71). Each decameric subunit comprises a main domain of VP2 (residues ~100–880), creating a thin, comma-shaped plate where the unfolded N-terminal domain (NTD) is positioned beneath the decameric five-fold axis (20, 24, 71). Several viral proteins (22, 71–73) and nonspecific single-stranded RNA (ssRNA) (74) interact with VP2, primarily to facilitate association with the NTD. These interactions are closely linked to the core-shell structure and genome replication. Additionally, VP2 serves as a key component in forming viroplasms and, when co-expressed with NSP5, produces VLS (31, 46, 58, 75). In this context, the VLSs induced by VP2 are dynamic as they migrate to the perinuclear region (31). Furthermore, the highly conserved L124 of VP2 in RVA is crucial for its association with NSP5. When L124 is mutated to alanine, VP2 L124A disrupts viroplasm morphology, rendering RV replication incompetent (58). Recently, it has been suggested that VP2 may have further roles early post-infection due to its interaction with NSP2, which prevents its spontaneous oligomerization and sumoylation, thereby enhancing the ability of VP2 to interact with other proteins (31, 63).

We recently examined whether NSP5 and NSP2 from non-RVA can form VLSs (76). The co-expression of these proteins produced globular VLSs in RVA, RVB, RVD, RVF, RVG, and RVI, while RVC formed filamentous VLSs. No VLSs formed with NSP5 and NSP2 from RVH and RVJ. NSP5 from all species oligomerized via its tail and, except for RVJ, interacted with its corresponding NSP2. Interspecies VLSs formed between related species (B/G and D/F). Notably, VLSs were restored in RVH and RVJ by swapping their NSP5 tails with those of RVA.

In this study, we characterized the formation of VLS supported by the co-expression of NSP5 and VP2 across RV species A–J. We determined that the NSP5 tail is crucial for both VLS formation and its interaction with VP2 in all RV species tested. A point mutation to alanine of a conserved amino acid residue in VP2 disrupts VLS formation. Heterologous VLS formation was observed between closely related RV species pairs: A and C, B and G, D and F, as well as H and J. Additionally, we demonstrated that the unstructured N-terminal region of VP2 is necessary for VLS formation.

## RESULTS

### Biophysical features of VP2 in RV species A–J

**Fig 1:**
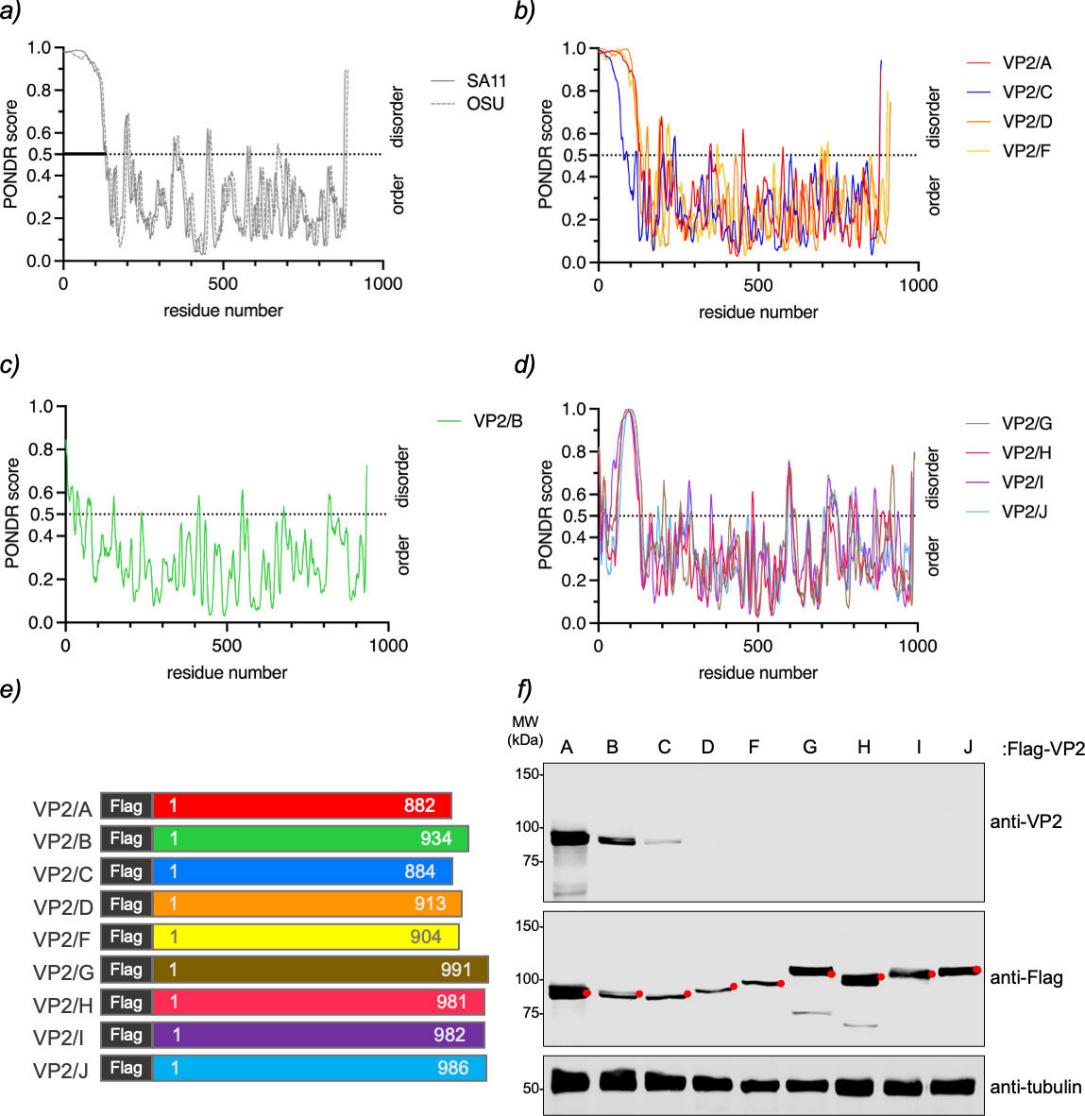
Instrinsically disordered protein (IDR) and expression of VP2 in species A–J. Plots comparing the IDR prediction of VP2 from RVA strains OSU and SA11 (**a**), RVA (strain SA11), RVC, RVD, and RVF (**b**), RVB (**c**), and RVG-RVJ (**d**). (**e**) Schematic representation of VP2 from RV species A–J, fused to a Flag tag at its N-terminal region. (**f**) Immunoblotting of MA104 cell extracts expressing Flag-VP2 from RV species A–J. The membrane was incubated with guinea pig anti-VP2 (top) and mouse mAb anti-Flag (middle). Anti-tubulin was used as a loading control (bottom). The red dot indicates the predicted molecular weight of the recombinant proteins.

We recently demonstrated that the replication mechanism of non-RVA species can be investigated by extrapolating the roles of NSP5 and NSP2 from RVA to their orthologs in other RV species (76). In this context, it is known that RVA forms VLS upon co-expression of NSP5 with VP2 (46, 58, 77). This prompted us to investigate whether VP2 from non-RVA species might similarly contribute to VLS formation when co-expressed with its cognate NSP5. To begin, we identified available VP2 open reading frames for RV species A–J in the NCBI database, matching each with its cognate NSP5 and NSP2 sequences as previously described (76). However, RVB, RVC, and RVI complete VP2 sequences were unavailable, so we substituted strains with higher homology (Table 1) (76). The VP2 proteins vary in length across RV species, with differences of up to 109 amino acids. RVA has the shortest VP2 (882 amino acids), and RVG has the longest (991 amino acids; Table 1). Sequence analysis revealed high diversity among VP2 proteins from species A to J compared to our model strain, RVA (simian strain SA11). The most similar sequence was from RVF (68.51% similarity), and the most divergent was RVJ (35.28% similarity; Table 1). Consistent with previous findings that the N-terminal domain of VP2 RVA is unfolded (residues ~1–100 for type A and ~1–80 for type B) (20, 21, 24, 71), the PONDR analysis also predicted a highly disordered N-terminal region in the VP2 sequences of RVA model strains SA11 and OSU (Fig. 1a). Similar disordered N-terminal regions were predicted in VP2 from most non-RVA species (Fig. 1b and d), except for RVB, which lacked this feature (Fig. 1c). The predicted disordered regions in VP2 N-termini varied among species: RVA, RVC, RVD, and RVF showed completely disordered N-terminal domains (Fig. 1b), while RVG, RVH, RVI, and RVJ showed partially disordered regions, characterized by few ordered residues at the extreme N-terminus, followed by a disordered region of approximately 50 residues (Fig. 1d). We also used AlphaFold3 to compare the predicted folding of VP2 across different RV species. The predicted dimeric structure of VP2 RVA closely matched the previously experimental structure (Fig. S1, RVA) (71). For VP2 of non-RVA species, AlphaFold3 predicted similar overall structures, especially in the apical, central, and dimerization regions (Fig. S1). As expected for disordered domains, AlphaFold3 showed reduced confidence in the N-terminal regions across all analyzed RV species (data not shown). Accordingly, we designed a series of plasmids encoding the VP2 open reading frame from RV species A–J, each tagged with a Flag epitope at the N-terminus (Fig. 1e) (58). Lysates from MA104 cells expressing these Flag-VP2 constructs were assessed by immunoblotting using a polyclonal anti-VP2 antibody raised against RVA strain SA11 (56). This antibody detected VP2 from RVA and, with lower affinity, VP2 from RVB and RVC, suggesting antigenic homology for VP2 among these RV species (Fig. 1f, upper panel). Subsequent probing with an anti-Flag antibody recognized VP2 from all tested RV species, with migration patterns corresponding to their predicted molecular weight (Table S1).

**TABLE 1:** NSP5 and VP2 protein features of the RV species analyzed in this study

RV species	RV protein	Host	Strain	GenBank accession no.	Amino acid length	Predicted MW (kDa)	Similarity (%) to RVA^*a*^
RVA	NSP5	Simian	SA11	BAW94621	198	21.72	100.00
	VP2	Simian	SA11	LC178565.1	882	102.7	100.00
RVB	NSP5	Human	CAL-1	AF206724	170	19.77	42.99
	VP2	Human	Bang 373	NC_021545.1	934	105.9	36.62
RVC	NSP5	Porcine	12RO21	KP982878	210	23.21	44.29
	VP2	Human	Bristol	NC_007546.1	884	101.7	68.02
RVD	NSP5	Chicken	05 V0049	NC_014521	195	22.26	37.79
	VP2	Chicken	05 V0049	NC_014512.1	913	106.2	64.19
RVF	NSP5	Chicken	03 V0568	NC_021629	218	24.38	45.54
	VP2	Chicken	03 V0568	NC_021626.1	904	104.5	68.51
RVG	NSP5	Chicken	03 V0567	JQ920012	181	20.84	32.72
	VP2	Chicken	03 V0567	NC_021580.1	991	112.7	36.50
RVH	NSP5	Pig	SP-VC36	MT644988	180	20.36	35.85
	VP2	Pig	SP-VC36	MT644972.1	981	112	37.06
RVI	NSP5	Raccoon dog	SD-MO2	OM451078	157	17.72	33.66
	VP2	Dog	KE135	NC_026826.2	982	110.6	35.70
RVJ	NSP5	Bat	BO4351	NC_055273	165	18.48	30.99
	VP2	Bat	BO4351	NC_055265.1	986	112.4	35.28

^*a*^ Pairwise similarities were obtained by global alignment (Needleman-Wunsch) using matrix BLOSUM62, Gap open penalty of 10.0 and extend penalty of 1.0.

### VLS formation by co-expression of NSP5 with VP2 across RV species A–J

**Fig 2:**
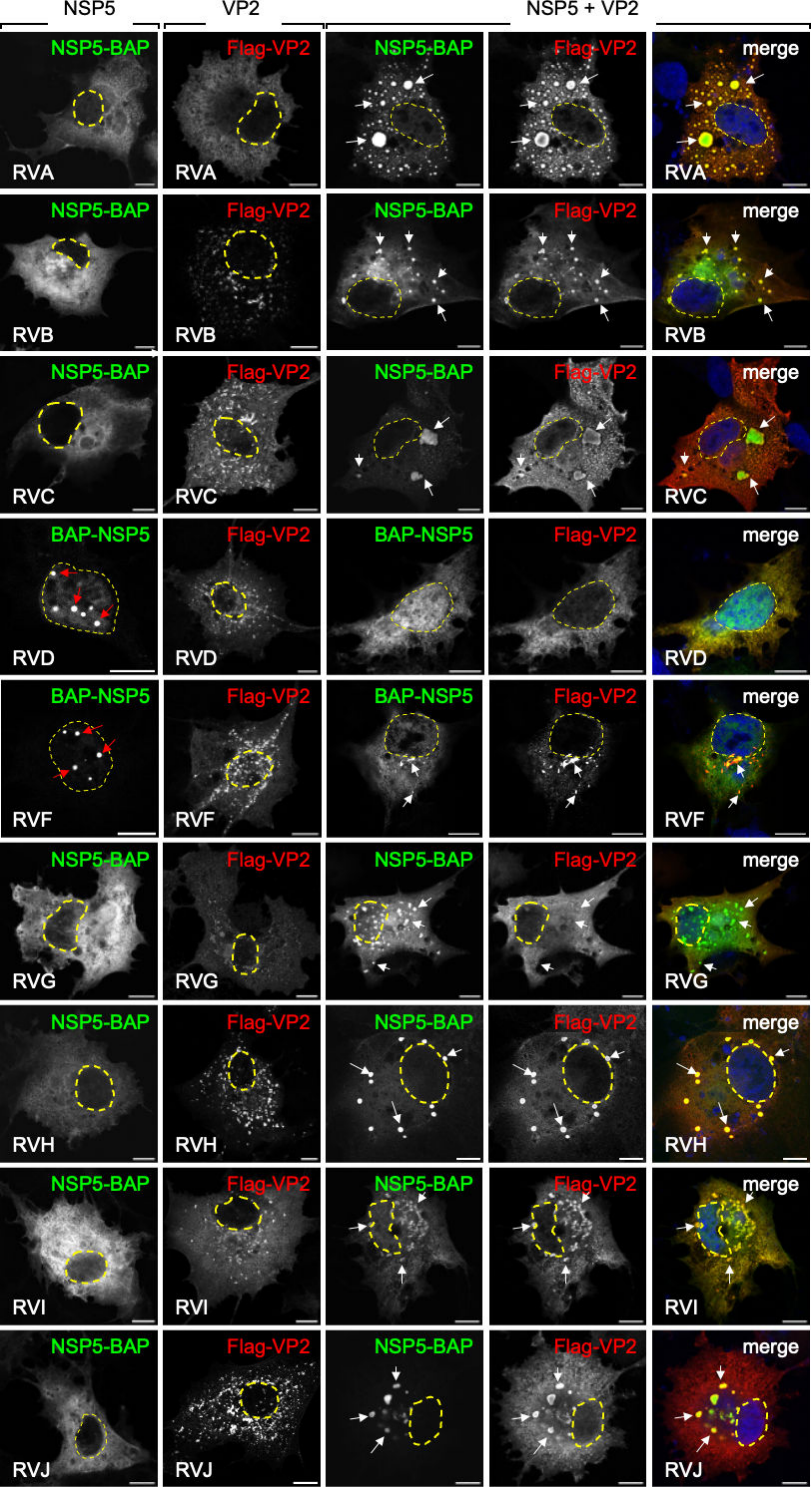
Characterization of VLS formation through co-expression of NSP5-BAP and Flag-VP2 across RV species A–J. Immunofluorescence images of MA/cytBirA cells expressing NSP5-BAP (RVA-RVC and RVG-RVJ) or BAP-NSP5 (RVD and RVF) alone (first column), Flag-VP2 (RVA-RVJ) alone (second column), and the co-expression of both proteins (third, fourth, and fifth columns). At 16 hpt, the cells were fixed and stained to detect NSP5 with Streptavidin (green) and VP2 (anti-Flag, red). A merged image is shown in the fifth column. Nuclei were stained with 4′,6-diamidino-2-phenylindole (DAPI) (blue). The scale bar is 10 µm. The white and red arrows point to globular VLS and nuclear inclusions, respectively. The yellow discontinuous line marks the nucleus as determined by DAPI staining.

Next, we investigated whether biotin acceptor peptide (BAP)-tagged NSP5 (76) co-expressed with their cognate Flag-VP2 protein supports the formation of VLS in RV species A–J. Of note, VLSs are visualized by colocalization of the signals of NSP5 with NSP2 or VP2 in globular cytosolic inclusions (39, 46, 48, 58, 76, 77). In the first instance (Fig. 2; Fig. S2), the proteins were expressed in mammalian MA/cytBirA cells and fixed at 16 h post-transfection (hpt). VLS formation was monitored by immunofluorescence for the detection of NSP5 fused to BAP tag (streptavidin-Dylight 488, green) and Flag-VP2 (mAb anti-Flag followed by secondary antibody conjugated to Alexa 594, red), respectively. As expected (58), NSP5-BAP and Flag-VP2 of RVA colocalized, forming globular cytosolic inclusions corresponding to VLS. Similarly, the co-expression of NSP5-BAP and Flag-VP2 of RVB, RVC, RVF, RVG, RVH, RVI, and RVJ also led to the formation of globular VLSs. However, the co-expression of these proteins in RVD did not result in VLS formation. As previously described (76), BAP-NSP5 of RVD and RVF formed globular inclusions in the nuclei.

**Fig 3:**
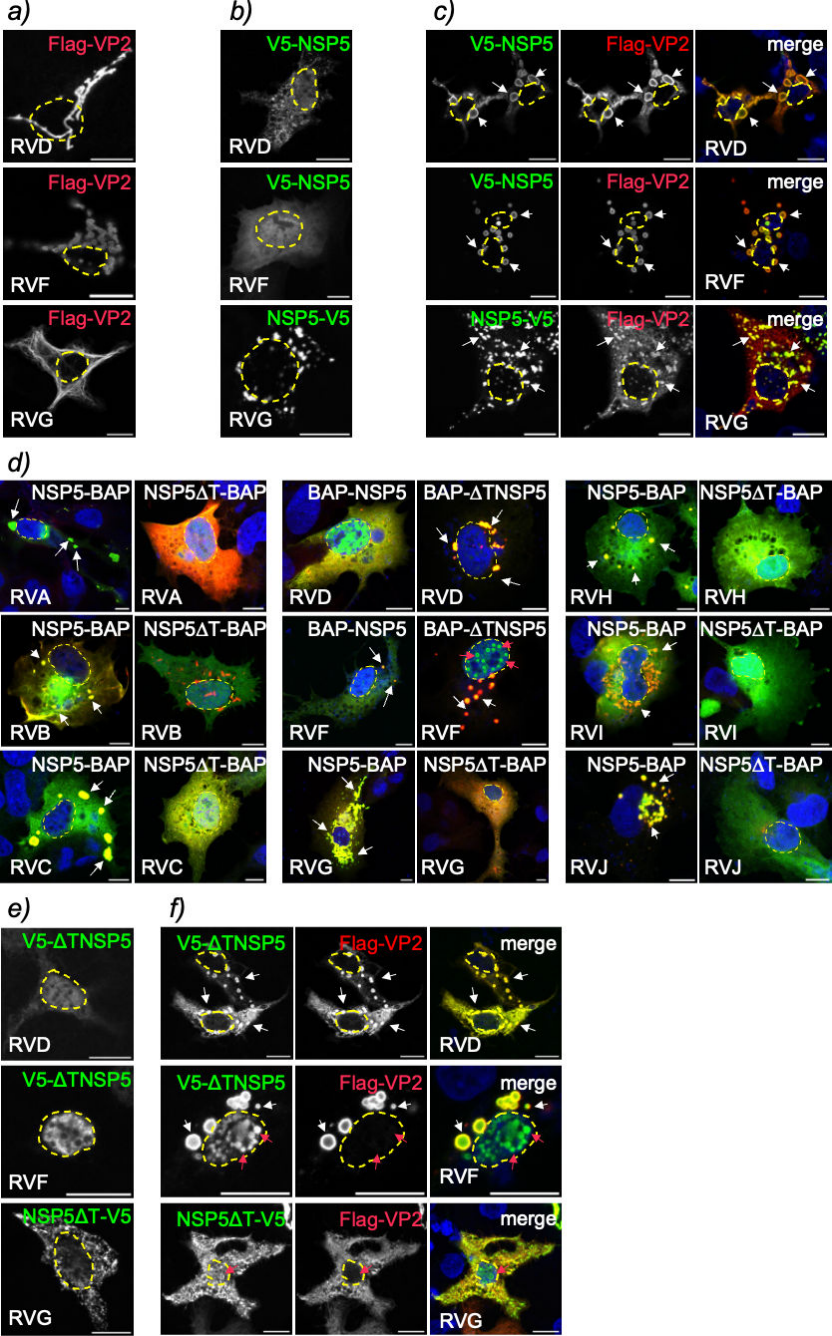
The role of the NSP5 tail in the formation of VLS induced by VP2 in RV species A–J. Immunofluorescence images of chicken LMH cells expressing (**a**) Flag-VP2 alone, (**b**) NSP5 fused to a V5 tag alone, and (**c**) their co-expression in RVD, RVF, and RVG. (**d**) Merged immunofluorescence images of MA/cytBirA cells co-expressing Flag-VP2 with either NSP5 (first, third, and fifth columns) or NSP5∆T (second, fourth, and sixth columns), both fused to a BAP tag. Immunofluorescence images of LMH cells expressing a tail deletion of NSP5 fused to a V5 tag from RV species D, F, and G alone (**e**) or co-expressed with its respective Flag-VP2 (**f**). At 16 hpt, the cells were fixed and immunostained to detect NSP5 or NSP5∆T (StAv-Dylight 488, green or anti-V5-Dylight 488, green) and VP2 (anti-Flag, red). Nuclei were stained with DAPI (blue). The scale bar indicates 10 µm. The white, red, and yellow arrows point to globular VLS, nuclear inclusions, and aggregated proteins, respectively. The discontinuous yellow lines outline the nucleus as identified by DAPI staining.

Since RVD, RVF, and RVG were originally isolated from avian hosts, we hypothesized that the host cellular environment might influence the folding and interaction behavior of NSP5 and VP2, thereby affecting VLS formation. To test this, we expressed V5-tagged NSP5 and Flag-VP2 in LMH chicken epithelial cells and monitored VLS formation via immunofluorescence. Of note, V5-NSP5 was used instead of BAP-NSP5 because LMH cells lack the cytosolic BirA. In this context (Fig. 3a), the expression of Flag-VP2 alone from RVD, RVF, and RVG led to filamentous structures for RVD and RVG, while Flag-VP2/RVF formed globular structures, distinct from the diffuse cytosolic aggregates observed in MA/cytBirA cells for these species. As expected (76), V5-NSP5 from RVD and RVF appeared diffusely distributed in the cytosol, whereas NSP5-V5 from RVG formed globular cytosolic inclusions (Fig. 3b). Furthermore, deletion of the N-terminal “tail” region of NSP5 from RVD and RVF resulted in its localization to both the cytosol and the nucleus. In LMH cells, co-expression of V5-tagged NSP5 with Flag-VP2 from RV species D, F, and G resulted in the formation of cytosolic globular VLSs (Fig. 3c).

We previously described that the predicted ordered region of NSP5, referred to as the “tail,” is located at the N-terminus in RVD and RVF and at the C-terminus in RVA, RVB, RVC, RVG, RVH, RVI, and RVJ. This tail has been shown to play a predominant role in VLS formation with NSP2 (76). Moreover, deletion of the tail region (NSP5∆T) has been shown to impair VP2-induced VLS formation in RVA (58). We investigated whether this deletion would similarly disrupt VLS formation in non-RVA species. To address this (Fig. 3d), we co-expressed Flag-VP2 (red) with either full-length NSP5 or its tail-deleted version (NSP5∆T), both BAP-tagged (green), in MA/cytBirA cells. As expected (58), co-expression of Flag-VP2/A with NSP5∆T-BAP/A impaired VLS formation. Similar impairments were observed for RVB, RVC, RVH, RVG, RVI, and RVJ, following deletion of the NSP5 tail. Surprisingly, BAP-∆TNSP5/D acquired the ability to form VLS with its cognate Flag-VP2. Although BAP-∆TNSP5/F alone formed nuclear inclusions (Fig. S3 [76]), its co-expression with Flag-VP2/F led to the formation of numerous and enlarged cytosolic VLS, as well as nuclear globular inclusions. In LMH cells (Fig. 3e and f, upper and middle rows), the co-expression of V5-∆TNSP5/D and ∆TNSP5/F with their corresponding Flag-VP2 also supported VLS formation, consistent with results in mammalian cells. In contrast (Fig. 3e and f, bottom row), co-expression of NSP5∆T-V5/G with Flag-VP2/G impaired VLS formation and resulted in the accumulation of nuclear globular inclusions, likely composed of NSP5∆T-V5/G alone.

### The NSP5 tail plays a crucial role in its interaction with VP2

**Fig 4:**
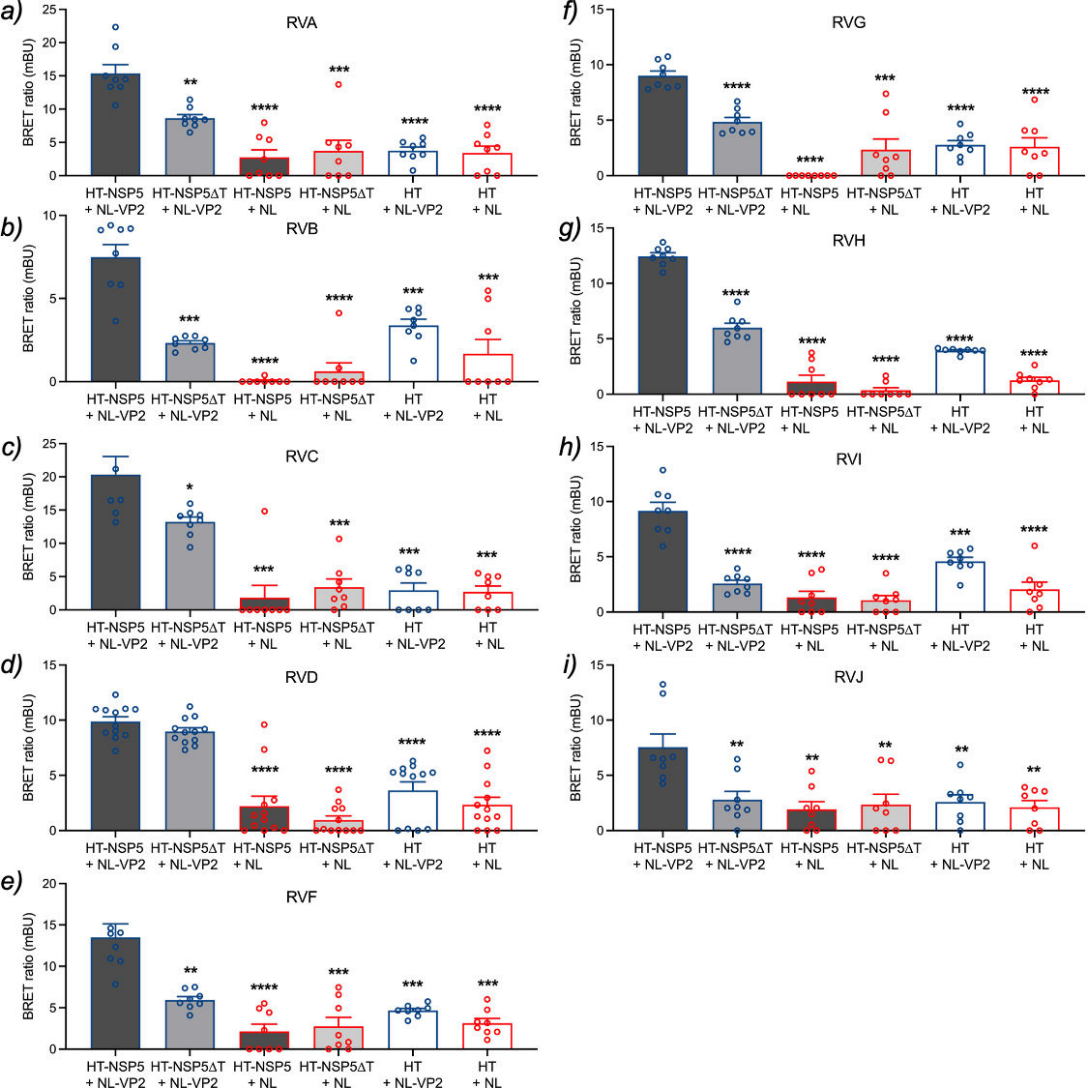
Quantification of VP2 and NSP5 association across RV species A–J. A BRET assay was conducted to examine the association of NanoLuc luciferase (NL) fused to VP2 with HaloTag (HT) fused to full-length NSP5 or NSP5∆T for RV species A (**a**), B (**b**), C (**c**), D (**d**), F (**e**), G (**f**), H (**g**), I (**h**), and J (**i**). HEK-293T cells were transfected with the indicated pairs for 16 h, then incubated with HaloTag 618 ligand substrate for 6 h, followed by the addition of Nano-Glo substrate. Luminescence controls for each protein were included. The result corresponds to the mean ± SEM of three independent experiments. The data were compared to HT-NSP5 with NL-VP2 couple using Brown-Forsythe and Welch ANOVA tests, (*), *P* < 0.05; (**), *P* < 0.01; (***), *P* < 0.001; and (****), *P* < 0.0001.

Given that the ordered region of NSP5 plays a critical role in VLS formation in most RV species studied, we investigated whether this region is also required for the direct interaction between NSP5 and VP2. Previous studies using pull-down and tripartite green fluorescent protein (GFP) assays demonstrated that NSP5∆T disrupts its association with VP2 in RVA (58). In this study, we developed a bioluminescence resonance energy transfer (BRET) assay to monitor NSP5-VP2 interactions in living cells. This system uses NanoLuc luciferase (NL) fused to VP2 (NL-VP2) as the energy donor and HaloTag fused to NSP5 (HT-NSP5) as the fluorescent acceptor. The BRET signal, which arises from energy transfer between NL and HT when the proteins are in close proximity, serves as a quantitative readout of interaction (Fig. S4a). First, we validated the assay by co-expressing NL-VP2 and NSP5-HT from RVA. As expected, these proteins showed a significant interaction, with BRET values markedly higher (*P* < 0.000001) than control pairs (HT-NSP5 + NL and HT + NL-VP2; Fig. 4a). In contrast, interaction was significantly reduced when NL-VP2 was co-expressed with tail-deleted version HT-NSP5∆T. We then extended this assay to RV species B through J, constructing NL-VP2 and HT-NSP5 fusion proteins to each species (Fig. S4b through f). All tested VP2-NSP5 showed significant interaction signals (Fig. 4b through i). However, when the NSP5 tail was deleted, the interaction with NL-VP2 was significantly impaired for all RV species tested, except RVD, where the interaction was retained. Similar results were obtained through co-immunoprecipitation of cell lysates co-expressing Flag-VP2 with either full-length NSP5 or its tail-deleted version, both fused to a BAP tag, across RV species A to J, thereby validating the BRET assay (Fig. S5).

### VP2 phylogenetic analysis and structural localization of conserved critical residues involved in VLS formation

**Fig 5:**
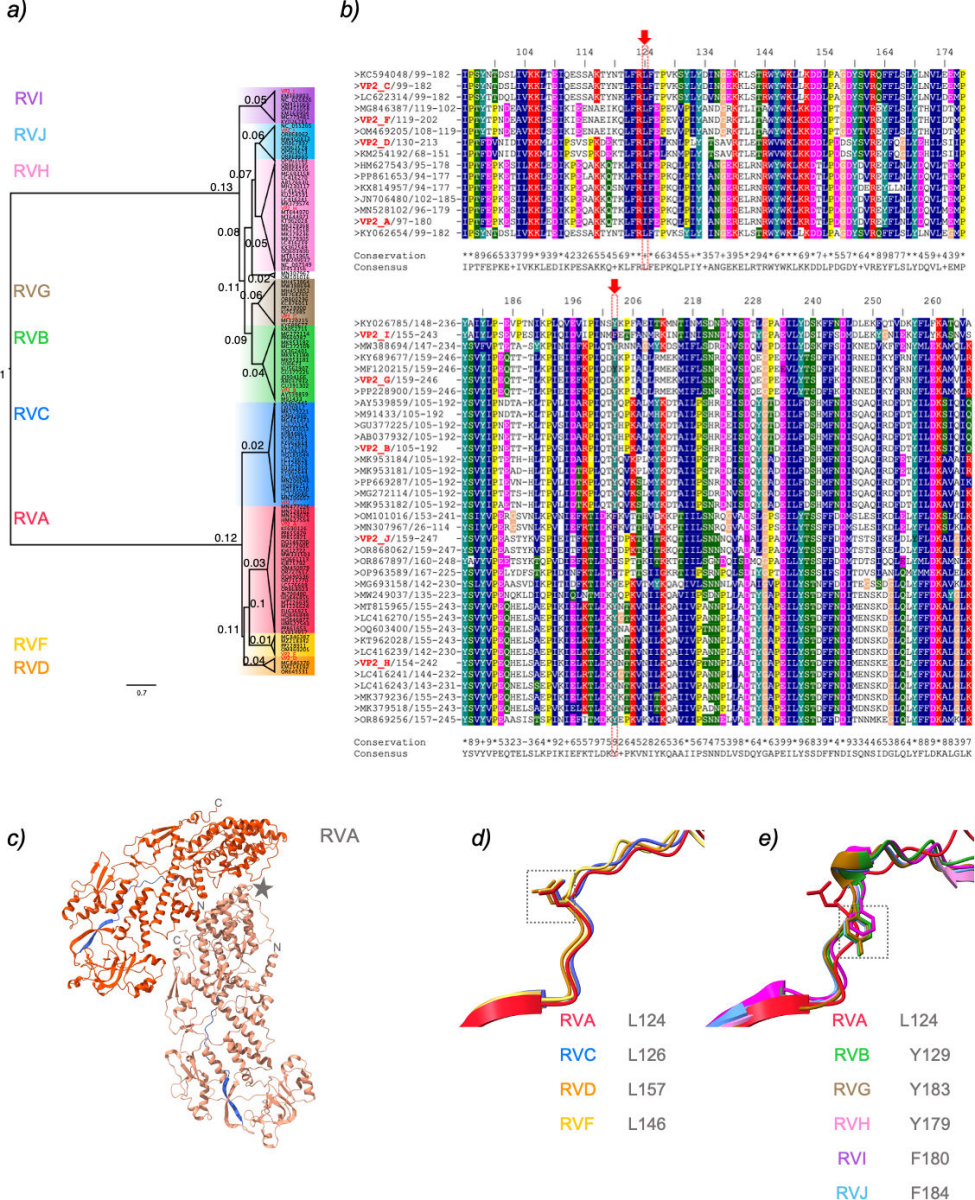
Sequence likelihood of VP2 from RV species A–J. (**a**) Maximum likelihood tree illustrating phylogenetic relationships among VP2 of RV species A–J. The red label corresponds to the CDS used in this study, followed by a letter indicating its RV species. Each RV species has a colored panel: RVA, red; RVB, green; RVC, blue; RVD, orange; RVF, yellow; RVG, brown; RVH, pink; RVI, purple; and RVJ, light blue. The scale is 0.7 substitutions per nucleotide. (**b**) Multiple sequence alignment of VP2 from RV species A–J based on the VP2 phylogenetic tree. GenBank accession numbers are shown. Conserved residues are labeled according to the ClustalX classification, with blue indicating hydrophobic, red positively charged, pink negatively charged, green polar, light orange glycine, yellow proline, cyan aromatic, and white unconserved residues. The first and last residues of each RV protein region are marked. Red arrows point to conserved leucine (L) in RV species A, C, D, and F or aromatic residues (F or Y) in RV species B, G, H, I, and J, respectively. (**c**) AlphaFold3 prediction of dimeric VP2 of RVA (monomer type A, dark orange; and monomer type B, light orange). The region from 97 to 180 is highlighted in blue for both monomers. A gray star indicates the fivefold axes. N- and C-termini of each monomer are labeled. (**d**) Superimposition of VP2 region 97–180 of RVA (red) with RV species C (blue), D (orange), and F (yellow) on the left. A gray dashed open box highlights conserved leucine. (**e**) Superimposition of VP2 region 97–180 of RVA (red) with RV species B (green), G (brown), H (pink), I (purple), and J (light blue) is shown on the right. Conserved aromatic residues Y or F are marked by a gray dashed open box. The predicted local distance difference test scores are >70 for all model predictions.

We analyzed the evolutionary relationship of VP2 across RV species A–J (Fig. 5a) by comparing their coding sequences (CDS) available in public databases. This allowed us to identify RV species pairs sharing common ancestors. Similar to what has been reported for NSP5 and NSP2 (76), VP2 phylogeny revealed two major groups, one comprising RV species A, C, D, and F, and another including RV species B, G, H, I, and J. Within this framework, RVA is most closely related to RVC, RVF to RVD, RVB to RVG, and RVH to RVJ. RVI appears to be most distantly related but shows a closer affinity to RVH and RVJ.

We previously reported that a highly conserved leucine residue at position 124 (L124) in VP2 of the RVA strain SA11 is essential for viroplasm and VLS formation as well as for its association with NSP5 and efficient RV replication (58). Equivalent leucine residues were also identified in other RV species, specifically L126 in RVC, L157 in RVD, and L146 in RVF (Fig. 5b, top panel). However, no conserved leucine residues in corresponding positions were found in RVB, RVG, RVH, RVI, and RVJ (data not shown). To explore structural conservation, we mapped the RVA VP2 L124 region onto the known tertiary structure of RVA VP2 from the RRV strain (78), which spans amino acid residues 94–180 (Fig. 5c, blue region). L124 was found within a loop that precedes a beta-sheet. Using AlphaFold3, we superimposed the VP2 RVA tertiary structure with predicted VP2 structures of RV species B through J. Consistent with our sequence alignment, the predicted structures of RVC (L126), RVD (L157), and RVF (L146) showed complete overlap with RVA L124 (Fig. 5d). Furthermore, the predicted VP2 structures of RVB, RVG, RVH, RVI, and RVJ also overlapped with RVA VP2 region spanning residues 97–180 (Fig. 5e), which also includes a loop preceding a beta-sheet. Interestingly, sequence alignment of this loop revealed conserved aromatic residues, tyrosine or phenylalanine, at positions corresponding to RVA L124. We identified Y129 in RVB, Y183 in RVG, Y179 in RVH, F180 in RVI, and F184 in RVJ (Fig. 5b, bottom panel), all of which align with the same loop region as L124 in VP2 RVA.

### Conserved VP2 residue is essential for VLS formation

**Fig 6:**
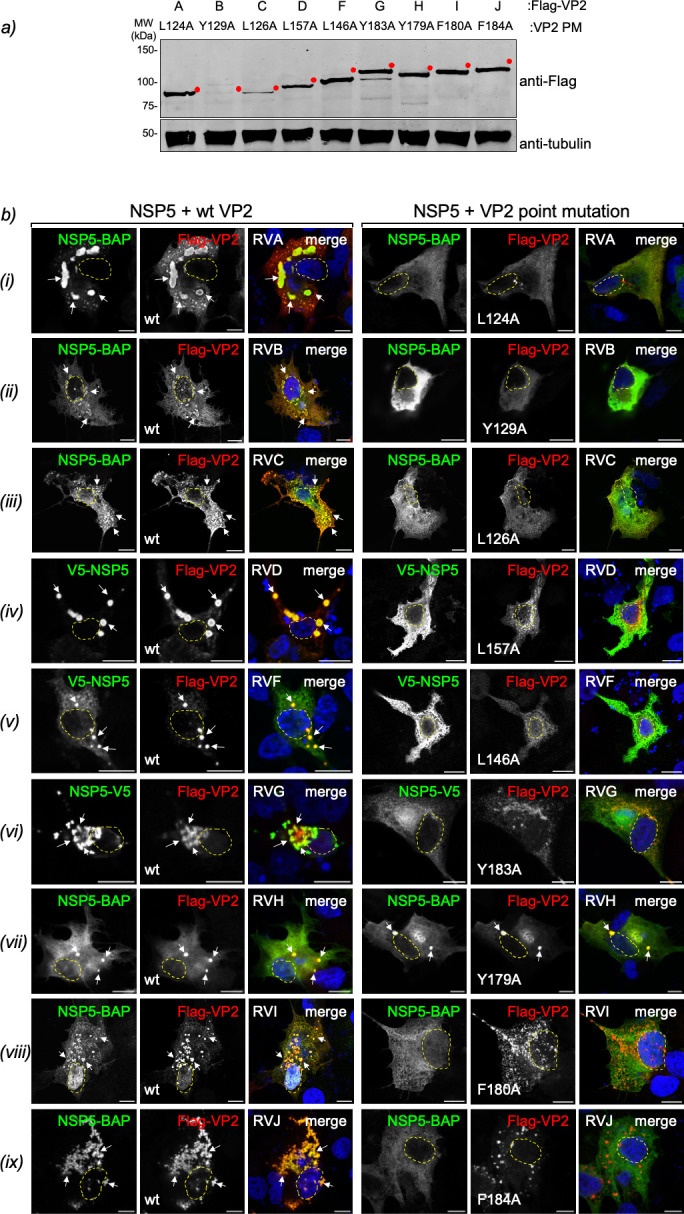
Point mutations in conserved residues of VP2 for RV species A–J impair VLS formation. (**a**) Immunoblotting of cellular lysates from MA104 cells expressing Flag-VP2 of RV species A–J with the indicated point mutation. The membrane was incubated with mouse mAb anti-Flag to detect Flag-VP2 (top) and mouse mAb anti-tubulin (bottom) to detect alpha-tubulin as a loading control. The red dot indicates the predicted molecular weight. (**b**) Immunofluorescence images comparing VLS formation in cells co-expressing NSP5 with either wtVP2 (left) or VP2 containing the indicated point mutation (right) of RV species A-J. At 16 hpt, cells were fixed and co-stained to detect NSP5-BAP (StAv, green) or V5 tag fusion to NSP5 (anti-V5, green, RV species D, F, and G) with Flag-VP2 (anti-Flag, red). The third and sixth columns show merged images. The scale bar is 10 µm. VLS formation was observed in MA/cytBirA cells for RV species A (i), B (ii), C (iii), H (vii), I (viii), and J (ix), while LMH cells were used for detection in RV species D (iv), F (v), and G (vi). The white arrows point to globular VLS, and nuclei are outlined with dashed yellow lines as determined by DAPI staining.

We hypothesized that a conserved residue in the VP2 protein of RV species B–J is critical for VLS formation, similar to the role of L124 in RVA. Supporting this, a point mutation substituting L124 with a non-bulky amino acid like alanine (L124A) was previously shown to impair VLS formation (58). To test this hypothesis, we generated Flag-VP2 constructs with alanine substitutions at the conserved residues across RV species A–J and expressed them in MA104 cells. These mutant proteins migrated at their predicted molecular weights (Fig. 6a), although Flag-VP2(Y129A) from RVB exhibited weak expression despite proper migration. VP2 point mutations did not alter protein folding, as shown by overlapping AlphaFold3 predictions with wild type (wt) VP2 (Fig. S6a), and both wt and mutant Flag-VP2 displayed identical proteinase K cleavage patterns (Fig. S6b).

To assess VLS formation, we performed immunofluorescence microscopy in MA/cytBirA cells co-expressing NSP5-BAP with either wt Flag-VP2 or Flag-VP2 point mutant from RVA, RVB, RVC, RVH, RVI, and RVJ (Fig. 6b, rows i, ii, iii, vii, viii, and ix). As previously reported (58), co-expression of NSP5-BAP with RVA Flag-VP2 (L124A) failed to support VLS formation (Fig. 6b, row i). Similarly, alanine substitutions in the VP2 proteins of RVB, RVC, RVI, and RVJ also impaired VLS formation when co-expressed with their respective NSP5-BAPs, in contrast to the robust VLS formation observed with the corresponding wt proteins. Interestingly, Flag-VP2 (Y179A) from RVH retained the ability to support VLS formation when co-expressed with RVH NSP5-BAP (Fig. 6b, row vii).

For avian RV species RVD, RVF, and RVG, the corresponding Flag-VP2 point mutants were tested in LMH cells to provide a more suitable host environment (Fig. 6b, rows iv, v, and vi). While the co-expression of wt Flag-VP2 with its cognate NSP5 fused to V5 supported VLS formation in these RV species, the respective alanine mutants, L157A (RVD), L146A (RVF), and Y183A (RVG), failed to form VLSs.

### VLS morphology is modulated by NSP5, NSP2, and VP2

**Fig 7:**
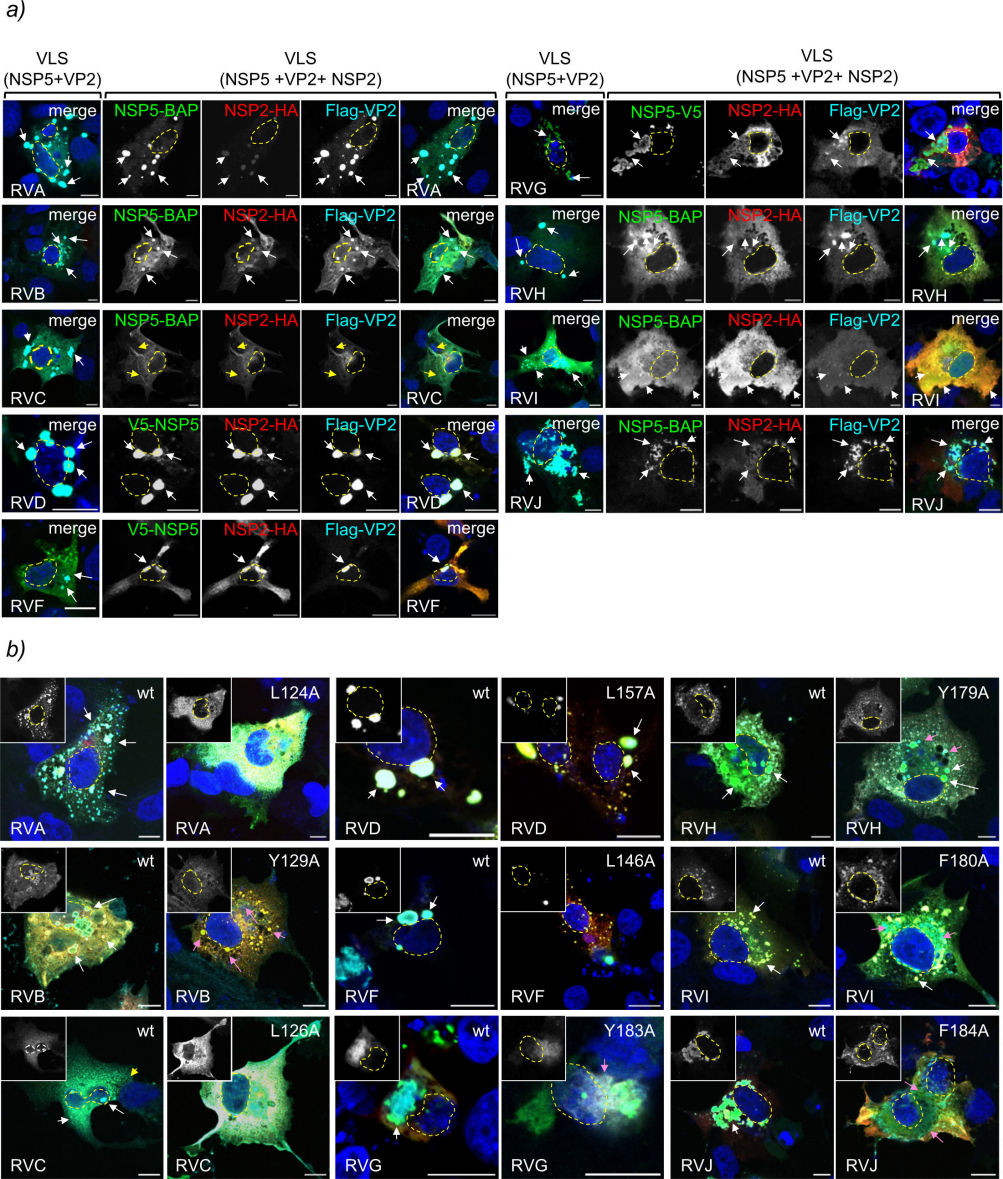
Morphology of VLS composed of NSP5 with VP2 and NSP2 across RV species A–J. (**a**) Immunofluorescence images of cells co-expressing BAP-tagged or V5-tagged NSP5 with Flag-VP2 in the absence and presence of NSP2-HA for RV species A–J. At 16 hpt, the cells were fixed and stained to detect BAP-tagged NSP5 (RVA, RVB, RVC, RVH, RVI, and RVJ; StAv, green) or V5-tagged NSP5 (RVD, RVF, and RVG; anti-V5, green), NSP2-HA (anti-HA, red), and Flag-VP2 (anti-Flag, cyan). Nuclei were stained with DAPI (blue). The scale bar is 10 µm. White arrows point to globular VLSs. Nuclei are outlined with a yellow dashed line based on DAPI staining. (**b**) Immunofluorescence images comparing VLS composed of BAP-tagged or V5-tagged NSP5, NSP2-HA, and Flag-VP2 wt or its corresponding point mutation. The cells were fixed at 16 hpt and stained for the detection of BAP-tagged NSP5 (RVA, RVB, RVC, RVH, RVI, and RVJ; StAv, green) or V5-NSP5 (RVD, RVF, and RVG; anti-V5, green), NSP2-HA (anti-HA, red), and Flag-VP2 (anti-Flag, cyan). Nuclei were stained with DAPI (blue). The scale bar is 10 µm. The images show the merged view. The top left corner of each shows Flag-VP2 immunostaining. White, yellow, and pink arrows indicate globular VLS, filamentous VLS, and dispersed VP2, respectively. Nuclei are outlined with a yellow dashed line based on DAPI staining. In both experiments, VLS formation was observed in MA/cytBirA cells for RVA, RVB, RVC, RVH, RVI, and RVJ, while LMH cells were used for RVD, RVF, and RVG.

We previously reported that the co-expression of cognate NSP5 with NSP2 leads to the formation of globular VLSs in RVA, RVB, RVD, RVG, and RVI, while RVC forms filamentous VLSs and RVH and RVJ fail to form VLSs (Table 2) (76). In this study, we observed that co-expression of NSP5 with VP2 resulted in globular VLS formation in all RV species tested. To assess whether NSP2 influences the morphology of VP2-induced VLSs (Table 2), we compared the morphology of VLS formed by the co-expression of NSP5 and VP2, VLS (NSP5 + VP2), with those formed by the co-expression of NSP5, NSP2, and VP2, VLS (NSP5 + VP2 + NSP2; Fig. 7a). We found that the addition of NSP2 led to globular VLS morphology in all RV species, with the exception of RVC, which retained a filamentous morphology. Notably, in this condition, VLS (NSP5 + VP2 + NSP2) facilitated the recruitment of NSP2 in RVH and RVJ.

**TABLE 2:** Summary of VLS formation through the co-expression of NSP5 with NSP2, VP2, or both^*f*^

RV species	VLS (NSP5 + VP2)				VLS (NSP5 + NSP2) formation^*c*^		Morphology VLS (NSP5 + VP2 + NSP2)
	VLS formation^*a*^		NSP5-VP2 interaction^*b*^				
	NSP5	NSP5∆T	NSP5	NSP5∆T	NSP5	NSP5∆T	
RVA	+ (globular)	−	+	−	+ (globular)	−	Globular
RVB	+ (globular)	−	+	−	+ (globular)	−	Globular
RVC	+ (globular)	−	+	−	+ (filamentous)	−	Filamentous
RVD	+ (globular)^*d*^	+ (globular)	+	+	+ (globular)	+ (globular)	Globular
RVF	+ (globular)	+ (globular)^*e*^	+	−	+ (globular)	+ (globular)^*e*^	Globular
RVG	+ (globular)	−	+	−	+ (globular)	−	Globular
RVH	+ (globular)	−	+	−	−	−	Globular
RVI	+ (globular)	−	+	−	+ (globular)	−	Globular
RVJ	+ (globular)	−	+	−	−	−	Globular

^*a*^ Confirmed by co-immunostaining for the detection of both VLS components, NSP5 and VP2 (transfection ratio 1:2 = NSP5:VP2).

^*b*^ Determined by BRET assay.

^*c*^ Described by Lee et al. (76).

^*d*^ Forms VLS only in chicken epithelial LMH cells.

^*e*^ Forms nuclear inclusions.

^*f*^ +, positive; −, negative.

We also investigated the impact of VP2 point mutations on VLS(NSP5 + VP2 + NSP2) morphology (Fig. 7b). As previously shown by Buttafuoco et al. (58), the Flag-VP2(L124A) disrupted VLS(NSP5 + VP2 + NSP2) in RVA. Similarly, the corresponding VP2 point mutations in other RV species impaired VLS integrity. This was evident in RVD and RVF, where small, punctate VLSs formed lacking detectable VP2, and in RVB, RVG, RVH, and RVI, where VLSs appeared irregular, and VP2 was dispersed throughout the cytosol. Strikingly, VLS formation was completely abolished in RVC and RVJ, resulting in the loss of their characteristic filamentous and globular morphologies, respectively.

### Heterologous formation of VP2-induced VLSs among RV species

**Fig 8:**
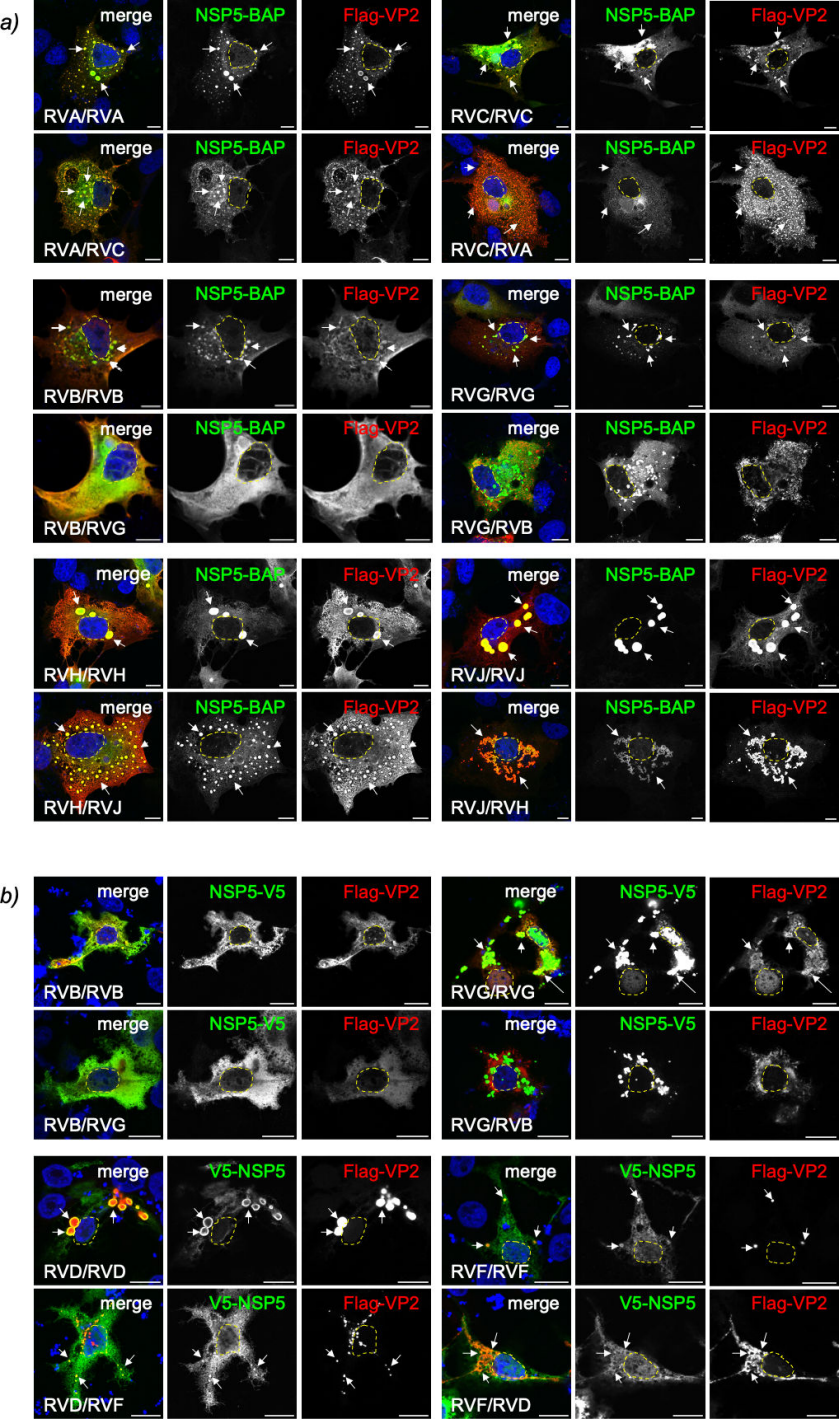
Heterologous formation of VLS by NSP5 and VP2 from closely related RV species. (**a**) Immunofluorescence images of MA/cytBirA cells co-expressing BAP-NSP5 and Flag-VP2 from closely related RVA and RVC (top), RVB and RVG (middle), and RVH and RVJ (bottom) in all indicated combinations. After fixation, the cells were immunostained for the detection of NSP5 (StAv, green) and VP2 (anti-Flag, red). Nuclei were stained with DAPI (blue). (**b**) Immunofluorescence images of chicken LMH cells co-expressing V5-tagged NSP5 with Flag-VP2 from closely related RVB and RVG (top), and RVF and RVD (bottom). After fixation, the cells were immunostained for the detection of NSP5 (anti-V5, green) and VP2 (anti-Flag, red). Nuclei were stained with DAPI (blue). In all images, the indication at the bottom left corner corresponds to the RV species of NSP5 and VP2, respectively. The scale bar is 10 µm. The white arrows point to globular VLS. The yellow lines outline the nucleus position.

We previously demonstrated that NSP5 and NSP2 from closely related RV species pairs can be interchanged to form heterologous VLSs (76). This was observed for the pairs RVA/RVC, RVB/RVG, and RVD/RVF. Given that VP2 shares the same phylogenetic distribution with NSP5 and NSP2 (Fig. 5a), we wondered whether heterologous VLSs could also be formed by co-expressing NSP5 and VP2 from these closely related RV species. To test this, we co-expressed NSP5-BAP with Flag-VP2 of RVA and RVC in all four interspecies combinations of NSP5 and VP2 (A/A, C/C, A/C, and C/A; Fig. 8a, top panel). All the combinations supported VLS formation, although with varying morphologies, ranging from large (NSP5/RVA with VP2/RVA) to smaller, punctate structures (NSP5/RVC with VP2/RVA). In contrast, NSP5 and VP2 from RVB and RVG did not support heterologous VLS formation in any combination (B/G or G/B), either in mammalian cells (Fig. 8a, middle) or in avian cells (Fig. 8b, top). Homologous RVB VLSs were also not supported in LMH chicken cells, whereas homologous RVG VLSs were. By comparison, heterologous VLSs formed successfully in all four interspecies pairings of RVH with RVJ (Fig. 8a, bottom) and of RVD with RVF (Fig. 8b, bottom), indicating full compatibility between their respective NSP5 and VP2 proteins.

### Chimeric VP2 RVB harboring the N-terminal region of VP2 RVG forms VLS

**Fig 9:**
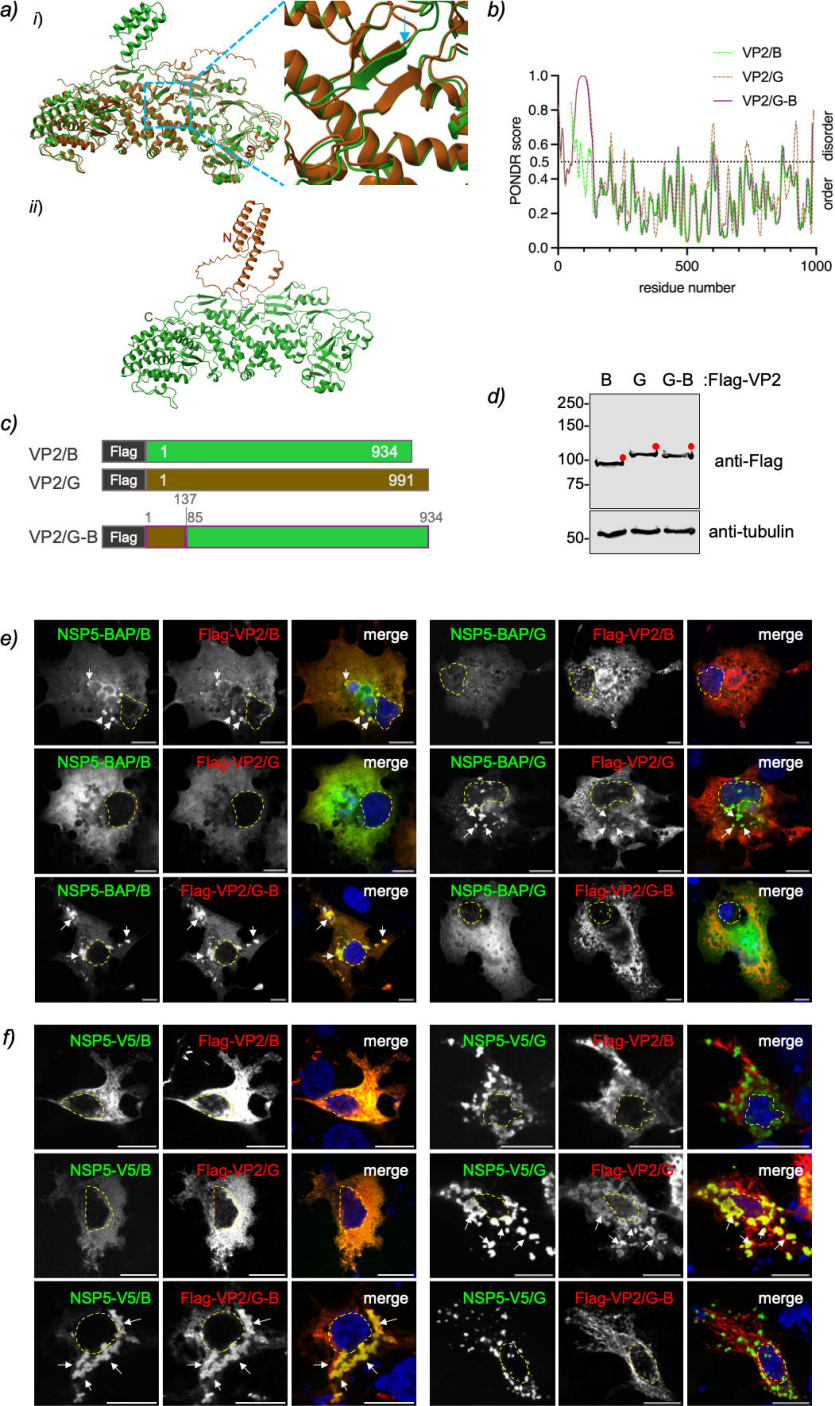
The disordered N-terminal region of VP2 is essential for VLS formation. (**a**) AlphaFold3 prediction for designing chimeric VP2/G-B. (*i*) Overlap of predicted monomer structures of VP2/B (amino acids 1–934, green) and VP2/G (amino acids 101–991, brown). The open dashed blue box highlights the magnified image on the right, showing the initial overlapping regions between VP2/B and VP2/G. The blue arrow points to V85 in VP2/B and D138 in VP2/G. (*ii*) The predicted monomeric structure of chimeric VP2/G-B, displaying the VP2/G N-terminal region (amino acids 1–137, brown) alongside VP2/B from residue 85 to 934 (green). (**b**) Plot comparing IDR predictions for VP2 species B, G, and chimeric VP2/G-B. (**c**) Schematic of Flag-VP2/B and G, along with chimeric Flag-VP2/G-B constructs. The chimeric Flag-VP2/G-B contains the N-terminal region 1–137 and the region 85–934 VP2/B. (**d**) Immunoblot of cellular lysates from MA104 cells expressing Flag-VP2/B, Flag-VP2/G, and chimeric Flag-VP2/G-B. The membrane was incubated with anti-Flag (top) and anti-tubulin (bottom) as loading controls. The red dots indicate the predicted molecular weight of each protein. Immunofluorescence images showing co-expression of NSP5/RVB (left) or NSP5/RVG (right) with Flag-VP2/B (first row), Flag-VP2/G (second row), or chimeric Flag-VP2/G-B (third row). At 16 hpt, MA/cytBirA (**e**) or LMH (**f**) cells were fixed and stained to detect NSP5 (StAv [**e**], anti-V5 [**f**], green) and VP2 (anti-Flag, red). The scale bar is 10 µm. White arrows point to globular VLS. Nuclei are outlined with a dashed yellow line as shown by DAPI staining (blue).

We previously showed that the co-expression of NSP5 and NSP2 from RVB supports the formation of globular VLSs, and NSP5 from RVB can also form heterologous VLSs with NSP2 from RVG (76). However, heterologous VLS formation between NSP5 and VP2 from RVB and RVG was not supported in both cell lines tested. Even more intriguing, homologous VLS RVB were not observed in LMH chicken cells. We hypothesized that VP2 from RVB may differ functionally from VP2 in other RV species due to the absence of an unstructured N-terminal region (Fig. 1c). To test whether this region is required for VLS formation, we used AlphaFold3 to compare the predicted tertiary structures of VP2 from RVB and its close relative, RVG. The first common structural element identified was a beta-sheet beginning at valine 85 in RVB VP2 and aspartic acid 138 in RVG VP2. Based on this, we designed a chimeric VP2 protein (VP2/G-B) by replacing the N-terminal region of RVB VP2 with amino acids 1–137 from RVG VP2 (Fig. 9a). The resulting chimera VP2/G-B was predicted using PONDR score to contain a disordered N-terminal region resembling that of VP2/G, while retaining the apical, central, and dimerization regions of RVB VP2 (Fig. 9b). We then expressed the chimeric protein as Flag-VP2/G-B, which migrated at the expected molecular weight (Fig. 9c and d).

We next co-expressed BAP- or V5-tagged NSP5 from RVB (left panels) or RVG (right panels) with Flag-tagged VP2 from RVB, RVG, or the chimeric Flag–VP2/G-B in mammalian (Fig. 9e) and avian (Fig. 9f) cells. As expected, homologous RVG VLSs formed in both cell types, whereas homologous RVB VLSs formed only in mammalian cells. Notably, co-expression of NSP5 from RVB with VP2/G-B supported VLS formation in both mammalian and avian cells, while NSP5 from RVG with VP2/G-B did not.

## DISCUSSION

Understanding the RV life cycle, particularly the assembly of virions, is crucial for controlling its spread. RV includes nine species, from A to J, that infect many mammals and birds. Notably, two new RV species (RVK and RVL) were recently added by the ICTV, although they were not part of this study. RVA viroplasms are cytosolic globular inclusions that facilitate virus genome replication, sorting, and packaging in newly assembled viral cores. Studying the life cycle of non-RVA species is difficult due to limited research tools, such as adapted viruses for tissue culture, specific antibodies, and reverse genetics tailored for non-RVA species. We recently addressed this challenge by applying the role of orthologous proteins responsible for VLS formation to non-RVA (76). Using this approach, we described how NSP5 can form VLS when co-expressed with NSP2 in certain RV species, including RVB, RVD, RVF, RVG, and RVI. Similarly, in this study, we examined VLS formation across RV species A–J by co-expressing NSP5 with VP2, using confocal immunofluorescence microscopy. We found that VLS can form across RV species A–J. These findings differ from our previous research, particularly regarding RVH and RVJ. Conversely, in other species such as RVH and RVJ, where NSP2 is not necessary, VP2 plays an essential role. Similar to RVA, NSP2 and VP2 had a complementary role in VLS formation for RVB, RVC, RVD, RVF, RVG, and RVI. Our results also show that NSP5 and VP2 directly interact, as confirmed by the BRET assay, suggesting their association influences VLS formation. These findings highlight the significant role of VP2 in viroplasm formation.

Similar to how VLSs form with NSP2 (30, 72, 76), we also demonstrate that the tail region of NSP5 is essential for both its interaction with VP2 and the induction of VLSs across multiple RV species, including RVA, RVB, RVC, RVG, RVH, RVI, and RVJ (Table 2). We previously described that the deletion of the ordered region of NSP5 in RVD and RVF, located at their N-terminus instead of the C-terminus as in other studied RV species, does not affect VLS formation induced by NSP2 (76). Similarly, the co-expression of ∆TNSP5 with VP2 of RVD or RVF enhances VLS formation in mammalian cells while consistently forming VLS in chicken epithelial cells. Notably, the VLS of ∆TNSP5 with VP2 of RVF also formed nuclear globular inclusions in both cell types, seemingly composed solely of NSP5. Therefore, the nuclear translocation of ∆TNSP5/F influences its cytosolic interaction with VP2, which is consistent with the decreased binding of these two proteins in the BRET assay. We want to point out that the addition of a Flag tag at the N-terminus of VP2 was based on previous evidence showing that the N-terminus of VP2 is flexible, and HA tagging the N-terminus of VP2 RVA supports the formation of VLS (58, 71, 79).

The viroplasms are complex structures composed of several viral proteins, each potentially contributing to viroplasm morphology. We previously demonstrated that RVA VLS induced by either NSP2 or VP2 can recruit other viral proteins (39, 46, 58, 77). Here, we determined that VLS induced by VP2 could incorporate NSP2 for RV species A–J. Interestingly, when NSP2, NSP5, and VP2 from various RV species were expressed, most formed VLSs with a globular shape. However, RVC was an exception, producing filamentous VLSs, similar to those induced by RVC NSP5 and NSP2. This result suggests that NSP2 plays a major role, over other RVC proteins, in determining the morphology of RVC VLSs. In contrast, VLS induced by VP2 from RVH and RVJ permitted the recruitment of NSP2, maintaining their globular morphology (Table 2). We previously demonstrated that the association of the respective NSP5 and NSP2 of RVH and RVJ is weak or not detectable (76). Here, we show that NSP5 and VP2 from these RV species interact and form VLSs, suggesting that VLSs composed of NSP5, NSP2, and VP2 arise either through direct interaction of both NSP5 and NSP2 with VP2, or that VP2 enhances the otherwise weak association between NSP5 and NSP2.

Our earlier findings showed that a conserved residue in VP2 RVA, L124, is necessary for the formation of VLS as well as for maintaining globular morphology and the ability of viroplasms to replicate (58). Here, we found that this conserved residue occupies a similar tertiary position in RV species B through J, as a leucine for RVC, RVD, and RVF, and as an aromatic residue, tyrosine, for RVB, RVG, and RVH, and phenylalanine for RVI and RVJ. Indeed, substituting this conserved residue with alanine disrupts VLS formation in RV species B–J, whether the VLS are induced by VP2 or formed by a combination of NSP5, NSP2, and VP2. The resulting disrupted VLSs showed two distinct patterns: in RVA, RVC, and RVJ, the proteins were completely dispersed throughout the cytosol, while in RVB, RVD, RVF, RVG, RVH, and RVI, the VLSs were smaller and mainly composed of NSP5 and NSP2, with VP2 dispersed in the cytosol. It is important to note that the substitution of this residue by non-bulky alanine in VP2 of all RV species tested does not seem to affect its folding, as denoted by AlphaFold 3 structural prediction and the fragment pattern from cleavage with proteinase K when compared with their respective wt VP2. These observations suggest that this conserved residue plays a critical structural role in VLS formation across RV species A–J.

Heterologous VLS formation is also observed with NSP5 and VP2 from closely related RV species, such as RVA with RVC, RVF with RVD, and RVH with RVJ, suggesting that genetic reassortment among these RV species may be possible in principle. In this sense, the NSP5 and NSP2 of closely related RV species, RVA with RVC and RVD with RVF, can also be interchanged (76). Since RVH and RVJ do not form VLS with NSP5 and NSP2, it remains unclear whether they can be interchangeable. However, we now demonstrate that NSP5 and VP2 of RVH and RVJ can form interspecies VLS. The formation of triple VLS involving NSP5, NSP2, and VP2 among RVH and RVJ suggests that reassortment may also occur. An interesting case involves RVB and RVG, which previously showed the ability to form heterologous VLS between NSP5 and NSP2. In contrast, NSP5 and VP2 behaved differently. Formation of homologous RVB VLS was supported only in mammalian cells, not in avian cells. Our results show that the lack of a disordered N-terminal region in VP2/B prevents heterologous VLS formation in chicken cells, whereas replacing this region with that of VP2/G enables VLS formation with NSP5/B but not with NSP5/G. These findings suggest that reassortment in these RV species depends not only on viral proteins but also on host proteins provided by specific cellular environments. Nonetheless, we cannot rule out the possibility that a natural recombination of VP2 of RVB with its closely related VP2 RVG could lead to the acquisition of a disordered N-terminal region. Notably, the VLS (NSP5 + VP2) formation is supported between intraspecies strains, as previously demonstrated with VP2 RVA simian strain SA11 with NSP5 RVA from either simian strain SA11 or porcine strain OSU (58). Similarly, VLSs are also supported with NSP5 RVA simian strain SA11 with VP2 RVA from either simian strain SA11 or porcine strain OSU (Fig. S7a). In this context, intraspecies reassortment supporting VLS (NSP5 + VP2) formation is plausible since the high similarity of NSP5 and VP2 between strains (Fig. S7b and c). Therefore, we also describe in this study for the first time that the disordered region of VP2 not only plays a role in the association with replication intermediates VP1 and VP3 in the core virion (22, 71, 73, 79) but also in the formation of VLS and, by extension, probably of viroplasms. However, it is important to keep in consideration that NSP5, NSP2, and VP2 are only a few elements in the RV life cycle, and their interaction with RdRp VP1 could also influence reassortment (80).

A previous study demonstrated that RVA viroplasms act as liquid-liquid phase-separated structures, driven primarily by NSP5 and NSP2, while VP2 was not examined due to difficulties in maintaining it in a homogeneous solution (45). However, it was suggested that the positively charged surface of NSP2 and poly-arginine-rich motifs in the N-terminus of RVA VP2 might facilitate droplet formation with NSP5. Consistent with this observation, non-RVA VP2 proteins are also enriched in basic residues (lysines and arginines) in their predicted N-terminal region (Table 3), ranging from 9.4% in RVB to 26.6% in RVD. Moreover, the present study provides essential insights into the ability of VP2 in non-RVA species to act as a client protein within NSP5 condensates, particularly in the formation of VLS in RVH and RVJ, which can arise only through association between NSP5 and VP2, and not with NSP2. Further research is needed to explore the liquid-liquid phase separation properties of VLS in non-RVA species in greater depth.

**TABLE 3:** Biophysical characteristics of the VP2 N-terminal regions of RV species A through J^*b*^

VP2	Predicted N-terminal region^*a*^	Lys	Arg	% Basic residues
RVA	1–131	6	25	23.7
RVB	1–85	7	1	9.4
RVC	1–90	12	4	17.8
RVD	1–124	27	6	26.6
RVF	1–138	25	4	21.0
RVG	1–137	28	2	21.9
RVH	1–130	22	2	18.5
RVI	1–128	19	3	17.2
RVJ	1–131	12	3	11.5

^*a*^ Predicted with PONDR score (VSL2). The numbers correspond to the first and last amino acid of the N-terminal region.

^*b*^ Lys and Arg present in the VP2 N-terminal region.

RV reverse genetics has been established only for certain RVA strains, such as simian SA11 (81), porcine OSU (82), and human KU (83), and is not available for other RVA strains and non-RVA species. Understanding viroplasms is crucial for applying reverse genetics to non-RVA species, as the co-expression of proteins like NSP5 and NSP2 significantly enhances the recovery of recombinant rotaviruses (84). For future experiments exploring reverse genetics in other RV species, it appears that for RVH and RVJ, the co-expression of NSP5 and VP2 will be favored, instead of NSP5 and NSP2, for the successful recovery of recombinant virus.

## MATERIALS AND METHODS

### Cells and viruses

MA104 (embryonic rhesus monkey kidney, ATCCCRL-2378, RRID: CVCL_3845) cells were cultured in Dulbecco’s modified Eagle’s medium (DMEM, Gibco BRL) supplemented with 10% fetal calf serum (FCS, AMIMED, Bioconcept, Switzerland) and penicillin (100 U/mL)-streptomycin (10 µg/mL). MA/cytBirA (39) were cultured in DMEM supplemented with 10% FCS, penicillin (100 U/mL)-streptomycin (10 µg/mL), and 5 µg/mL puromycin (InvivoGen, France). LMH cells (chicken hepatocellular carcinoma epithelial, ATCCCRL2117) were cultured in Waymouth’s MB572/1 (Sartorius) medium supplemented with 10% FCS and penicillin (100 U/mL)-streptomycin (100 µg/mL). HEK-293T (human embryonic kidney, ATCCCRL-3216) cells were cultured in DMEM supplemented with 10% FCS and penicillin (100 U/mL)-streptomycin (10 µg/mL).

The recombinant vaccinia virus encoding T_7_ RNA polymerase (strain vvT7.3) was amplified as previously described (85).

### Antibodies and reagents

Guinea pig anti-VP2 was described previously (77). Mouse monoclonal (mAb) anti-tubulin (clone B5-1-12) and mouse mAb anti-Flag (clone M2) were purchased from Merck. AlexaFluor 594 anti-HA.11 (clone 16B12) and AlexaFluor 647 anti-Flag Tag (clone L5) were purchased from BioLegend. Mouse mAb-V5 Tag-Dylight 488 was purchased from Invitrogen. Streptavidin-Dylight488 and mouse secondary antibodies conjugated to AlexaFluor 488 or AlexaFluor 594 were purchased from Thermo Fisher Scientific. The secondary antibodies for immunoblot conjugated to IRDye680CW and IRDye800CW were purchased from LI-COR. Mouse mAb anti-NanoLuc and HaloTagTMRDirectLigand (Cat# G2991) were purchased from Promega.

### Rotavirus sequences

The sequences of rotavirus NSP5 and NSP2 open reading frames from species B to J used in this study were previously published by (76). The sequences of the rotavirus VP2 open reading frames from RV species A to J are provided in the supplemental material and Table 1.

### Plasmid constructs

The plasmids pCI-NSP5-BAP/A, B, C, D, F, G, H, I, and J; pCI-BAP-NSP5/D and F, pCI-NSP5-V5/G, pCI-NSP5∆T-BAP/A, B, C, G, H, I, and J; pCI-BAP-∆TNSP5/and F; and pCI-NSP2-HA/A, B, C, D, F, G, H, I, and J were described previously (76). pCI-V5-NSP5/D, pCI-V5-NSP5 (15–195)/D, pCI-V5-NSP5/F, and pCI-V5-NSP5 (19–218)/F were obtained by PCR amplification of pCI-NSP5/D and F (76) using specific primers to insert *Mlu*I/V5 tag and *Not*I sites, followed by ligation into those sites in pCI-Neo (Promega). pCI-Flag-VP2/A was obtained by PCR amplification of pCI-HA-VP2(SA11) (58) using specific primers to insert *Mlu*I/Flag tag and *Not*I sites, followed by ligation in those sites in pCI-Neo. pCI-Flag-VP2/B, C, F, and I were obtained by PCR amplification from baculovirus encoding VP2/B, C, F, and I (generously provided by Dr. Daniel Luque, USW, Australia) using specific primers *Mlu*I/Flag tag and *Not*I sites, followed by ligation in those sites in pCI-Neo. pCI-Flag-VP2/D, G, H, and J were obtained from digestion with *Mlu*I and *Not*I of DNA synthetic segments (GenScript, Netherlands, Table S2) encoding for the respective Flag-VP2 and ligation on those sites in pCI-Neo.

The plasmids pCI-HaloTag-NSP5/A, B, C, D, F, G, H, I, and J were obtained from the digestion of *Mlu*I and *Not*I restriction enzymes of their respective pCI-NSP5(78) and cloned in pCI-HaloTag on those sites. The plasmid pCI-HaloTag was obtained by synthesis of the HaloTag fragment (GenBank: MG867371.1) flanked at 5'- and 3'-ends by *Xho*I and *Mlu*I restriction sites (GenScript, Netherlands) and ligated in those in pCI-Neo (Promega). The plasmids pCI-HaloTag-NSP5 (1–178)/A, pCI-HaloTag-NSP5 (1–124)/B, pCI-HaloTag-NSP5 (1–150)/C, pCI-HaloTag-NSP5 (15–195)/D, pCI-HaloTag-NSP5 (19–218)/F, pCI-HaloTag-NSP5 (1–114)/G, pCI-HaloTag-NSP5 (1–151)/H, pCI-HaloTag-NSP5 (1–104)/I, and pCI-HaloTag-NSP5 (1–136)/J were obtained by digestion with *BspE*I and *Mlu*I restriction enzymes from their respective pCI-NSP5∆T-BAP (76) and ligated in those sites in pCI-HaloTag-MEB-stop. The plasmid pCI-HaloTag-MEB-stop was obtained by annealing of the oligonucleotides 5'-cgcgtgaattctccggatgagc-3' and 5'-ggccgctcatccggagaattca-3', followed by ligation in pCI-HaloTag between *Mlu*I and *Not*I restriction sites. pCI-NanoLuc-Flag-VP2/A, B, C, D, F, G, H, I, and J were obtained from PCR amplification of their respective pCI-Flag-VP2 using a specific primer to insert in frame *Mlu*I and *Not*I restriction sites in Flag-VP2. The PCR fragment was subsequently ligated *Mlu*I/*Not*I sites in pCI-NanoLuc. The plasmid pCI-NanoLuc was obtained by synthesis of the NanoLuc fragment (GenBank: AHH41346.1) flanked at 5' and 3' ends by *Xho*I and *Not*I restriction sites (GenScript, Netherlands) and ligated into pCI-Neo (Promega).

The version of the constructs pCI-Flag-VP2/A, B, C, D, F, G, H, I, and J as well as pCI-NanoLuc-Flag-VP2/A, B, C, D, F, G, H, I, and J harboring VP2 point mutations L124A, Y129A, L126A, L157A, L146A, Y183A, Y179A, F180A, and F184A, respectively, was built by insertion of point mutations using the QuickChange site-directed mutagenesis protocol (Agilent).

The chimeric pCI-Flag-VP2/G-B was obtained by insertion in between *Mlu*I and *Pci*I of pCI-Flag-VP2/B of a synthetic DNA segment (GeneArt Technology, Invitrogen, Table S2) containing an in-frame sequence of Flag tag, N-terminal region of VP2/G (region 1-137) and VP2/B region 85–163.

All the oligonucleotides were obtained from Microsynth AG, Switzerland, and described in Table S3.

### AlphaFold predictions

Protein structures of VP2 dimers were predicted using the AlphaFold3 server (https://alphafoldserver.com/about) (86). As a reference for VP2 folding, the PDB of RVA VP2 strain RRV was used (6OGZ, https://doi.org/10.2210/pdb6OGZ/pdb).

### IDR predictions

The intrinsically disordered regions of proteins were determined with PONDR (Molecular Kinetics, Inc., https://www.pondr.com/) using the VSL2 algorithm. Data were plotted with GraphPad Prism (version 10.4.2).

### Immunofluorescence

MA/cytBirA and LMH cells were transfected and treated for immunofluorescence, as described previously by (76). For VLS formation composed of NSP5 and VP2, a ratio of 2:1 was used, with 2 µg and 1 µg of DNA plasmids, respectively. With the exception of VLS induced by VP2 of RVI, which were obtained with a transfection ratio of NSP5 and VP2 of 1:2, respectively (Fig. S2), VLS composed of NSP5, VP2, and NSP2 was obtained with a ratio of 2:1:1 using 2 µg, 1 µg, and 1 µg of DNA plasmids, respectively. The images were acquired using a confocal laser scanning microscope (DM550Q, Leica). Data were analyzed with Leica Application Suite (Mannheim, Germany) and ImageJ2 (version: 2.16.0/1.54 p, https://imagej.net/software/imagej2/).

### Immunoblotting

Cell lysis and immunoblotting procedures were performed as described by Lee et al. (76).

### Detection of Halo-tagged proteins

MA104 cells seeded at a density of 2 × 10^5^ cells per well in a 12-well plate. The cells were infected with vvT7.3 (multiplicity of infection [MOI]: 1 PFU/cell), followed by transfection with 1 µg DNA plasmid using 3 µL of Lipofectamine 2000 (Thermo Fisher Scientific) according to the manufacturer’s instructions. At 16 hpt, the cells were lysed in 30 µL TNN buffer (100 mM Tris-HCl, pH 8.0, 250 mM NaCl, 0.5% nonidet P-40, and cOmplete protease inhibitor cocktail [Roche, Switzerland]) for 10 min on ice. The cell lysate was centrifuged at 17,000 × *g* for 7 min at 4°C. Then, 10 µL of supernatant was incubated with 10 µL of 2.5 µM HaloTagTMRDirect Ligand (Promega) in DMSO. The sample was incubated for 30 min in the dark at room temperature, followed by the addition of 10 µL of sample buffer (8% SDS, 40% glycerol, 200 mM Tris-HCl pH 6.8, 0.8% bromophenol blue, and 5 mM 2-mercaptoethanol). The samples were heated at 70°C for 3 min and migrated in an SDS-polyacrylamide gel followed by acquisition at 520 nm channel at Odyssey M Imager (LI-COR Biosciences).

### NanoBRET protein-protein interaction

HEK-293T cells were seeded at 8 × 10^5^ cells per well in six-well plates. At 4 h post-seeding, the cells were transfected in a ratio HaloTag: NanoLuc of 10:1, by adding 2,000 ng and 200 ng of the respective DNA plasmids, using 6 µL of Lipofectamine LTX transfection reagent (ThermoFisher Scientific) diluted in 100 µL of Opti-MEM reduced medium. The transfection mixture was incubated for 30 min at room temperature and added to the cells. At 20 hpt, the cells were counted and diluted to 2 × 10^5^ cells per mL in 4% FCS in OptiMEM-I reduced serum medium. Then, 500 µL of diluted cells was mixed with 0.5 µL of 0.1 mM HaloTagNanoBRET618 Ligand (+ ligand, Promega) or 0.5 µL DMSO (-Ligand). Then, 40 µL of each mixture was distributed in quadruplicates in a white wall 384-wells plate. The cells were incubated for 6 h at 37°C and 5% CO2. Afterward, 10 µL of 5× solution of NanoBRET Nano-Glo substrate in Opti-MEM reduced serum medium was added per well. The luminescence was measured in a range of 10 min, at 460 nm and 618 nm for donor emission and acceptor emission, respectively, using a Spark instrument (TECAN). The BRET ratio corresponds to the mean corrected mBU, which is obtained as follows:


$$Mean\;corrected\;mBU=\;Mean\;mBU_{\left(+ligand\right)}-Mean\;mBU_{\left(-Ligand\right)}$$

Where: mBU=(618 nm/460 nm) ×1000.

Statistical analysis was performed using:


$$Z^{\prime }factor=1-[(3\times {STDV}_{\left(+ligand\right)}+3\times {STDV}_{(-ligand)})/(mean\;mBU_{\left(+ligand\right)}-mean\;mBu_{-ligand})].$$

### Phylogenetic tree analysis

The CDS for rotavirus VP2 proteins was translated *in silico* into amino acid sequences using EMBOSS “transeq” (http://emboss.open-bio.org). The protein sequences were aligned using “mafft” (MAFFT v7.475 [23 November 2020]; https://mafft.cbrc.jp/alignment/software/), and the aligned protein sequences were backtranslated using EMBOSS “transeq” (http://emboss.open-bio.org) (87, 88). The phylogeny from the nucleotide multiple sequence alignments was then inferred by using “BEAUTi” and “BEAST” (v1.10.4) (89). In brief, 10,000,000 Markov chain Monte Carlo steps with the Juke-Cantor model were performed, saving each 10,000th tree. After the burn-in of 100,000 states, the consensus tree for VP2 was calculated and visualized using FigTree (https://beast.community).

## Data Availability

The GenBank accession numbers for NPS5 and VP2 of RV species A–J used in this study are listed in Table 1. The sequences of the open reading frames of VP2 are detailed in the supplemental material. The GenBank accession numbers for NSP2 and the open reading frames for NSP2 and NSP5 of RV species A–J are available in Lee et al. (78).

## SUPPLEMENTAL MATERIAL

Supplemental figures jvi.00990-25-s0001.pdf Figures S1 to S7.

Supplemental material jvi.00990-25-s0002.pdf Legends for Fig. S1 to S7, sequences, Tables S1 to S3, and supplemental methods.

ASM does not own the copyrights to Supplemental Material that may be linked to, or accessed through, an article. The authors have granted ASM a non-exclusive, world-wide license to publish the Supplemental Material files. Please contact the corresponding author directly for reuse.
