# Supplementary material for: Comparative analysis of NSP5/VP2-induced viroplasm-like structures in rotavirus species A to J
Source: J Virol. 2025 Oct 14;99(11):e00990-25. doi: 10.1128/jvi.00990-25 (PMC12646009; doi:10.1128/jvi.00990-25)
Supplement: Supplemental figures — Figures S1 to S7. [file jvi.00990-25-s0001.pdf]

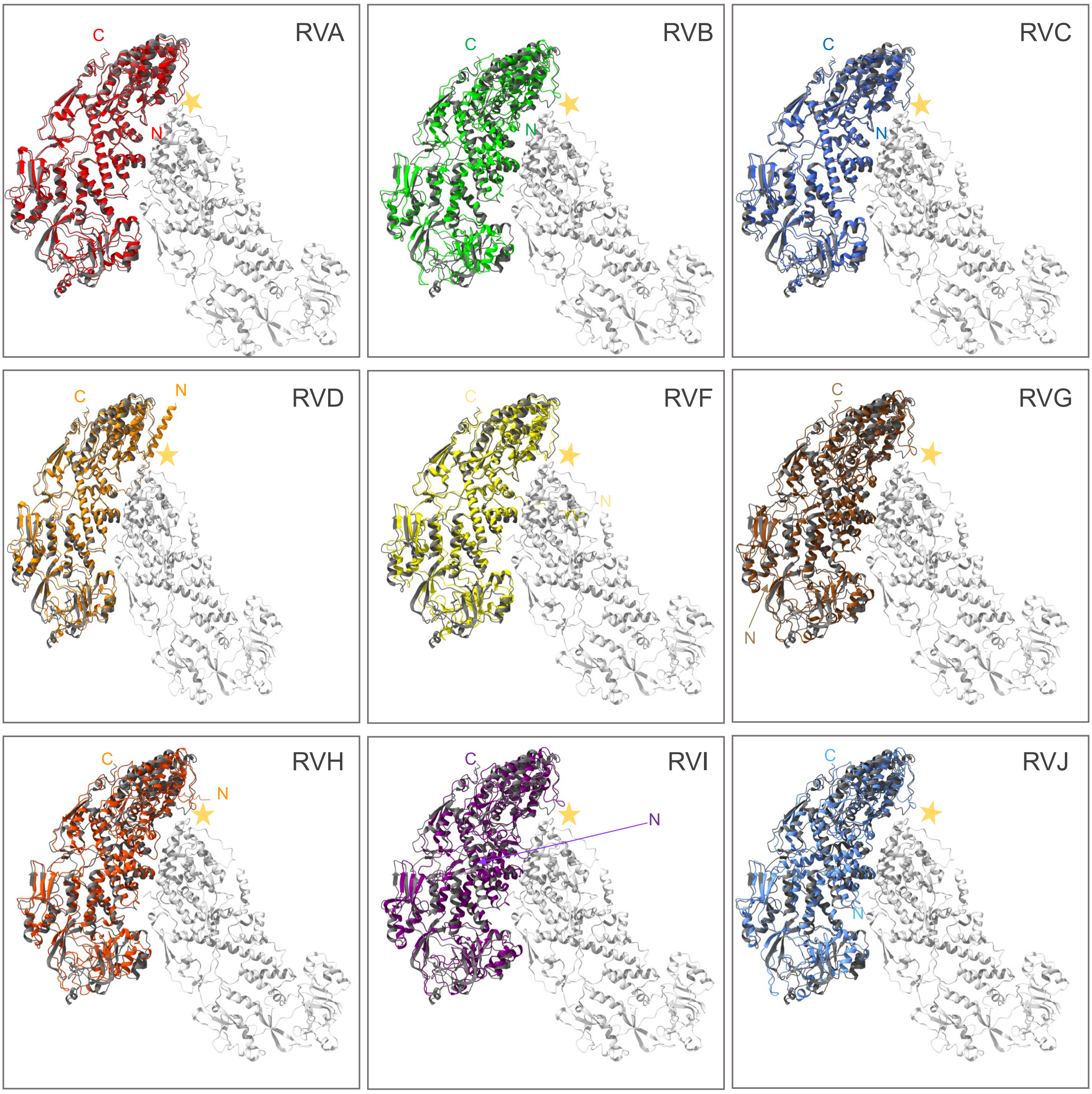
RVA
C
N
RVB
C
N
RVC
C
N
RVD
C
N
RVF
C
N
RVG
C
N
RVH
C
N
RVI
C
N
RVJ
C
N

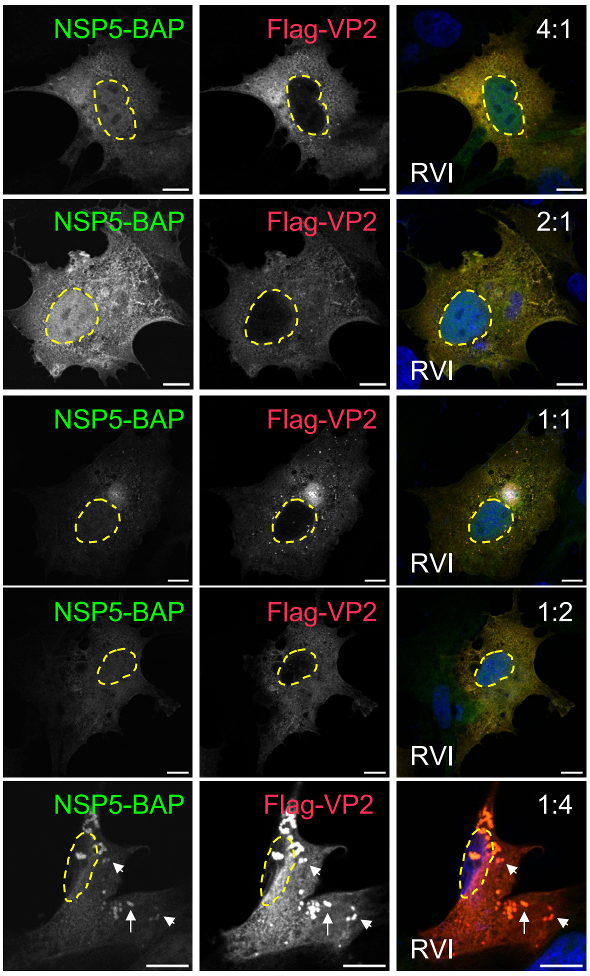
NSP5-BAP
Flag-VP2
4:1
RVI
2:1
1:1
1:2
1:4

Figure S3

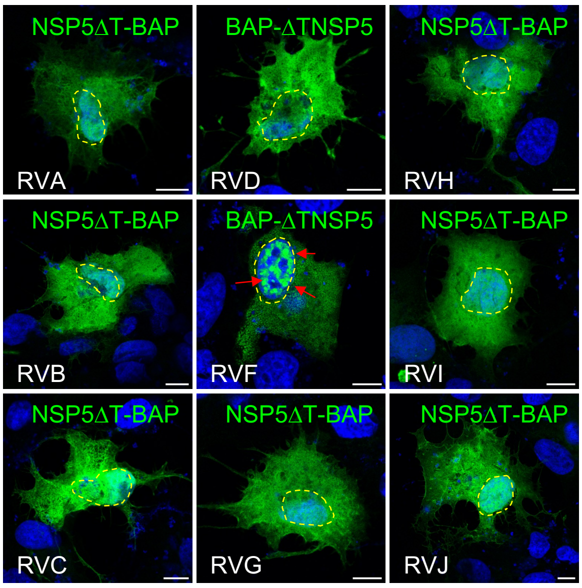
NSP5ΔT-BAP
RVA
BAP-ΔTNSP5
RVD
NSP5ΔT-BAP
RVH
NSP5ΔT-BAP
RVB
BAP-ΔTNSP5
RVF
NSP5ΔT-BAP
RVI
NSP5ΔT-BAP
RVC
NSP5ΔT-BAP
RVG
NSP5ΔT-BAP
RVJ

Figure S4

a)

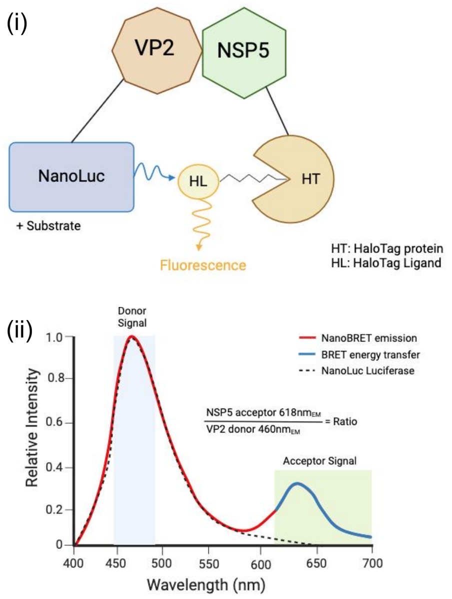

b)

HaloTag -NSP5	Predicted MW (kDa)	HaloTag -NSP5ΔT	Predicted MW(kDa)
RVA	55.7	RVA (1-178)	53.3
RVB	53.7	RVB (1-124)	48.6
RVC	57.1	RVC (1-150)	50.3
RVD	56.2	RVD (15-195)	54.8
RVF	58.3	RVF (19-218)	56.6
RVG	54.7	RVG (1-144)	50.8
RVH	54.3	RVH (1-151)	51.2
RVI	51.6	RVI (1-104)	45.9
RVJ	52.4	RVJ (1-136)	49.3

c)

NanoLuc-Flag-VP2	Predicted MW (kDa)
RVA	122.9
RVB	126.0
RVC	121.8
RVD	126.3
RVF	124.6
RVG	132.8
RVH	132.1
RVI	130.7
RVJ	132.5

d)

e)

f)

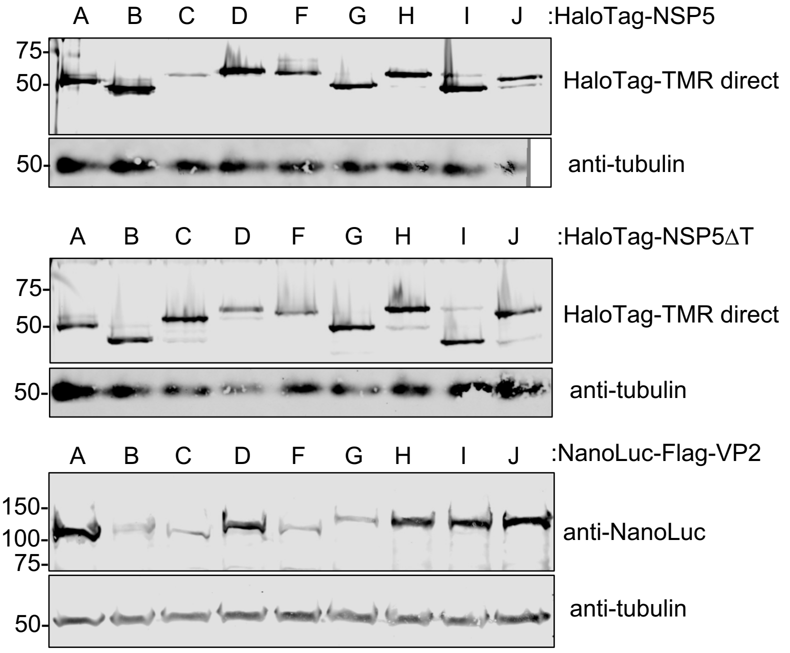

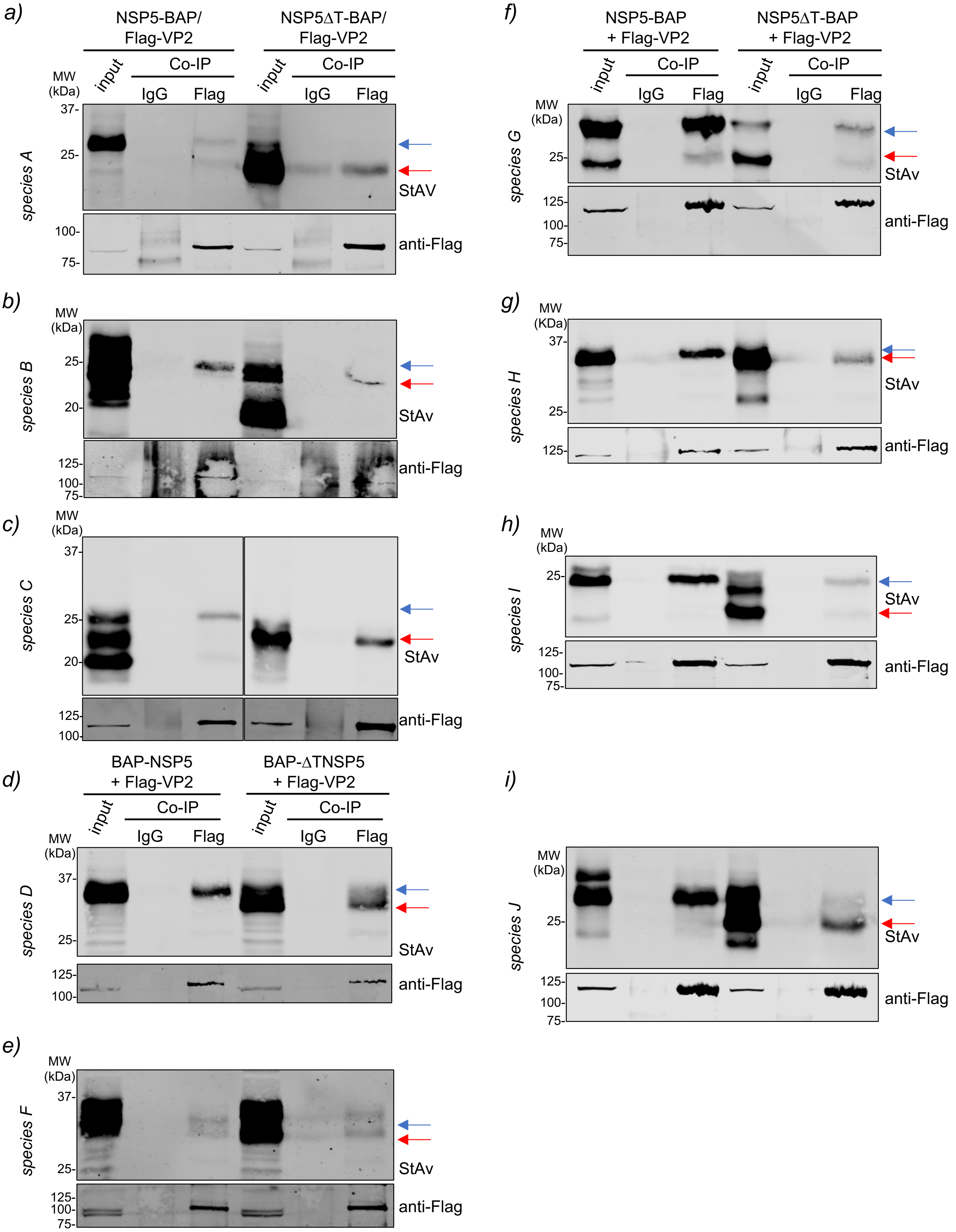
a)
NSP5-BAP/ Flag-VP2
NSP5ΔT-BAP/ Flag-VP2
input
Co-IP
IgG
Flag
MW (kDa)
37-
25-
100-
75-
species A
StAV
anti-Flag
b)
species B
25-
20-
125-
100-
75-
StAv
anti-Flag
c)
species C
37-
25-
20-
125-
100-
StAv
anti-Flag
d)
BAP-NSP5 + Flag-VP2
BAP-ΔTNSP5 + Flag-VP2
species D
37-
25-
125-
100-
StAv
anti-Flag
e)
species F
37-
25-
125-
100-
75-
StAv
anti-Flag
f)
NSP5-BAP + Flag-VP2
NSP5ΔT-BAP + Flag-VP2
species G
25-
125-
100-
75-
StAv
anti-Flag
g)
MW (KDa)
species H
37-
25-
125-
StAv
anti-Flag
h)
species I
25-
125-
100-
75-
StAv
anti-Flag
i)
species J
25-
125-
100-
75-
StAv
anti-Flag

a)

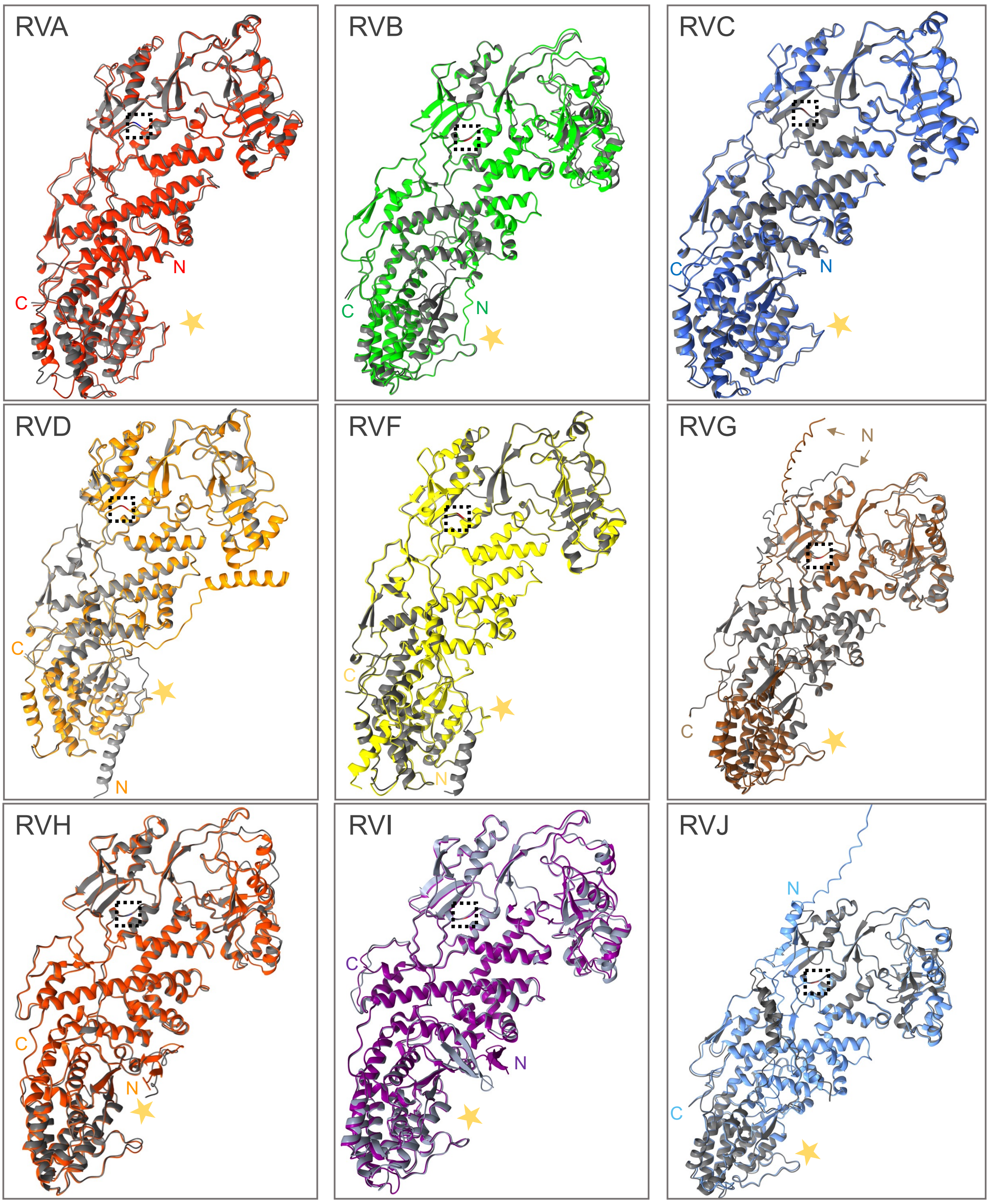
RVA
N
C
RVB
C
N
RVC
C
N
RVD
C
N
RVF
C
N
RVG
N
C
RVH
C
N
RVI
C
N
RVJ
N
C

b)

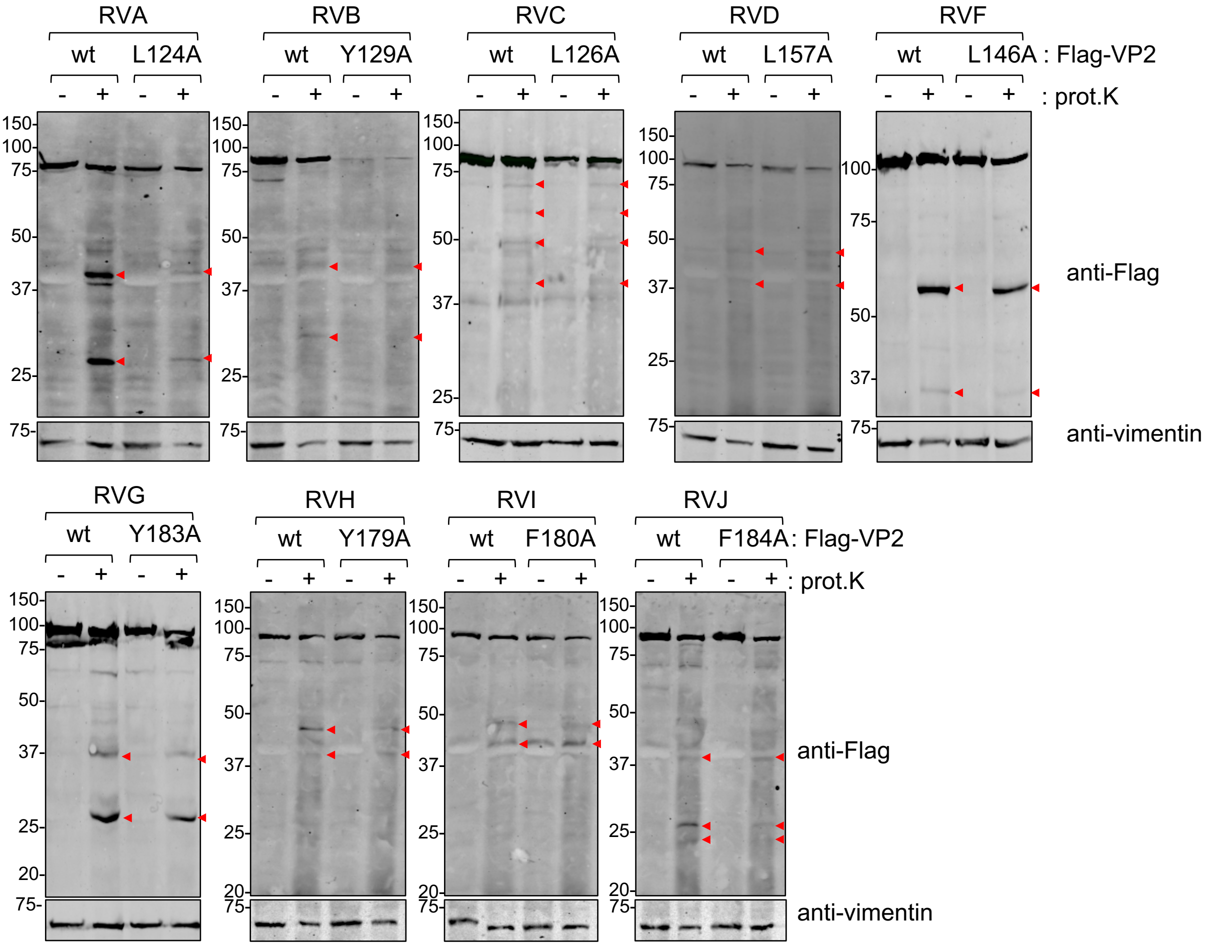
RVA
wt
L124A
RVB
wt
Y129A
RVC
wt
L126A
RVD
wt
L157A
RVF
wt
L146A
: Flag-VP2
- + - +
: prot.K
150
100
75
50
37
25
anti-Flag
anti-vimentin
RVG
wt
Y183A
RVH
wt
Y179A
RVI
wt
F180A
RVJ
wt
F184A
: Flag-VP2
: prot.K
20
anti-Flag
anti-vimentin

Figure S7

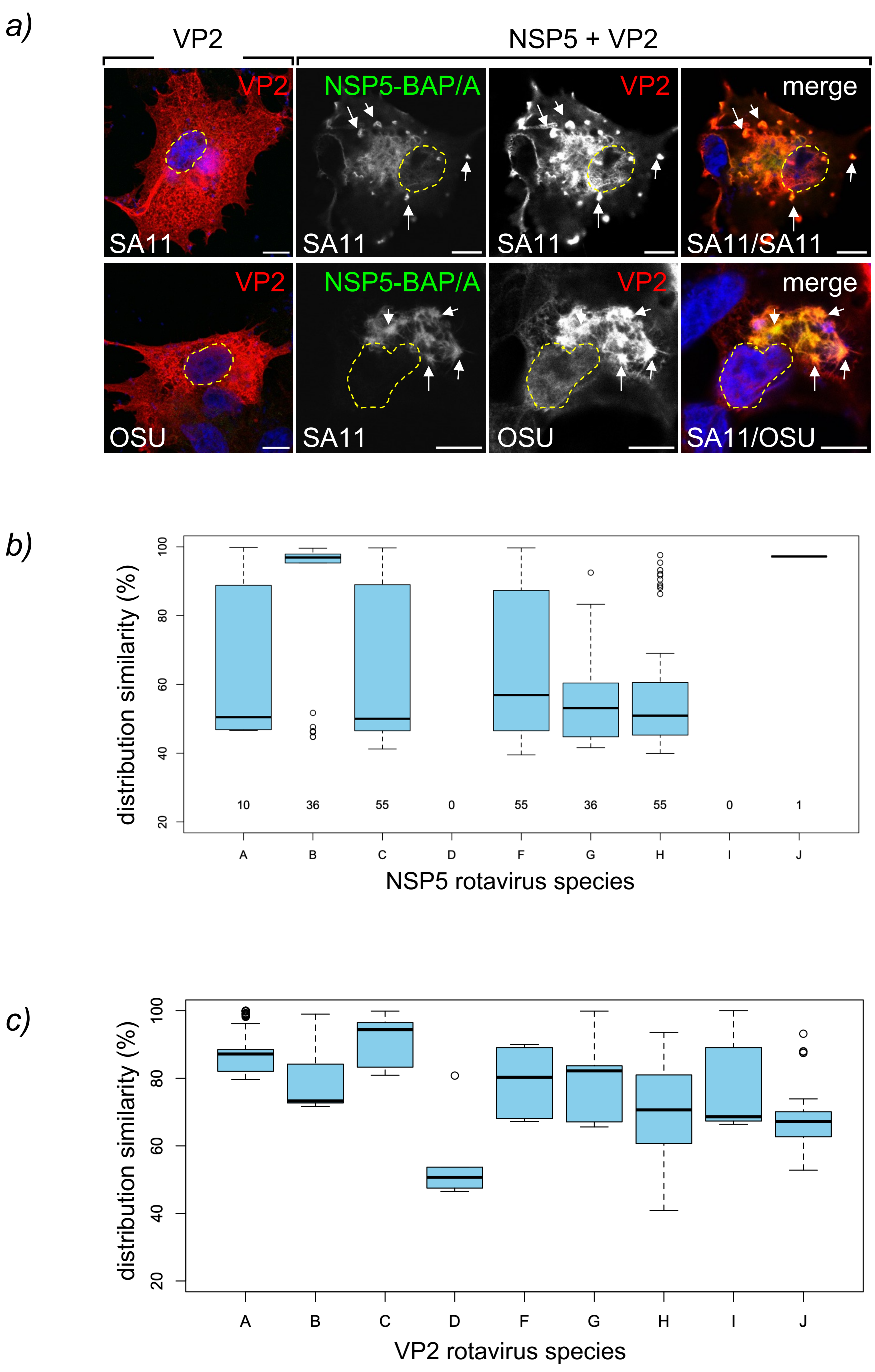
a)
VP2
NSP5 + VP2
VP2
NSP5-BAP/A
VP2
merge
SA11
SA11
SA11
SA11/SA11
VP2
NSP5-BAP/A
VP2
merge
OSU
SA11
OSU
SA11/OSU
b)
distribution similarity (%)
100
80
60
40
20
10
36
55
0
55
36
55
0
1
A
B
C
D
F
G
H
I
J
NSP5 rotavirus species
c)
distribution similarity (%)
100
80
60
40
20
A
B
C
D
F
G
H
I
J
VP2 rotavirus species
