# Supplementary material for: Comparative analysis of NSP5/VP2-induced viroplasm-like structures in rotavirus species A to J
Source: J Virol. 2025 Oct 14;99(11):e00990-25. doi: 10.1128/jvi.00990-25 (PMC12646009; doi:10.1128/jvi.00990-25)
Supplement: Supplemental material — Legends for Fig. S1 to S7, sequences, Tables S1 to S3, and supplemental methods. [file jvi.00990-25-s0002.pdf]

Supplemental Figure Legends

Figure S1. AlphaFold3 predictions for VP2. Side view of AlphaFold3 predictions of the dimeric structure of VP2 for RV species A to J. Each panel shows VP2 isoforms A (colored) and B (light grey). VP2/RVA isoform A (dark grey) from PDB(1) was overlaid with the corresponding isoform A of VP2 from RV species A to J. The star marks the position of the five-fold axes. The C-terminus is shown for each isoform A. Since the N-terminus of all VP2 had low confidence (pLDDT < 50), suggesting likely disorder, the first 100 amino acids were removed to improve and standardize the structural predictions across models. All predicted local distance difference test (pLDDT) values were above 70. The N label indicates the N-terminal after removal of the first 100 amino acids.

Figure S2. Immunofluorescence images of MA/cytBirA co-expressing NSP5-BAP with Flag-VP2 from RVI at the indicated various ratios of NSP5: VP2. The cells were fixed at 16 hpt and stained for detection of NSP5 (StAv, green) and VP2 (anti-Flag, red). The scale bar is 10 µm. A merged image is shown in the right column. The nuclei were labeled with a dashed yellow line as determined by DAPI staining (blue).

Figure S3. Expression of NSP5 with deleted tail. Immunofluorescence images of MA/cytBirA expressing tail-deleted NSP5 fused to BAP tag at N-or C-terminus region as indicated. After fixation, the cells were stained for the detection of NSP5 (StAv, green). The scale bar is 10 µm. The red arrows point to nuclear globular inclusion. The nuclei position is labeled by a dashed yellow line as determined by DAPI staining (blue).

Figure S4. Characterization of the protein-protein interaction. a) (*i*) Schematic showing energy transfer from NanoLuc-VP2 (energy donor) to a fluorescently labeled HaloTag-NSP5 (energy acceptor) upon interaction. (*ii*) Plot depicting spectral separation of NanoLuc luciferase emission (460 nm) and the fluorescent HaloTag NanoBRET ligand emission (618 nm), along with the calculation of the BRET ratio. **b)** Table with the predicted molecular weight of

HaloTag-NSP5 and HaloTag-NSP5ΔT of RV species A to J. **c)** Table with the predicted molecular weight of NanoLuc-Flag-VP2 of RV species A to J. SDS-PAGE analysis of Hek293T cells expressing HaloTag-NSP5 **(d)** or HaloTag-NSP5ΔT **(e)**. Before lysis at 16 hpt, cells were incubated with HaloTag-TMR-direct substrate for 15 min (upper panel). After image capture, proteins were transferred to a nitrocellulose membrane, then incubated with anti-tubulin as a loading control (bottom panel). **f)** Immunoblot of cellular extracts expressing NanoLuc-Flag-VP2 of RV species A to J. The membrane was incubated with anti-Flag to detect VP2 and with anti-tubulin as a loading control.

Figure S5. Co-immunoprecipitation assay for the association of NSP5 with VP2. Anti-Flag immunoprecipitated from extracts of MA/cytBirA cells co-expressing Flag-VP2 with full-length NSP5-BAP or NSP5ΔT-BAP for RV species A (a), B (b), C (c), G (f), H (g), I (h), and J (i). For RV species D (d) and F (e), the cells co-expressed Flag-VP2 with full-length BAP-NSP5 or BAP-NSP5ΔT. The membranes were incubated with streptavidin conjugated to IRDye800 (top panel) and mouse mAb anti-Flag (bottom panel). The input corresponds to 5% of crude cell extract. IgG refers to immunoprecipitation with an isotype control antibody. Blue and red arrows indicate full-length NSP5-BAP and NSP5ΔT-BAP, respectively.

Figure S6. Wild-type VP2 and VP2 harboring point mutation disrupting VLSs are structurally similar in RV species A to J. **a)** AlphaFold3 predictions of wt VP2 and VP2 harboring indicated point mutations. Each panel illustrates overlapped monomeric wt VP2 (grey) and VP2 point mutations (colored) for RV species A to J. The dashed black open box indicates the position of residue substituted to alanine [RVA (L124A), RVB (Y129A), RVC (L126A), RVD (L157A), RVF (L146A), RVG (Y183A), RVH (Y179A), RVI (F180A), and RVJ (F184A)]. The star indicates the position of the five-fold axes. The C-terminus is displayed for each isoform A. Since the N-terminus for all VP2 proteins displayed low confidence (pLDDT < 50), indicating likely disorder, the first 100 amino acids were excluded to refine and

standardize the structural predictions across models. The values of the predicted local distance difference test (pLDDT) were all > 70. The N label refers to the N-terminal region after excluding the disordered N-terminal segment, as determined by the PONDR score. **b)** Immunoblotting of Flag-VP2 cell extracts cleaved with proteinase K. MA104 cell extract expressing indicated wt or point mutations of Flag-VP2 from RV species A to J were untreated or treated with proteinase K (12.5 mU) for 2 min on ice. The membranes were incubated with mAb anti-Flag for the detection of Flag-VP2. The red arrowheads point to the cleaved fragments of wt Flag-VP2 and the corresponding point mutation with the same molecular weight. Anti-vimentin was used as a loading control.

Figure S7. Strain similarities among various RV species and their ability to form VLSs. **a)** Immunofluorescence images of MA/cytBirA cells expressing VP2 (SA11) (top panel) or VP2 (OSU) alone (first column) or with NSP5-BAP/A (SA11) (second, third, and fourth columns). At 16 hours post-transfection, the cells were fixed and stained to detect NSP5-BAP (StAV, green) and VP2 (polyclonal anti-VP2, red). Nuclei were stained with DAPI (blue). The scale bar is 10 µm. White arrowheads indicate VLSs, and the dashed yellow line outlines the nucleus based on DAPI staining. Boxplot displaying the percentage similarity of RV strains within each RV species for NSP5 **(b)** and VP2 **(c)**. The analysis included 133 sequences (30 for RVA, 17 for RVB, 31 for RVC, 4 for RVD, 5 for RVF, 11 for RVG, 27 for RVH, 7 for RVI, and 8 for RVJ), representing 17556 pairwise alignments.

Open-reading frame sequences of VP2 of RV species A to J used in this study.

Start and stop codons are highlighted in bold.

VP2-RVA

>LC178565.1 Rotavirus A VP2 gene for VP2, complete cds, strain: SA11

ATGGCGTATCGAAAACGTGGAGCGCGTCGTGAGACGAATCTAAAACAAGATGAACGAATGCAAGAAAAAGAAGATAGCAAGAA

CATTAATAATGACAGTCCTAAATCACAATTATCAGAAAAAGTATTATCTAAGAAAGAAGAGATAATTACAGATAATCAAGAAG

AAGTTAAGATATCTGATGAGGTAAAAAAATCTAATAAAGAAGAATCGAAACAGTTGTTAGAAGTACTTAAAACAAAAGAGGAA
CATCAAAAAGAAGTTCAGTATGAAATATTACAAAAAACTATCCCTACATTTGAACCAAAAGAGTCAATACTCAAAAAATTAGA
AGACATAAAACCAGAACAAGCAAAGAAACAAACTAAACTGTTTCGAATATTTGAACCGAAACAATTGCCTATTTATAGAGCTA
ATGGAGAAAGAGAGCTTCGTAATAGATGGTATTGGAAATTGAAACGAGATACTCTTCCTGATGGAGATTATGATGTTAGAGAG
TATTTTTTAAATTTATATGATCAAGTATTAATGGAAATGCCGGATTATCTATTACTTAAAGATATGGCTGTAGAGAATAAAAA
TTCAAGGGATGCTGGCAAAGTAGTTGATTCTGAAACAGCCGCAATATGCGATGCTATTTTTCAAGATGAAGAAACCGAAGGTG
CAGTAAGAAGATTCATAGCTGAGATGAGACAACGAGTTCAAGCTGATCGAAATGTAGTCAATTATCCATCTATATTGCATCCA
ATTGACCATGCGTTTAACGAATACTTCTTACAACATCAGTTGGTAGAACCATTAAATAATGATATCATTTTCAATTACATACC
AGAGAGAATAAGAAATGATGTCAACTATATATTAAATATGGACAGGAATTTACCGTCTACTGCTAGATATATCAGACCAAACT
TGCTACAAGATAGGTTAAATTTACATGATAATTTTGAGTCACTCTGGGATACTATAACTACATCTAATTATATTTTAGCAAGA
TCTGTGGTGCCAGACCTAAAAGAATTAGTATCTACTGAGGCACAAATCCAGAAAATGTCACAAGATTTGCAATTGGAAGCTTT
GACAATACAATCAGAGACTCAGTTTTTAACAGGTATAAACTCACAAGCCGCTAATGATTGTTTTAAAACTTTGATTGCTGCTA
TGTTGAGTCAGAGAACCATGTCATTAGATTTCGTAACGACAAATTACATGTCACTTATTTCAGGCATGTGGTTACTCACTGTG
ATTCCAAATGATATGTTTATAAGAGAATCATTAGTAGCATGTCAACTAGCCATAATAAATACCATTGTTTATCCGGCATTCGG
AATGCAAAGAATGCATTATAGGAATGGTGATCCACAGACTCCCTTTCAAATTGCAGAGCAACAGATTCAAAATTTTCAGGTAG
CTAATTGGTTACATTTTGTTAATTATAATCAGTTTAGACAAGTAGTGATTGATGGAGTGTTAAATCAAGTCTTGAATGATAAT
ATAAGAAATGGTCATGTAGTCAACCAATTAATGGAAGCTCTGATGCAATTATCTAGACAACAGTTTCCCACAATGCCAGTTGA
TTATAAAAGATCTATACAGAGAGGAATTTTGCTGCTTTCTAACAGACTTGGTCAGCTTGTCGATTTAACAAGATTGTTATCAT
ACAATTATGAGACATTAATGGCATGCATAACAATGAATATGCAGCATGTTCAAACATTAACAACTGAAAAATTGCAATTAACA
TCAGTAACATCATTATGTATGCTAATTGGAAATGCTACGGTTATACCGAGTCCGCAAACATTGTTCCATTACTATAATGTGAA
TGTCAATTTTCATTCAAATTATAATGAAAGAATTAATGACGCAGTTGCAATTATAACTGCGGCAAATAGATTAAATTTATATC
AAAAGAAAATGAAATCAATAGTTGAGGACTTTCTGAAAAGATTACAGATATTTGATGTTGCGAGAGTACCAGATGACCAAATG
TATAGATTGAGAGATAGATTAAGACTATTACCAGTTGAAATAAGAAGATTAGATATTTTTAATTTGATAGCAATGAATATGGA
ACAGATTGAACGTGCATCAGATAAAATTGCACAAGGAGTTATAATAGCATACCGAGATATGCAGTTAGAACGAGATGAGATGT
ATGGTTACGTCAATATTGCCAGAAACTTGGACGGATTTCAACAAATAAATCTTGAAGAATTGATGAGATCAGGAGATTATGCT
CAAATTACTAACATGCTACTTAATAATCAACCAGTAGCTTTAGTTGGAGCGCTACCATTTATAACGGATTCATCAGTGATTTC
GTTAATAGCTAAACTAGATGCAACCGTTTTTGCACAGATTGTCAAACTTAGAAAGGTCGACACGTTAAAACCCATCCTATATA
AGATAAATTCAGATTCTAATGACTTTTATTTGGTGGCTAATTATGATTGGATTCCTACATCTACTACAAAAGTGTATAAACAA
GTTCCACAACAATTTGATTTTAGAGCGTCAATGCATATGTTAACGTCTAACCTAACATTTACCGTATATTCAGATTTGCTTGC
GTTCGTTTCAGCTGATACTGTTGAACCAATTAATGCTGTTGCTTTTGATAATATGCGCATCATGAACGAACTG**TAA**

VP2-RVB

>NC_021545.1 Human rotavirus B strain Bang373 inner capsid protein (VP2) gene, complete cds

ATGGATTCAACTGCCTTAGTCGAATCTGCTAAAACCAACATTCATGGAGTTGACTCAAAAACGGAGAAGCAAACTATATTTGA
TCAACTGATATCAGATATAAAGAGTCAGAATGATGGCCAAATTCCGGATGAAGTACTTCCAGATTTACTACAACTGGCTGAAA
TTAATGGACTAACGTTTGAGTACAAACCAAAGGAAAAGTTAAGTATCATGGATCGTCCAGATCCAACGTCTGTACTTTCACAG
GACGTCTTCCAAATTAGAACAATTTTATCGAAAACGCTATTCGTTGATGTCGAAAATGAAGATTATTCAGTATATATCCCTAA
TGAAACGACGAAATTGACTCCTGTGTCAATTGATGCAAGACCAATCCAGACTTACCATCCTAAAGCTTTGATGTACAAAGACA
CTGCGATTCTTCCCTCTCATAGAGATGAGATATCAGATCAATATGGTACTGATGAGATATTATTTGATTCTCACATGTTCAAT
GATATCAGTCAAGCTCAAATACGAGATTTTGACACGTACATATTAGATAAATCAATTCAAATTCAAAACACGCTACCAAATTT
AGAGTTTATTTCCGCACTGGAAAAAGAAGTGAACCCTTTCAATATACACAACACACTATGTCTTAATTTCGGTCAAAAAGAGT
ATTACAACATAATTGCAGATCGTACGAATCTTTCTTTCCAACAGAGGAGACATTCAGTTCAATTTGACAATGTTGTCGTTGAC
GGAGTGGCTAGAACTGCCAGGGTTTCACTACGACTTCATCCATTTGACAGCCAACTGTTAGACATAGTAAGATTTAACGTTAT
CCAAGACCAGCCCTTGGCTGACATTTTAATGGAATATCAATTAGTGGCAGCAGATGGATTTGTCGCCACTCCAAAATTTCGTG
TTGACCGTGACGCCAGACTAATCGCAGATGTAAGATCTCCAGTCATGGCTAGATTGTGCGAATTGTCGCCATATTTTCATAGA
ACACGAATATTGTCTTCAATGACCGATTTTACACCACTATGGAAAGTCAATGTTTTCTCAAGTTCTATAGATAACGCTAAAGA
TGCAATTTATCGCATGGCTGAAATTTCTTTTACCGTCGCAGACGCCACAACGTCTGCTCTTGCGTCTGTGAATGTAGCCTCCG
CTCAGCAGACTCTTTTGACATTACTGAATTTGTCCCTTTTTCGTTTCGAAATTGATCCGACCGGAAGCCAATCAAATTTCGGC
TCCGCTGTCTCTGCAGCTCTAATGCTGATCGTATTTCCAACTGATGAGCAATCAATGTCTAATATCACTTTTGACAATTTGTG
TAATTTGGTATTCAATGAGTTGATTGCTTGGACCGTTGATCGGCCGACATTCGTCAAGAGAACTGGTATGACAAACGCTTTCG
AAGCGAATGTGAATATTGGTGGTGGTAATATGACTAGAGACATAATTGCGTACATGAGGTTTGTGTTACTACGCAGACCATGG
GCTGTTTTTCAACGGGCGTATGATGATAGATACGTGTCTGACATTATGGTGCCAAATATCGATGAAGCCAACGTCAATGATCA
ATGCTACGTCGCCATTAACAACCTGTTTAATGGATTGATACAAGCAGCACAAAGAAATCCCAACCCAGGACGGCAGATAGCTG
CCACTTCTTTTAGGAAATTGCTGAAATCTATGAAGGATTCATGTTGTAATAGGATGATACCATTAATCAGATTATTAAAGTAT
AACATTGAGAGAATCGCCCGTGTATACAGGTTTTTTCCATATACAGCTGATTTAGTACACATCATTCCTGCTTTTAGAGATGA
GAGACTAAGAGTGAAAGTTCCAGTGTCTGGAATGTTGTCTATAGCTCTCGGAATTAACAAAGCTCCAGATTCCTTCGATTGGT
ACAATTTGCTTAAATTTGCTGATGTTGTAAGAACTAAGAACTTCGCGGAACGACAATCTCTAGAATCAATTATGGTGCACGCA
CTGATCAGAAATGACATAAATCCCGCGCGATCAAAAAAAGACTATATACAGCAAAATATCAAACCAGCAACGAACGTTGTCGC
ATCACTGTCTAAATTGCCATCAGCAACATTCACAACAATTCTCGCGGATCGGATGCTTAATAATGAGATAAGGCGAACTCAGA
GCTATGTGGTTACAAATCGTATTCGTGATGCTGTCAGAGCTGCATTTGAACACGTGCCAACAGCTGAACATGGGATCGCCAAG
GGTGCTCTTTTATTACCATATCCGCAAAATTTTCAACGATCCTCAGTGTATGTTAGGAAAGATAACATATTGTACGATCCTCC
AGTTGGAGTTGATAGATTCAATCTAAGTGACCTACTGGATGGTAGATTCTATCAAGGATTGATTAACCGAGTGCAAAACATGG
CGCCGTTTGTTATATCGGGACCATTGCAAGTAAAGCCATCCGATGCATCTGCGATTGAATCTGTTACATCTGCCTACCTTACA
ATGTCCTCTCCTTACGACGCTTGCGTGAGACCAGAGGATTTACGACATAACCGAGTTGTCCACCCACCTACCGTTGATTATTT
TAGTGATGCTAGTATTGCTAGACCTAATACACAATTTGAGCAGCTGATGTCAAAAACGTCAGTATTTGTTATTGACGCTCCAA
GACTTATAGTTCAAAATGACGCTACTGTATACACATTCGACTACAAAGACATTCAACTCACAACATCCGTCGTAGATAAATTA
GAATTTACCTCAGTTAAAACGCCAGACGTTACTCTATTTAATGGAATGTTGGTTTATGAAGAT**TAA**

VP2-RVC

>NC_007546.1 Rotavirus C segment 2, complete sequence/bristol (2736 bp)

ATGATAAGCAGAAACAGGCGCAGAAATAATCAACAAAAAAACATAGAAAAAGAGAAACAATTAGAGACTATAATTAACAAAGA
AGTTAAGGAAAACAAAGATTCTATGAAAGAAGATAAGCTAGTAGTTACAGAAGAAAGCAATGGAGACGTCACAACTGCTAAAG
AACAATCGAATAATATTAATTTACAAAAGAATGATTTGGTTAAAGAAGTCATGAATATACAGAATCAAACATTAAATACAGTA
GTTACTGAGAATAAAGTTGAAATAGAAGAAATAGTTAAAAAATACATTCCATCATATAATACTGATAGCCTCATTGTTAAAAA
GTTAACTGAAATCCAGGAATCAAGTGCTAAAACATATAATACATTGTTTAGATTATTTACTCCAGTTAAAAGTTATTTATATG
ATATAAATGGTGAGAAAAAATTATCGACTAGATGGTATTGGAAATTGCTCAAAGATGATTTACCTGCTGGTGATTACTCAGTT
AGACAATTCTTCCTGTCACTATATTTAAATGTTTTAGAGGAAATGCCCGATTACATAATGCTTCGTGATATGGCAGTGGATAA
CCCATATTCAGCAGAAGCAGGTAAAATCGTAGATGGAAAGTCTAAAGAAATTTTGATTGAACTATATCAAGACCAGATGACAG
AAGGATATATTAGAAGATATATGTCTGAATTAAGACATAAAATATCTGGAGAGACAAATACTGCAAAATACCCAGCTATTCTA
CATCCCGTGGATAATGAACTTAATCAATACTTTCTTGAGCATCAGTTAATTCAACCATTAACTACAAGGAACATTGCAGAATT
GATTCCAACTCAATTATATCATGATCCAAATTACGTTTTTAATATTGATGCAGCCTTTTTAACAAATTCAAGATTTGTTCCAC
CATACTTAACACAGGATAGGATTGGATTACATGATGGATTTGAATCAATATGGGATTCAAAAACTCATGCTGATTACGTTTCA
GCTAGAAGATTTATACCTGATTTAACTGAACTGGTGGATGCTGAAAAGCAAATAAAAGAAATGGCTGCACATTTACAACTAGA
GGCTATTACGGTACAGGTTGAATCACAATTTTTAGCAGGAATTAGTGCTGCTGCAGCTAATGAAGCGTTTAAATTTATAATTG
GCTCAGTTTTATCTACCAGAACAATAGCTGTAGAATTCATAACCTCAAACTATATGTCACTAGCATCATGTATGTATTTAATG
ACTATTATGCCATCAGAGATTTTCTTAAGAGAATCATTAGTTGCTATGCAATTAGCAATAATAAATACCCTTATTTATCCAGC
TCTAGGTTTAGCGCAAATGCATTATCAAGCAGGTGAAGTGAGGACTCCATTCGAATTAGCTGAAATGCAAGTAGCTAATAGAT
CTATTAGACAATGGTTACATCATTGTAATACACTTCAATTTGGTAGACAGATAACGGAAGGGATAATTCATCTACGATTTACT
AATGATATCATGACAGGCAGGATAGTGAACTTATTTTCAACAATGTTAGTGGCTCTATCATCTCAGCCTTTCGCTACATATCC
TTTAGACTATAAAAGATCTGTACAAAGAGCGTTACAACTTTTATCAAATAGAACAGCTCAAATAGCAGATTTAACCAGATTAA
TAGTATACAATTATACTACATTATCTGCTTGTATAGTTATGAATATGCATTTAGTAGGAACTCTTACTGTTGAACGTATACAA
GCCACTTCTCTAACTTCTTTAATGATGTTAATCTCTAATAAGACAGTTATTCCGGAACCATCGTCTCTTTTTTCATATTTCTC
TAGTAACATTAATTTTCTTACAAATTATAATGAGCAAATTGATAATGTGGTAGCAGAAATAATGGCCGCATATAGATTGAATT
TATATCAACAGAAAATGTTGATGCTCGTTACCAGATTTGTGTCAAAGTTATACATATTTGATGCTCCTAAGATACCACCAGAT
CAGATGTATAGATTAAGAAACCGATTAAGAAATATTCCAGTTGAAAGAAGAAGAGCTGACGTATTCAGAATTATTATGAATAA
TAGAGATTTAATCGAAAAAACATCAGAACGTATATGCCAGGGTGTGCTGTTATCTTATACACCAATGCCTTTAACTTACGTTA
AGATGTCGGGTTAACAAATGTAATTAATGACACTAATAGCTTTCAAATAATTAATATTGAAGAAATTGAGAAGACCGGTGACT
ATTCAGCTATAACGAATGCATTACTTCGGGATACTCCAATCATATTGAAAGGTGCGATTCCATATGTTACTAACTCATCAGTA
ATTGATGTTTTATCTAAAGTGGACACCACAGTGTTCGCAAGCATTGTAAAAGATAGGGACATTTCAAAGTTAAAACCAATAAA
ATTCATAATTAATTCAGATTCATCCGAATATTATTTAGTACATAATAATAAATGGACACCAACAACAACTACAGCAGTATATA
AAGCTAGATCTCAGCAATTTGATATACAACATTCAGTATCAATGCTAGAGTCAAACTTATTTTTTGTGGTATATAATGATTTA
TTTAAATACATTAAAACCACTACAGTTCTGCCGATAAATGCTGTCTCTTATGACGGTGCAAGAATTATGCAAGAAACA**TAA**

VP2-RVD

>VP2_D_05V0049 (2801 bp)

ATGCGAAAGAATAAACTAATACAAGATAAAGAGAGAGAAAAATCTAAATCTAAAGATGAAACAAGAACCGAGCGAGGAAAGAC
TGATAGAAACACTACAAATTCGTTAAAAAATGGAGTAGAGACGGATTGTAATGATGGTAACTGCGTGGACAATGTTAATGTAG
ATAGTAAAAATGATAAAAAAGAAGAGAGAACGAAAAAAGATTACATTAAAAGTGAAAAAAAGAGTGACGGTGCATCTGATACT
AAAGAGAAAGAAAATGGATCTAAGGATAAAGTTGTATCAAATGAAGAGAATAAGAAAAATGAAAAGGAGCTAGCGGAAATTCT
GAAAACAAAAGAAGAAGTTACTAAAGACTTAGATAAAGAAATATTTTATAAATATATACCGACATTTGATGTTAATATAGATA
TTGTTAAGAAAATGTTAGATATACCATCTGTTTCGCCAAAGGATGAGAAAACATTATTTAGATTATTTGATTTAAAAAACCTG
CCATTGTATGATACTTCAGCAGTTAGGACACTAGAAACGAGATGGGTGTGGAAGCTTAAAAAAGATGATTTGCCAGATGATTC
TTACTCAGTGCGTGAGTACTTTCAAGGTTTATATGAACACATTCTATCAATAATACCAGATTATATAATGTTGAGGAATATGG
CTGTGGATAATACTAGATCGAGATATAATGGAAAAATTGTTGATAAAGAATCATTAGATATAGTTAATAAATTATTTGTTGAT
GATCAGATAGATAACCAAATTAGGCTTTACATTTCGGACATGAACCATACTACTATAGCAAGAACAAATACAATTATATATCC
GGCTATTATGAATCCAATAGATCACGAGTTTAATGAATATTTTTTGAATGCTCAGCTGATTGAAGAATTAAATACTGGAGTAA
TTATGAATATGATTCCAAGACAGCTAAGAGCCGATTCTAACTACAATTTTGCTTTAGAAAATTCGTTTAATCACACTGCTAGA
TATTTACCTCAGTTGCTAATGCAGGATAGGATAGCAATTCATGAAATAAGTTCACTATGGGATTCAATGACAACCGCAAATTA
CGTATTAGCTAGATCGGTTATTCCAGATTTGAAGGATTTATTACCAGCTGATGTTCAAATTACTGAGATGGCTGCTAATCTAA
ATTTGGAAGCATTAACCACACAAGTTGAGACAGCTTATTTATCAGGTATAACGACTGAATCTGCAAATGAATGTTTTAAAATA
ATTATAGCATCACTTCTATCTACACGTACTATATCAATGAGTTATTCCGGGAATAATTATGTTTCATTATTCTCTGGAATGTA
TTTACTATCAATTATACCATTTAATTCAATGCTACGTGAGTCAGTTATATCTCTACAGCTAGCAATTGTAAATTCAATTCTTT
ATCCAGCGTTTGGTCTACCACAACTTACGTATACATACCTAGATCAAGATACTCCATTTATGATAGCTCAACAAACTATTAGT
AATAGAAGAGTACGAGAATGGCTACAGCACGTTAATAACTTTGATTTTCCAAGAGTAAATAGAGACGGTGTATTTGTATATAC
TGTGCCAGATAGAATTAGGTATGGTAATATAGTAAACTTATTCTCTGAAACAGTGACAGAATTAGCGAATCAACAGTTTAGGA
CGTATACTTTAGAGTATCAAAGAGCTATCAAAAGAGCAATTCAGTTATTTGTTAGGCGTGTTCCACAAATACTAGATCTGACG
AGATTGATGTTTTACAACTATGAAGTTCTATTACGTATGATAGTAATGTCACAGCAACGAATGATAACTTTAACTACTGAAAA
ATTAGACTTAACTAGAGTAACATCTTTGCTATTCCTAATATCTAATACAGTTGTCTTTCCGGATCCTCAATCTTTAATGAGAT
ATTACTCAGCGAATAGGAATTTTCTGAATAACTATAATGAGCGTATTGATGATACTGTCGCTAGATTATATGCGTCAAATAGA
TTAAATTTGTATCGGAAGAAAGTATTATCAATTGTTACTGATTTTGTTAGGAACTTATATATTTTTGAAGCAACCAAGGTACC
AGCAGATCAAATGTATAATTTACGAGATAGATTAAGACGTCTACCGTTGGAAAACAGAAGGCAGAGAGTCTTTGATATTATGA
TGAATAATCAGGATCAGATAATTCATGCATCTGATAAAATAGCACACGGTGTTGTGTTGTTCAGAAATGAACGAGAATTAATT
AATGATGAATATGAAGGATTGACAAACGTAGTTCGTAACATAGATGGAAACGCACTATCTATTGAAGAGATACGTAATCGTGG
AGATTATCAACCACTCATCGATAGTTTATTACAAACTAATTCAGTTGCCCTCAAGGGAGTTATACCATTCAATACGACACATA
ATCCGTTCGAACTGATAGCAAAGGTCGATGTGTCAATTTTTGCACCGGTACTTAAGGATAGAGACATTAATAAATTAAAACCA
GTTAAATACGCAATAAACTCTGATTCACAGTCATTTTACATTGTCGCAAATAATAATTGGAAGCCGACATCATCAACTGCAGT
ATATAAGTTACAACCACGACAATTTGATTTTACACAGTCTCTATTTCAGCTTACGTCAAGACTATTTTTCCAAGTATTTAAAG

ATCCGCTTACTTTCCTAACTATACGAACTGTGGACCCGATAATTGCAGTCGCTAGTGACAACCGAAGGATTATCTTAAGTGTG

TAG

VP2-RVF

>NC_021626.1 Rotavirus F chicken/03V0568/DEU/2003 segment 2, complete sequence

ATGTCCAGTAATAAGGCAAAACAACGTTTAGAGGAATTAAAGAATAAAAGAGAAGCGTCTCTTAAAAAAGCAATAAATGAAGG
TGCTGAAGCAGTGAAAAAGTTAGTAGATCAACAGAATGACGAGAATTCAGTCCAATCTGAACAAACATTAAGTCAGTCAGACA
CTAAAACAAAAGAAGATAAAAAAGAGAAAGAGAATAGTCAGATACAGCAATCAGACGAAGTAAAATCATTAGAAGATGTGGAA
AAAGCAGCGAAAGAGAAAAGAGATGGGACTAGACATCTTATAGAAGTCCTTAAAACTAAAGAAGATAGTGAAAAAGAAATACA
GAAAGAAATATTACAGAAGGCAATACCAACTTATACACCAAATGATGAGGCAGTTAAAAAATTGTCTGAGATTAAAATTGAAG
AGAATGCGGAAATTAAACAATTATTTAGACTATTTGAGCCAGAAGTTGTTCCAATTTATGCAAACGATGGTAGAAAGACTTTA
ATTACTGCATGGTATTGGAAGCTTAAAAAAGATGATATACCAACTGGAGATTACACAGTTAGAGAATACTTTCTATCTCTATA
TACTCACGTCATTGATACAATGCCACCATATATAATGCTGAGAAGTATGGCTGTAGACAATAAGCATTCACGTGAAGCAGGTA
AAGTAGTAGATGCGGAAACTGCCTCAATTCTTGAAAAATTATTTGCAGATGAACAAACAGATGCAATGACAAGAAGATATATT
GCAGAAATGAGACATAGAGTTAACGCTGAAGCTAATATAGTTAGATATCAAGCAGTGCTGCATCCAGTTGACTATGAATTTAA
TCAATATTTCTTGGAGCATCAGATATTAAGACCACTAACAACACGTGAGATATTTCAGATGATACCAACACGTATGAGAAATG
ATGTTAATTATATATTTACTATCGATAATTCCTTTCTTGAAACAGCAAGATATGTTCCTTATAATCTGTTGCAAGATAGATTA
AATCTACATGAAGGTCTACAGTCTATATGGGAAACATTAACGTTAACTAATTATGTGCATGCCAGAACTGCAGTGCCTGATCT
AGTAGATCTTGTAGACACAGAAACACAAATTAAAGAAATGTCGCAAATGTTGCAATTAGAAGCGATGACAATACAGTCCGAAT
CACAATTTATAACAGGTATTAATTCTGACGCCGCAAATGAATTCTTTAAAACTGTTATAGCTGCTTGTTTGTCACAGCGCACT
TTAAGAATGGAATTTACTCAAGCGAATTATATGTCATTATTGTCTGGAATGTTTGTATTAGCTCTCGTTCCGAATGAACTTAT
TCTTCGTGAATCACTGAATTCTCTGCAAGTAGCGATAATAAATACAATTTTATGTCCAGCTTTTGACATTCCAGCTATGCAGT
ATAATTATTTAGAAATACAGACTCCATTTGAGCTCATTTTACCACGCATAGTTTCACGACAAGTCAGAGATTATTTACACCAT
GTCAACAATAACCACTTCCTATATAATGTAGTAGATGGAGTAAGAAATGCCTCTTTACAAAATATTATTAGATCAGGAGCGAT
AGTACAAAATGTAGCCGCTGCTTTGTTAAATATAGCAGGCCAACCATTTCGCGCGTATAATCAGGAGTATAAAAGATCAATAC
AAAGAGCAATTACTATATTAACGAGACGTGCGCCACAGATTGAAGATTCATTTAGGATGTTGATTTACAATTATGAAGTGATT
CAAAGATTCATAGTAATGAATCAGCAATATGCACAAACTATAAACACCGAATTAGTGAATATGACAGCAGCTACGTCGTATTT
CATGTTAATGTCAAATAGAGCTGTATATCCAGATGCATCTGTTCTACTACAGTATTATAAAGTGAACATCAGATTTTTGCATA
ATTATAACGAGGCAATTGATGATACCGTAGCTACCCTACTCGTATCTCATAGGTTAAATCTCTATCAGAAGAAAATACTGTCA
CTGGTAACTGAATTTATGCGTAAATTAAAAATATTTGATGCTCCAAAGATTCCACCAGATCAGATGTATTTACTAAGGAATAG
GATGAGGCAATTACCAGTTGAACAGAGAAGGGCTGATGTATACAATATAATGGAGACACATAGAACACAGATTGAACGCGCTT
CAAAACGAATTGCACAGGGAGTTGTATTAATGCAACAAGAAGCACCATTACAACATGACCCAATTGTCGGTTATACTAATGTA
ACAAGAAATTTAGATGGTTATAGAACCATCTCACTTGAAGATTTGCAGATGCGTGGAGATTTTCAACCATTAACGGACGTTTT
GTTAGCTAACCAACCAATTGCTTTACAAGGTGCAATAACGTACAAAACTGAAACTGATCCATTTGCTGTATTAGCTAAATCTG
ACGTTGCAGTATTTGCGCCAATAATAAAAGAGCGGAATCTAAATGCTTTAAATCCAATTTCATATGAAATAAATTCAGACTCA

AAAGGCTTCTATATAGTTCATAATAATTCATGGGTTCCGACATCAAATACTAAATTATTCAAACAACCGCCACGCAAATTTGT
TCTATCTGATTCAACATTCTTTTTAGAGTCTGGACTGTTTTACACTATATTTACCGATCCATTGTCATTTATATCACACACTT
CAGTTGAACCGGTTAATGCTGTAGCTTTTGATGGTCATAGAATCGTCAGAACGCTC**TAA**

VP2-RVG

>NC_021580.1 Rotavirus G chicken/03V0567/DEU/2003 segment 2, complete sequence

ATGGATCCCGAAGGTTTAATTTCAGATGCGATTAAAGCAATCGATGAAGCAAAGAATGATAAAAAGAAGATAATAGTAATAGC
TAAGCAGTTAGTCAACGATATTAAAGATACAGTTGAAGACTTGGATATTGATTTACTAAAAAAATTGAAAGTACAATTTAGCG
CAAAACGAATTGCAACAAAAGAAATTGATGTACTGATAAAAAATATTGAAGATAAAAAGAAAGAAAGTAAGAATGTGAGCGAT
AAAAAGGAGGAAAGCGGTAAAAACGATGATAATCAAAATCAGGAAAAAGGAAAGGATAATGAATCTGGAAAGAAAGAGAAAAT
CAATAACAGTAAAGGGGACGAAGGCGGACTAAGAGATAATGTACTTGGTAATTCAGATCCAGATAGTAAATTAACAAAAGATA
TATTGACAATAAGACAAATAAATTCCAAAATATTATTTGTTGATACTGAGAATGATACTTACTCAGTGTATATACCTGAACAA
ACTACGACACTCAAACCAATAGAAATAGAATTTAAACCAATACAAGACTATAAGCCAATAGCAGACTTAAGGATGGAGAAGAT
GATATTTTTGTCGAATAGGGATCAAGTGTCTGATCAAGAAGGACCAGAAGAGGTACTTTATACATCAGATTTCTTTAAGGATA
TAAAGAATGAAGATATTAAATACTTTAGAAATTACTTTTTGGAGAAGGCTATGGTGTTAAGGAAACAAATGCCAAACGTTAAT
TACATGTCAGGATTGAATAAAGAGACTAATCCATTGAATATACAAAATACAATATGCACATCATTCGATCAAATGAAATATTA
TAATATTGTAGTAGATAGAACTAATAGAGCATTTGACAATAGAAGAAGAGATGTCGAGTTTGACAATGTCACCATAGATGGGG
TGAATAGAAGAGCAACGGTATCTTTGAGACTACATCCAGTGGATGACCAAATTCTAGCAGCAGTTGATATGAACACATATGAA
ACACAAAACTTAGCTGATGTGATGACTAGATATCAAATGATTGCAGCGGATGGATATGCAGTTGCGCCAAAAATTCGAGTTGA
TAGAGATCATACAGTGATTGCAGACGTACGGTCACGTGTCTTAGCAAGAATATGCGTATTGTCACCTTATGTTTATAGATCAA
GAATACTAAACTCAGTATCGATGGTCAATAGATTATGGAGAACAAATGTCTTTTCTGAATCAATTGAAAATGCAAAAGACGCC
ATTTATAGATCAGCGGAAATCCAGTTTACGGTGGCAGATGCAACTACATCTGCACTATCTACTATTAACGTAGCCTCAGCACA
GCAAACGCTATCGGCAATACTAAATATGACAATATTCAGAATGGATATTGAACTAACCGGGAATCAATCATCTTTTGGAGCAG
CTATATCAGCTGCAATTGCGTTAATTATACTACCAACTGATGATGGAATGATGTCAGGAATGGTGTTTGATGATCTTTGCAAT
TTAGTTTTTAATGAATTGATAGCTTGGGCAACTGAAAGGCCAACTTTCGTTAAAAGGACAGGAGCTACTAATGCATTTGAAGC
TCACGTCAATTTAGGAGGTGGTAATTTGAATAGAGACATAATAAATTATATGAGATACGTATTACTCAGAAGACCATGGGCCA
TATTCCAAAGGACACAAACGGTGGATTATCAAACCGACATCATGTTACCAAATATAGACCCTGTAAATGTAAATGATCAGGCA
TATATGGCAATGAATAGCCTGTTAAGTGGTATCTCAGCCGCAGCACAAAGAAATCCAAATCCTGGTAGACAGATAGCGGCAAA
TTCTTTCAGAAAGCTGATTAAATCGATGAAAGACATGTGTTCAAATAATATTATGCCACTAATTAGACTGATACAGTTTAATG
TAGAAAGAGTAGCTCGAATTATGGAATTTCTGCCATATTCGGCGGATATTGGACAACTGAACCAAAATATGAGAGATGAAAGA
TTACGTGTTAAAATACCAGTTTCAGGTTTCCTGTCAATAATTATGGGAATAAATAAAGCGCCAGACAATTTCGATTGGGGAGC
CATGTTAGCATTCGCTGATGAGGTGAGAAGAGTGAATTATGCAGAGAGAGAGGCAATTGAAAATGTAACGACATATGTAGTCC
TGAAAAATGATGGAAATAGATCGGTATCAAAAAAAGATACGTTTCTGGAAAAAGTTGAAACACCTACTGAGACGATTAAAGCT
ATCACGAAAATACCTTCAGCATCACTAACTACAATCCTATCAGATAGATTACTCGTAAATGAGATAAGGAATACGAGAACATA
TGCACTCGTAAATAGATTAATTGACATATTGAGAACTGCATTCGATAATGTGCCGACTTCACATCATGGAATTGGTAAAGGAG

CATTGTTACTCCCGTATCCAAGAAGATTTGGCAGGTCATCAGTATATGTAAGAAAAGATAATATAATATTCAATAGACCGCAA
GGGGTTAACAGATACAACGTAGACGATTTACTACATGGAAGATTCTACCAAGGTTTAATGGGGCAAGTTCAGAATATGCAACC
AGTTTTTATTGATGGTCCAATGAAACTGAGAACATCAGATCCGACAGCAATTGAATCAATAACATCAGCATATTTGACGATGT
CAGCACCTTATGATGCTTATATACATCCAATGGATCTAAAGCATAATAAAGTTATTGAACCAAGAGAAGTTGATTTCTTTGTT
GATGATAAACCAACAAAACCGCATGAACAATTTGAAGAGATGATGTCTAAAACATCAGTTTTTATTCTAGATGCACAACGACT
GATTGTTCAGAGCGGAGCGACAAATTATAACTTTGATTATCACGATATGTTGATAACTGATAAAGTCGTTGACAAGCTACAGT
TCTCAACTGTGCTACCACCAGATGTAACCTTATTCAACGGTGTACTCGTATATGAAGCTAAACCTTCG**TGA**

VP2-RVH

>MT644972.1 Rotavirus H isolate SP-VC36 VP2 (VP2) gene, complete cds

ATGGAGGTGATAGACAAACTGAACGTGACACTAGAAAAACTCAAACAAGTTAAAGATAAGAAAGAATATCAGAATATCCATGA
CAATTTTTTACAGGATTTTGATTCTTTTGAAATTGTAGATATCAGTGATGATGAGTGGAAAGAATTTTTAAGACTAGTGAAAT
CAGTCATTTCAGTTTTAAAGTCAAATAAAATTAAAACTGTACCATTAGAAAATGAACTGAAACAGAAGGAAAAGCAGAGAGAT
AAAAATAAAAATGAAAAAGAGAATAAAGATGAAGAAAAGAATCAAAATGGAGGTAAAGATGAGCAGCAAGTCGAAGAATCCAC
GGATACGCTAATGGATAAAGTTCTAAAGGCAACAGCTTCTGGCACTACAAATCCATACTCATCAGATGTGCTTCAAATCAGAA
CAATCCTTTCGAAAACCTTATTTGTTGACACTGATACAGAAGCGTACTCAGTATATGTTCCAGAGCAACATGAACTTTCAGCT
GAACCAATTAAAATTGAATTGAAAACATTGGATAAATATAATACAAAAGTGAATATACTGAAGCAAGCGATAATTGTACCAAC
AAACAATCCGCTTTTAGCTGATACCTATGGCGCTCCAGAAATCTTATACTCAACTGACTTTTTCAATGACATCATGGAAAATT
CTAAAGAAGGATTGCAACTTTATTTCTTTGACAAAGCGCTTGGGTTAAAGAAAGAATTACCAAATCTACCTTTTATTTCGGCT
CTGTCCAAGGATGTGAATCCACTAAATCCACTAAACTCAATTTGCAAGTCATTTAATCAGGAGAAATATTATGATATGGTAAT
GGATAGAACTGACAGAGGACTTGATGTTAGAAGAGCGGCGGTACAATTTGACAACGTGATTGTGGATACTCAGAATAGAACTG
TCCAGTTCAACGTTAGGTTACATCCATTCGATTTACAGTTGATAAGAATTGCACAACAATTTGCAGAACCGATGCAGGATCTT
GCTCCAGTGATTAGAGAATATATGTTGCTAGGTGCTGATGGATACGTTATTACACAAAAAACTAGACTTGACAGAGACCAACA
ATTAATCGCAACAAGAAGATCGAATGTATTTGATAGGATGTGCGAACTATCTGGACCACTTTACAGATCTAGAATAATACATT
CAATGAGAATGATGTCAAAACTTTGGAGAACGAATGTTTTTAAGACATCACTTGAGAATGAAATTACAAACATTTACGCTGCA
GCTGAAGTTTCAATGGTTACAATCGATGCGACTACATCCGCTCTATCAACGATTAATATTGCGTCAGCTGAACAAACGTTGAA
TGCCCTACTAAATATGGCATTCTTTAGATGTGAATTGGATCTAATTGGTGGTCAGTCATCGTTTGGCGCAGCAATGTCAGCGA
TGTTAGCATTAGTTGTTTTGCCAACTAATCCAGAGAATATGGACGATGAGACTTTTGATATACTGTGTAATTTAGTTTACAAC
GAATTAATTGCCTGGGCAAATGATCGTCCTGTGTTTGTAAGAAGAGCTGGCGACACGAATGCATTTACAAGATTTGTAAACGC
TGGGTTGAATAGAGATATAACAAATTATATGAGATTTGTACTGTTGAGAAGGCCATGGTTGCCACTTGTTAACTCAAGAGATG
TTAGAAGGAATTGTCATGTTCTTGTACCAAATATTGATTTAGCTAACATCAACGATCAGAGTTATATGGCTGTGAATAATTTC
CTTAATGGGATAATTGAAGCATCAAGAAGGAATCCAAACCCAAACAAAACGATATCCGCAAATTCGTTCAGGAAACTGATGAA
AAACATGAGGGATATCTGTATTAATAAAATGATGCCGATGATTAGACTAATCAGATATAACGTTGAAAGAGTTGGTAGAATCA
TTAACTATCTTCCTTACACTGCAGACATTTATGACATTGATAGAGCACTAAGAGATGAGAGACTTAGAATTAAAATTCCAATG
TCAGGATTCCTATCGCTAATCATGGGTATAACAAAAGCGCCAGACGCTTTTGACTGGGCACAGTTGCTTACGTTTGCTGATGA

CGTAAGAAGAATGGATTTTGCAGAAGCGATTGCCGTTGAAGATTCCGCATCAGTCGCTATCATGAAAAATGATGCAAATCGTG
CTTTGTCAAAAAAAGAAATTTTCGTAAATGACATTAAACCGCCGACTCCAACAGTAGCGTCAATAGCGAGAATACCATCAGCG
ACTCTAACCGCAATCTTTTCAGATAGGCAGTTAGTCAATTTAATTAGGAACACACATTCATTTAGAACGATTAACGAAATTGC
TAATGCGCTACGGGCAGCATTTGATAATTCACCAACGTCACAGCATGGAATAGGAAAAGGAGCACTATTACATCCAACGCCAC
AAGAATTCGGAAGATCTTCACAGTTTGTTAGAAGAGACAACATAATCTTGCAACGACCAGCTGGTATCCAGAATTTTAATATC
GAAGATCTAAAGCAGGGCAGATATTTTCAAGGATTGACAGCACAAATTAGAGCAAGACAACCTGTGTTGATTAATGGACCAAT
CCCATTAAGAGTTTCTGATGCAGCAGACATTGAGCAAGTTACGTTGGCGTTTCTAACAATGAACTCCCCATATGATTCGTACA
TTGATCCAAAAGATTTGAGACAACAAAGACTATTATCAGACAGGGAAGTAGACCTGTTTATTGATCAAAACCCAGCACGACCA
AATGATGCGTTTGAAAACATAATGGCAAGAACGTCAGTCTTCGTGCTTGATGCACCAAGAGCAATAGTTCCAATTAACCCACA
AAGATTAAATTTACCGTATCATGATGTTATGGTCACGGATTCTGTGACAAAATTTCTAGAGTTTACAGTTGCTTTAACACCTG
ATTTGCAGCTATTCAATGGATTACTAGTGTTTGAACAG**TAG**

VP2-RVI

>NC_026826.2 Rotavirus I strain KE135/2012 VP2 gene, complete cds

ATGTCACTAATAGGGACGCTCGAGAAGTACCTCTCAGCACTTGAAGAAATAAAAACAAAAGGAGGACTACAAAAAATACATGA
TGATTTGCTACATGAATTAGACATGTTTATTGATGATATAGACATTAAAGAAGAGAAAGAAACCAAAATTTTGTTCGAAAAAA
TAAGCAGACTATTAAAAAAGAAGGGCATAAAGACAACGCCAATAGAAAATGAATTTAATTCAAAAAAATTCACAGAAAAGAAT
GGCACTGCAGAAACAGACAATATGAGCAAAAGAACAGAAGAAACTATGACTGAAAAAGAAAATGGAAATGCTAACACCACACA
ACATAACGTGAAGAGTGATGGTGAAAAGCGACATGACGACAATTCTAATATCAATTCATATATTCAAAATGTCATAAACATTA
GAGATATTTTATCCAAAACATTATTCGTCGATACTGACGCAGATGACTATGCAATATACCTTCCATCAGAAGTTCCGGAATCA
ATAAAACCAATCCAGATCGACGTTATACCTATCAATATGTTTGAGACATTTGCTAACATGAGAAAAATCAATACAATAAACAT
TTCAAACAATGACTTGGTATCTGACACATACGGTCCAGCAGAAGTATTGTATGACAGCATATTTTTCTCAGATATGGACCTTG
AAAAGTACGGTAACATTGAAAAATACTTCTTATACAAAGCTTCAAACGTATCACGGAAATTGCCAAATGTTAATTATATGACC
GAATGCACAAAAAATTCCAAACCCATATAACAACGATAACACAATTACGACGATGTTTGGACAAGGGACGTATTATGACATGCT
AATGGATCGTACAGATAAGAGTTTGACTGGAAGAAGAACTGATGCTCAGTATGATGATATTTCTGTTGATGCAATAAACAGAC
AAATATCCATATCACTACGGATACATCCAGTAGATGATCAGCTACTCAACATAGCCATAAACAATTGCATCCAGACACAGCCA
TTACAACCGGTCTTAAATGAATACATTATGATAGCGGCTGATGGATATGTTGCATCCCCTAAAAAACGTTATGATAGGAACAC
ATTGACTATATCAAATGTTTTTTCACCAGTTTTCAATAGACTGTGTGTGTTATCAGGCACAACATACAGAGCTAGAACTTTAC
AATCTATGACTTTTATGTCGAGACTGTGGAAAACCAATGTTTTCAAAACATCACTAGAGGATGATATAGCTAAAATGTATGCT
GGAGCCGAAATTTCAATGACTACGATCGATGGAACAACAGCCAGTCTTGCTACAATAAACATTTCTGCGGCCGAACAAGCTTT
AATAGGTATACTAAATGCGTCATTTTTTAGATTCGACTTTAACTTAACTGGACCTCAAAACTCGTTAGGAGCTGCGATATCGG
CGTTAATAGCGTTGATAGTACTACCAATTGACCAAGATACAATGGATAATGATACATATGATATGTTGTGTAACACTGTTTTT
AATGAAATGATGGCAAATGCAATGAACCTACCAGCGTTTGTGCGAAGAGCTGGAGATGAAAATGCGTTTAGAAGATATGTGAA
TGGACCAATTCCGAGAGAAGCTTCTGCTTTTATTAGATTCATTTTGTTACGAAGAGGATGGTTGCTTTTTCAGAGATCTGATG
ATAGAACGATTCATTGTGACATCTTAGTACCAAATTGTGACACGCAGAATGTAAATGATCAACCATATGTTTTACTCTCAGAC

TTCTTCGCTGCGATATTGGAATCTTCCAGACGTAATCCAAATCCAGGAAAAAATACATCCGCTAATTCGTTTAGACGACTAAT
TCGAGGACTTAGGGACATTGTCTGTAACAAAATCATGCCAGCTATGAGGTTAATTAGGTACAATGTAGAAAGAATAGCTAGAA
TTCAAAGTATGTTACCATATTCTGCTGATCTTGCATTAGTTAACCCCTCTTTGAGAGATGAAAGATTGAGAGCAAACATCCCA
TTATCAGGATTTTTATCATTGTTAATGGGAATATCAAAGGCACCAGATGCATTTGATTGGACAACAGTATTAACATTTTGTGA
TTCAATGCGAAAATTAAACTATGCTGAAACAATATCGGTCGAAGAAGCTCTAACCGTTGCTATTTCATCACATGATATAGATA
AATCAGTATCAAAAAAAGACATTATAAAAGACGTATTGCACCCACCAACTTCAGCCGTAGCTGCGATTACAAAGGTTCCATCA
GCGTCACTTTCTGCATTGCTATCAGATACGGTTTTAATTAACCTGGTAAAAAACTCAAAACCATTTAGGAAAATTACAGACAT
AGTGAAAGTTTTGAAAGCAGCTTTTCAACATTCACCGACTGCGAGTTATGGAGTAGTCAAAGGCGCTCTATTGATGTCATCAC
CCCAAAATTTTAGGAGATCATCTCAATATGTTAAAAGAGATAATGTAATACACAATGTCGTAGATGGCATTCAAAAATTCAAA
GCAGAAGATCTAGTTAAAGGCAATCACTTTCCTGGTCTAATTAATTCAATAAAGAACGGTGATGATATATATATAGAAGGACC
TTTACCAGTAAGAACATCGCATTCATCGGAGGTCGCTACCGCTTCTTTCGCCTTTCTAACAATGAATTCACCATATGACGCCT
TCATTGATCCAACTGATTTACAACATCAAAGATTAATAAAAATTAGAGCAGTTGACCATTTTTCAGACTCGTCAATTGACATG
ATACCATCGAAATTTGACAATTTGCTAGCTAAGACATCAGTATTCGTACATGATGCTCAAAGCTTAATGGTACAGTCAAAATC
AACAGTGAGACGGTTCAACTACCACGAATCACTATTACATATTGACATAGCAAATCTGATTAACTTCTCAGTTGCTTTGCCAA
CTGAACTACAGCTATTCGATGGAACATTAGTGTATGATGTG**TAG**

VP2-RVJ

>NC_055265.1 Rotavirus J strain BO4351/Ms/2014 VP2 (VP2) gene, complete cds

ATGGAGTCGATTACTCTCTTCACCGCAAAGCTCGATGAAGTCAAACCACTCAAAGAAAAAGCGGATATACAACAACTTTATGA
TGAGATAGTACAAAAGTGGGATGAAATTGAAATTGTTGATCTCAACGAAGATGACTACAAGGAATTGCTTCGGTTAGTTAAAT
CATTAGAGTCAGTTTTCAAAGTCAAGGGTATAAATGTTAATACTCTAAGAAATGAGACTCAACAAAAGCAGAAACAGTGGGAC
GAGAAAATTAAAAAAGAAAATGAAAGTGAGAAAGAAGCGCAAAATACTGACAACGATCGGAACGAAGAAGAGACGCAGAATTC
AAATTCGGACAATACGCCTGATTTAATGGACAAAATTCTAAAATATTCTGCTCAAGGCAACACCAACCCATTCACGAATGATG
TTCTCCAAATACGGTCAATACTGTCGAAAACACTTTTCGTTGACACAGACAATGAGTCATATTCGGTGTATGTTCCTGAAGCT
TCAACCTATAAAGTTTCACCAATTGAGATAACTTTCAAGACCATTGACACGTTCGATCCGAAAACTAAAATAACCAGAAAACA
AACAATAATATTATCGAACAATAATCAGGTAGCCGATGCACTTGGGCCAGCTGACGTCTTATACACATCATCATTTTTCGATG
ATATGACGTCGACTTCAATCAAAGAACTGGATTTATACTTCCTCGATAAAGCGCTGGGATTGAAGAAAGAGCTGCCAAATTTA
AATTATATTTCAAAATTGGACAAAGAAGTCAATCCAATGAATGTGGCAAACACGGTAACAACATCCTTTAACCAGAAGGAATA
TTACGACATGGTTATGGATAGACAAGACAGATCACTTGACGCGCGTAGGCAGGCAATTGAATTCGACAACGTCGTTGTTGACG
CACAAAGAAGAACTGTGAGATTCCCAGTCCGTTTGCATCCATTCGACAACCAGCTAATTGATATAGCTGCGAATTATGCTGTC
GAAACGCAAGATCTTGCGAACGCAATGAGAGAGTACGCAATGTTAGGAGCGGATGGATATGTGATTACGCCTAAGGTTAGAGT
TGACAGAGATCAAAGGTTAATATACAACAGAAGATCTCAGGTGTTGAATAGGCTGTGTGAACTATCAGGGCTGGTATACAGAA
CAAGAATTCTACACTCAATGAGATTAATGACCCCTCTTTGGAGGACAAATGTTTTTCAAACTTCACTCGAGGATGAAATAACA
AGAATATATTCCGCAGCCGAAGTATCGATGGTCGCAATAGATGCTACAGTATCCGCACTGTCATCTATTAATATAGGCGTAGC
TAAGCAAACTCTAGACGCTCTCTTGAATATGTCTTTCTTTAGATGTGAATTGGAACTGGTTGGATCACAATCATCATTCGGAG

CAGCGCTGTCTGCCGCGATAGCGTTACTAGTCCTCCCAACAGATCAAGATCATATGGATGAGGAAGTCTTTGACATTTTGTGT
AATTTAGTCTATAACGAATTAATTGCCTGGCCATCAGATCTCCCATCATTCGTAAGAAGAAACGGCGCCACAAACGCCTTCAG
ACAATATGTGAACGCTGGAGTCAATAGGGAAATCGCTGCATACATGCGTCACGTCTTACTTCGCAGGCCATGGCTACCTCTCA
TTCAATCAAATGACATCAGAAGGCAGTGTCACGTTTTAGTTCCAAACATTGATCTAGCTAACGTGAATGATCCAACATACGTT
GCATTGAATGGACTGCTCAATGGAATTATAAACGCATCCCGTAGGAATCCAAATCCTGGCAGATCAATTAACGCAAACTCCTT
TAGAAAGCTGTTAAAGAATTTGAGAGATATATGTGTTAATAAACTCATGCCAGCGGTTCGGCTGTTAAGATATAACGTTGAAC
GAGTGGCGAGAATTTTCCAATTTCTACCATATTCAGCAGATCTTTTTGATGCGAATCCGAACATGCGCGATGAGCGCCTAAGA
ATTAAATTTCCAATTTCCGGCGTTCTGTCATTATTGATGGGCATTACGAAAGCACCTGATGCGTTCGACTGGGCTGCACTTTT
AAATTTCGCTGATGATATCAGAAAACTGGACTATGCGGAGGCGGAAGCAACGGAGGATGCAGCAACAATCGCTGTTCTAAAAA
ATGATATAAACAGGTCAGTTTCAAGAAAAGACGTTTGGAACGCAGAGGTTAAACCGCCGTCACCAACAATTGCGTCAGTTGCT
AAAATTCCATCCGCTTCACTATCCGCAATTTTGTCGGACAGACAATTGATCAATCTTGTCAGAAACACCCATTCCTTTAGAAT
GATTACCGAAATCGTTAACGCTTTAAGGGCCGCATTTGAAAATTCTCCAACTTCGCAACATGGAATAGGTAAAGGCGCATTGT
TAAACCCAGTTCCAACACCATTCGGCAGATCATCTCAATATGTTAGAAGAGATAACGTCATTTTCCAGAGGCCGCCAAATGTT
CAAATGTTTACAATTCAACAGCTCATGCAAGGTCAACATTTTGCTGGATTAGTCGCACAGATTCGCGCTAGGAGACCTATTTT
CATACAAGGTCCAGTTCAATTAAGAGTTGCAAACGCAAGAGATGTGGAACAAGTTACAATGGCGTATCTAACCATGAATTCGC
CATATGACGCCTTTATCAATCCAATTGACTTAAGGCAACAGAGAATGATTGAACCAAGAGAGGTTGATTTGTTCATTGACGAC
GAGCTTAATCGAATCGAGGATGACTTTGACAATGTGATGGCGCAGACTTCGGTATACGTCCTCGATGCACAGAGATTACTCGT
CCCAATTCAGGCTCAATTAAGAAATTTCGACTACCATGATATAATGATCACAGATTCAGTCACTAAGCATCTCACTATGACCG
TCGCACAACCGCCAGACTTACAACTGTTTAACGGGCTTTTGGTTTTTGAGCAG**TAA**

Table S1. Predicted molecular weight of Flag-VP2 of RV species A to J.

Flag-VP2	Predicted molecular weight (kDa)
RVA	103.6
RVB	106.9
RVC	102.7
RVD	107.1
RVF	105.3
RVG	113.7
RVH	113.0
RVI	111.6

RVJ	113.4

Table S2. Synthetic DNA fragments used in this study.

**Name**	**Sequence**
MluI-Flag-VP2/D-NotI	5'-ACGCGTgtcgacc**ATG**[GATTACAAGGATGACGACGATAAG][CGAAAGAATAAACTAAT<br>ACAAGATAAAGAGAGAGAAAAATCTAAATCTAAAGATGAAACAAGAACCGAGCGAGGAAAGACT<br>GATAGAAACACTACAAATTCGTTAAAAAATGGAGTAGAGACGGATTGTAATGATGGTAACTGCG<br>TGGACAATGTTAATGTAGATAGTAAAAATGATAAAAAAGAAGAGAGAACGAAAAAAGATTACAT<br>TAAAAGTGAAAAAAAGAGTGACGGTGCATCTGATACTAAAGAGAAAGAAAATGGATCTAAGGAT<br>AAAGTTGTATCAAATGAAGAGAATAAGAAAAATGAAAAGGAGCTAGCGGAAATTCTGAAAACAA<br>AAGAAGAAGTTACTAAAGACTTAGATAAAGAAATATTTTATAAATATATACCGACATTTGATGT<br>TAATATAGATATTGTTAAGAAAATGTTAGATATACCATCTGTTTCGCCAAAGGATGAGAAAACA<br>TTATTTAGATTATTTGATTTAAAAAACCTGCCATTGTATGATACTTCAGCAGTTAGGACACTAG<br>AAACGAGATGGGTGTGGAAGCTTAAAAAAGATGATTTGCCAGATGATTCTTACTCAGTGCGTGA<br>GTACTTTCAAGGTTTATATGAACACATTCTATCAATAATACCAGATTATATAATGTTGAGGAAT<br>ATGGCTGTGGATAATACTAGATCGAGATATAATGGAAAAATTGTTGATAAAGAATCATTAGATA<br>TAGTTAATAAATTATTTGTTGATGATCAGATAGATAACCAAATTAGGCTTTACATTTCGGACAT<br>GAACCATACTACTATAGCAAGAACAAATACAATTATATATCCGGCTATTATGAATCCAATAGAT<br>CACGAGTTTAATGAATATTTTTTGAATGCTCAGCTGATTGAAGAATTAAATACTGGAGTAATTA<br>TGAATATGATTCCAAGACAGCTAAGAGCCGATTCTAACTACAATTTTGCTTTAGAAAATTCGTT<br>TAATCACACTGCTAGATATTTACCTCAGTTGCTAATGCAGGATAGGATAGCAATTCATGAAATA<br>AGTTCACTATGGGATTCAATGACAACCGCAAATTACGTATTAGCTAGATCGGTTATTCCAGATT<br>TGAAGGATTTATTACCAGCTGATGTTCAAATTACTGAGATGGCTGCTAATCTAAATTTGGAAGC<br>ATTAACCACACAAGTTGAGACAGCTTATTTATCAGGTATAACGACTGAATCTGCAAATGAATGT<br>TTTAAAATAATTATAGCATCACTTCTATCTACACGTACTATATCAATGAGTTATTCCGGGAATA<br>ATTATGTTTCATTATTCTCTGGAATGTATTTACTATCAATTATACCATTTAATTCAATGCTACG<br>TGAGTCAGTTATATCTCTACAGCTAGCAATTGTAAATTCAATTCTTTATCCAGCGTTTGGTCTA<br>CCACAACTTACGTATACATACCTAGATCAAGATACTCCATTTATGATAGCTCAACAAACTATTA<br>GTAATAGAAGAGTACGAGAATGGCTACAGCACGTTAATAACTTTGATTTTCCAAGAGTAAATAG<br>AGACGGTGTATTTGTATATACTGTGCCAGATAGAATTAGGTATGGTAATATAGTAAACTTATTC<br>TCTGAAACAGTGACAGAATTAGCGAATCAACAGTTTAGGACGTATACTTTAGAGTATCAAAGAG<br>CTATCAAAAGAGCAATTCAGTTATTTGTTAGGCGTGTTCCACAAATACTAGATCTGACGAGATT<br>GATGTTTTACAACTATGAAGTTCTATTACGTATGATAGTAATGTCACAGCAACGAATGATAACT<br>TTAACTACTGAAAAATTAGACTTAACTAGAGTAACATCTTTGCTATTCCTAATATCTAATACAG<br>TTGTCTTTCCGGATCCTCAATCTTTAATGAGATATTACTCAGCGAATAGGAATTTTCTGAATAA<br>CTATAATGAGCGTATTGATGATACTGTCGCTAGATTATATGCGTCAAATAGATTAAATTTGTAT<br>CGGAAGAAAGTATTATCAATTGTTACTGATTTTGTTAGGAACTTATATATTTTTGAAGCAACCA<br>AGGTACCAGCAGATCAAATGTATAATTTACGAGATAGATTAAGACGTCTACCGTTGGAAAACAG<br>AAGGCAGAGAGTCTTTGATATTATGATGAATAATCAGGATCAGATAATTCATGCATCTGATAAA<br>ATAGCACACGGTGTTGTGTTGTTCAGAAATGAACGAGAATTAATTAATGATGAATATGAAGGAT<br>TGACAAACGTAGTTCGTAACATAGATGGAAACGCACTATCTATTGAAGAGATACGTAATCGTGG<br>AGATTATCAACCACTCATCGATAGTTTATTACAAACTAATTCAGTTGCCCTCAAGGGAGTTATA

	CCATTCAATACGACACATAATCCGTTCGAACTGATAGCAAAGGTCGATGTGTCAATTTTTGCAC<br>CGGTACTTAAGGATAGAGACATTAATAAATTAAAACCAGTTAAATACGCAATAAACTCTGATTC<br>ACAGTCATTTTACATTGTCGCAAATAATAATTGGAAGCCGACATCATCAACTGCAGTATATAAG<br>TTACAACCACGACAATTTGATTTTACACAGTCTCTATTTCAGCTTACGTCAAGACTATTTTTCC<br>AAGTATTTAAAGATCCGCTTACTTTCCTAACTATACGAACTGTGGACCCGATAATTGCAGTCGC<br>TAGTGACAACCGAAGGATTATCTTAAGTGTG]**TAG**GCGGCCGC-3′
MluI-Flag-<br>VP2/G-NotI	5′-ACGCGTgtcgacc**ATG**[GATTACAAGGATGACGACGATAAG][GATCCCGAAGGTTTAATT<br>TCAGATGCGATTAAAGCAATCGATGAAGCAAAGAATGATAAAAAGAAGATAATAGTAATAGCTA<br>AGCAGTTAGTCAACGATATTAAAGATACAGTTGAAGACTTGGATATTGATTTACTAAAAAAATT<br>GAAAGTACAATTTAGCGCAAAACGAATTGCAACAAAAGAAATTGATGTACTGATAAAAAATATT<br>GAAGATAAAAAGAAAGAAAGTAAGAATGTGAGCGATAAAAAGGAGGAAAGCGGTAAAAACGATG<br>ATAATCAAAATCAGGAAAAAGGAAAGGATAATGAATCTGGAAAGAAAGAGAAAATCAATAACAG<br>TAAAGGGGACGAAGGCGGACTAAGAGATAATGTACTTGGTAATTCAGATCCAGATAGTAAATTA<br>ACAAAAGATATATTGACAATAAGACAAATAAATTCCAAAATATTATTTGTTGATACTGAGAATG<br>ATACTTACTCAGTGTATATACCTGAACAAACTACGACACTCAAACCAATAGAAATAGAATTTAA<br>ACCAATACAAGACTATAAGCCAATAGCAGACTTAAGGATGGAGAAGATGATATTTTTGTCGAAT<br>AGGGATCAAGTGTCTGATCAAGAAGGACCAGAAGAGGTACTTTATACATCAGATTTCTTTAAGG<br>ATATAAAGAATGAAGATATTAAATACTTTAGAAATTACTTTTTGGAGAAGGCTATGGTGTTAAG<br>GAAACAAATGCCAAACGTTAATTACATGTCAGGATTGAATAAAGAGACTAATCCATTGAATATA<br>CAAAATACAATATGCACATCATTCGATCAAATGAAATATTATAATATTGTAGTAGATAGAACTA<br>ATAGAGCATTTGACAATAGAAGAAGAGATGTCGAGTTTGACAATGTCACCATAGATGGGGTGAA<br>TAGAAGAGCAACGGTATCTTTGAGACTACATCCAGTGGATGACCAAATTCTAGCAGCAGTTGAT<br>ATGAACACATATGAAACACAAAACTTAGCTGATGTGATGACTAGATATCAAATGATTGCAGCGG<br>ATGGATATGCAGTTGCGCCAAAAATTCGAGTTGATAGAGATCATACAGTGATTGCAGACGTACG<br>GTCACGTGTCTTAGCAAGAATATGCGTATTGTCACCTTATGTTTATAGATCAAGAATACTAAAC<br>TCAGTATCGATGGTCAATAGATTATGGAGAACAAATGTCTTTTCTGAATCAATTGAAAATGCAA<br>AAGACGCCATTTATAGATCAGCGGAAATCCAGTTTACGGTGGCAGATGCAACTACATCTGCACT<br>ATCTACTATTAACGTAGCCTCAGCACAGCAAACGCTATCGGCAATACTAAATATGACAATATTC<br>AGAATGGATATTGAACTAACCGGGAATCAATCATCTTTTGGAGCAGCTATATCAGCTGCAATTG<br>CGTTAATTATACTACCAACTGATGATGGAATGATGTCAGGAATGGTGTTTGATGATCTTTGCAA<br>TTTAGTTTTTAATGAATTGATAGCTTGGGCAACTGAAAGGCCAACTTTCGTTAAAAGGACAGGA<br>GCTACTAATGCATTTGAAGCTCACGTCAATTTAGGAGGTGGTAATTTGAATAGAGACATAATAA<br>ATTATATGAGATACGTATTACTCAGAAGACCATGGGCCATATTCCAAAGGACACAAACGGTGGA<br>TTATCAAACCGACATCATGTTACCAAATATAGACCCTGTAAATGTAAATGATCAGGCATATATG<br>GCAATGAATAGCCTGTTAAGTGGTATCTCAGCCGCAGCACAAAGAAATCCAAATCCTGGTAGAC<br>AGATAGCGGCAAATTCTTTCAGAAAGCTGATTAAATCGATGAAAGACATGTGTTCAAATAATAT<br>TATGCCACTAATTAGACTGATACAGTTTAATGTAGAAAGAGTAGCTCGAATTATGGAATTTCTG<br>CCATATTCGGCGGATATTGGACAACTGAACCAAAATATGAGAGATGAAAGATTACGTGTTAAAA<br>TACCAGTTTCAGGTTTCCTGTCAATAATTATGGGAATAAATAAAGCGCCAGACAATTTCGATTG<br>GGGAGCCATGTTAGCATTCGCTGATGAGGTGAGAAGAGTGAATTATGCAGAGAGAGAGGCAATT<br>GAAAATGTAACGACATATGTAGTCCTGAAAAATGATGGAAATAGATCGGTATCAAAAAAAGATA<br>CGTTTCTGGAAAAAGTTGAAACACCTACTGAGACGATTAAAGCTATCACGAAAATACCTTCAGC<br>ATCACTAACTACAATCCTATCAGATAGATTACTCGTAAATGAGATAAGGAATACGAGAACATAT<br>GCACTCGTAAATAGATTAATTGACATATTGAGAACTGCATTCGATAATGTGCCGACTTCACATC<br>ATGGAATTGGTAAAGGAGCATTGTTACTCCCGTATCCAAGAAGATTTGGCAGGTCATCAGTATA

	TGTAAGAAAAGATAATATAATATTCAATAGACCGCAAGGGGTTAACAGATACAACGTAGACGAT<br>TTACTACATGGAAGATTCTACCAAGGTTTAATGGGGCAAGTTCAGAATATGCAACCAGTTTTTA<br>TTGATGGTCCAATGAAACTGAGAACATCAGATCCGACAGCAATTGAATCAATAACATCAGCATA<br>TTTGACGATGTCAGCACCTTATGATGCTTATATACATCCAATGGATCTAAAGCATAATAAAGTT<br>ATTGAACCAAGAGAAGTTGATTTCTTTGTTGATGATAAACCAACAAAACCGCATGAACAATTTG<br>AAGAGATGATGTCTAAAACATCAGTTTTTATTCTAGATGCACAACGACTGATTGTTCAGAGCGG<br>AGCGACAAATTATAACTTTGATTATCACGATATGTTGATAACTGATAAAGTCGTTGACAAGCTA<br>CAGTTCTCAACTGTGCTACCACCAGATGTAACCTTATTCAACGGTGTACTCGTATATGAAGCTA<br>AACCTTCG]**TGA**GCGGCCGC-3'
MluI-Flag-<br>VP2/H-NotI	5'-GATCACGCGTGTCGACC**ATG**[GATTACAAGGATGACGACGATAAG][GAGGTGATAGACAA<br>ACTGAACGTGACACTAGAAAAACTCAAACAAGTTAAAGATAAGAAAGAATATCAGAATATCCAT<br>GACAATTTTTTACAGGATTTTGATTCTTTTGAAATTGTAGATATCAGTGATGATGAGTGGAAAG<br>AATTTTTAAGACTAGTGAAATCAGTCATTTCAGTTTTAAAGTCAAATAAAATTAAAACTGTACC<br>ATTAGAAAATGAACTGAAACAGAAGGAAAAGCAGAGAGATAAAAATAAAAATGAAAAAGAGAAT<br>AAAGATGAAGAAAAGAATCAAAATGGAGGTAAAGATGAGCAGCAAGTCGAAGAATCCACGGATA<br>CGCTAATGGATAAAGTTCTAAAGGCAACAGCTTCTGGCACTACAAATCCATACTCATCAGATGT<br>GCTTCAAATCAGAACAATCCTTTCGAAAACCTTATTTGTTGACACTGATACAGAAGCGTACTCA<br>GTATATGTTCCAGAGCAACATGAACTTTCAGCTGAACCAATTAAAATTGAATTGAAAACATTGG<br>ATAAATATAATACAAAAGTGAATATACTGAAGCAAGCGATAATTGTACCAACAAACAATCCGCT<br>TTTAGCTGATACCTATGGCGCTCCAGAAATCTTATACTCAACTGACTTTTTCAATGACATCATG<br>GAAAATTCTAAAGAAGGATTGCAACTTTATTTCTTTGACAAAGCGCTTGGGTTAAAGAAAGAAT<br>TACCAAATCTACCTTTTATTTCGGCTCTGTCCAAGGATGTGAATCCACTAAATCCACTAAACTC<br>AATTTGCAAGTCATTTAATCAGGAGAAATATTATGATATGGTAATGGATAGAACTGACAGAGGA<br>CTTGATGTTAGAAGAGCGGCGGTACAATTTGACAACGTGATTGTGGATACTCAGAATAGAACTG<br>TCCAGTTCAACGTTAGGTTACATCCATTCGATTTACAGTTGATAAGAATTGCACAACAATTTGC<br>AGAACCGATGCAGGATCTTGCTCCAGTGATTAGAGAATATATGTTGCTAGGTGCTGATGGATAC<br>GTTATTACACAAAAAACTAGACTTGACAGAGACCAACAATTAATCGCAACAAGAAGATCGAATG<br>TATTTGATAGGATGTGCGAACTATCTGGACCACTTTACAGATCTAGAATAATACATTCAATGAG<br>AATGATGTCAAAACTTTGGAGAACGAATGTTTTTAAGACATCACTTGAGAATGAAATTACAAAC<br>ATTTACGCTGCAGCTGAAGTTTCAATGGTTACAATCGATGCGACTACATCCGCTCTATCAACGA<br>TTAATATTGCGTCAGCTGAACAAACGTTGAATGCCCTACTAAATATGGCATTCTTTAGATGTGA<br>ATTGGATCTAATTGGTGGTCAGTCATCGTTTGGCGCAGCAATGTCAGCGATGTTAGCATTAGTT<br>GTTTTGCCAACTAATCCAGAGAATATGGACGATGAGACTTTTGATATACTGTGTAATTTAGTTT<br>ACAACGAATTAATTGCCTGGGCAAATGATCGTCCTGTGTTTGTAAGAAGAGCTGGCGACACGAA<br>TGCATTTACAAGATTTGTAAACGCTGGGTTGAATAGAGATATAACAAATTATATGAGATTTGTA<br>CTGTTGAGAAGGCCATGGTTGCCACTTGTTAACTCAAGAGATGTTAGAAGGAATTGTCATGTTC<br>TTGTACCAAATATTGATTTAGCTAACATCAACGATCAGAGTTATATGGCTGTGAATAATTTCCT<br>TAATGGGATAATTGAAGCATCAAGAAGGAATCCAAACCCAAACAAAACGATATCCGCAAATTCG<br>TTCAGGAAACTGATGAAAAACATGAGGGATATCTGTATTAATAAAATGATGCCGATGATTAGAC<br>TAATCAGATATAACGTTGAAAGAGTTGGTAGAATCATTAACTATCTTCCTTACACTGCAGACAT<br>TTATGACATTGATAGAGCACTAAGAGATGAGAGACTTAGAATTAAAATTCCAATGTCAGGATTC<br>CTATCGCTAATCATGGGTATAACAAAAGCGCCAGACGCTTTTGACTGGGCACAGTTGCTTACGT<br>TTGCTGATGACGTAAGAAGAATGGATTTTGCAGAAGCGATTGCCGTTGAAGATTCCGCATCAGT<br>CGCTATCATGAAAAATGATGCAAATCGTGCTTTGTCAAAAAAAGAAATTTTCGTAAATGACATT<br>AAACCGCCGACTCCAACAGTAGCGTCAATAGCGAGAATACCATCAGCGACTCTAACCGCAATCT

	TTTCAGATAGGCAGTTAGTCAATTTAATTAGGAACACACATTCATTTAGAACGATTAACGAAAT<br>TGCTAATGCGCTACGGGCAGCATTTGATAATTCACCAACGTCACAGCATGGAATAGGAAAAGGA<br>GCACTATTACATCCAACGCCACAAGAATTCGGAAGATCTTCACAGTTTGTTAGAAGAGACAACA<br>TAATCTTGCAACGACCAGCTGGTATCCAGAATTTTAATATCGAAGATCTAAAGCAGGGCAGATA<br>TTTTCAAGGATTGACAGCACAAATTAGAGCAAGACAACCTGTGTTGATTAATGGACCAATCCCA<br>TTAAGAGTTTCTGATGCAGCAGACATTGAGCAAGTTACGTTGGCGTTTCTAACAATGAACTCCC<br>CATATGATTCGTACATTGATCCAAAAGATTTGAGACAACAAAGACTATTATCAGACAGGGAAGT<br>AGACCTGTTTATTGATCAAAACCCAGCACGACCAAATGATGCGTTTGAAAACATAATGGCAAGA<br>ACGTCAGTCTTCGTGCTTGATGCACCAAGAGCAATAGTTCCAATTAACCCACAAAGATTAAATT<br>TACCGTATCATGATGTTATGGTCACGGATTCTGTGACAAAATTTCTAGAGTTTACAGTTGCTTT<br>AACACCTGATTTGCAGCTATTCAATGGATTACTAGTGTTTGAACAG]**TAG**GCGGCCGCGATC-<br>3'
MluI-Flag-<br>VP2/I-NotI	5'-GATCACGCGTGTCGACC**ATG**[GATTACAAGGATGACGACGATAAG][TCACTAATAGGGAC<br>GCTCGAGAAGTACCTCTCAGCACTTGAAGAAATAAAAACAAAAGGAGGACTACAAAAAATACAT<br>GATGATTTGCTACATGAATTAGACATGTTTATTGATGATATAGACATTAAAGAAGAGAAAGAAA<br>CCAAAATTTTGTTCGAAAAAATAAGCAGACTATTAAAAAAGAAGGGCATAAAGACAACGCCAAT<br>AGAAAATGAATTTAATTCAAAAAAATTCACAGAAAAGAATGGCACTGCAGAAACAGACAATATG<br>AGCAAAAGAACAGAAGAAACTATGACTGAAAAAGAAAATGGAAATGCTAACACCACACAACATA<br>ACGTGAAGAGTGATGGTGAAAAGCGACATGACGACAATTCTAATATCAATTCATATATTCAAAA<br>TGTCATAAACATTAGAGATATTTTATCCAAAACATTATTCGTCGATACTGACGCAGATGACTAT<br>GCAATATACCTTCCATCAGAAGTTCCGGAATCAATAAAACCAATCCAGATCGACGTTATACCTA<br>TCAATATGTTTGAGACATTTGCTAACATGAGAAAAATCAATACAATAAACATTTCAAACAATGA<br>CTTGGTATCTGACACATACGGTCCAGCAGAAGTATTGTATGACAGCATATTTTTCTCAGATATG<br>GACCTTGAAAAGTACGGTAACATTGAAAAATACTTCTTATACAAAGCTTCAAACGTATCACGGA<br>AATTGCCAAATGTTAATTATATGACCGAATGCACAAAAATTCCAAACCCATATAACAACGATAA<br>CACAATTACGACGATGTTTGGACAAGGGACGTATTATGACATGCTAATGGATCGTACAGATAAG<br>AGTTTGACTGGAAGAAGAACTGATGCTCAGTATGATGATATTTCTGTTGATGCAATAAACAGAC<br>AAATATCCATATCACTACGGATACATCCAGTAGATGATCAGCTACTCAACATAGCCATAAACAA<br>TTGCATCCAGACACAGCCATTACAACCGGTCTTAAATGAATACATTATGATAGCGGCTGATGGA<br>TATGTTGCATCCCCTAAAAAACGTTATGATAGGAACACATTGACTATATCAAATGTTTTTTCAC<br>CAGTTTTCAATAGACTGTGTGTGTTATCAGGCACAACATACAGAGCTAGAACTTTACAATCTAT<br>GACTTTTATGTCGAGACTGTGGAAAACCAATGTTTTCAAAACATCACTAGAGGATGATATAGCT<br>AAAATGTATGCTGGAGCCGAAATTTCAATGACTACGATCGATGGAACAACAGCCAGTCTTGCTA<br>CAATAAACATTTCTGCGGCCGAACAAGCTTTAATAGGTATACTAAATGCGTCATTTTTTAGATT<br>CGACTTTAACTTAACTGGACCTCAAAACTCGTTAGGAGCTGCGATATCGGCGTTAATAGCGTTG<br>ATAGTACTACCAATTGACCAAGATACAATGGATAATGATACATATGATATGTTGTGTAACACTG<br>TTTTTAATGAAATGATGGCAAATGCAATGAACCTACCAGCGTTTGTGCGAAGAGCTGGAGATGA<br>AAATGCGTTTAGAAGATATGTGAATGGACCAATTCCGAGAGAAGCTTCTGCTTTTATTAGATTC<br>ATTTTGTTACGAAGAGGATGGTTGCTTTTTCAGAGATCTGATGATAGAACGATTCATTGTGACA<br>TCTTAGTACCAAATTGTGACACGCAGAATGTAAATGATCAACCATATGTTTTACTCTCAGACTT<br>CTTCGCTGCGATATTGGAATCTTCCAGACGTAATCCAAATCCAGGAAAAAATACATCCGCTAAT<br>TCGTTTAGACGACTAATTCGAGGACTTAGGGACATTGTCTGTAACAAAATCATGCCAGCTATGA<br>GGTTAATTAGGTACAATGTAGAAAGAATAGCTAGAATTCAAAGTATGTTACCATATTCTGCTGA<br>TCTTGCATTAGTTAACCCCTCTTTGAGAGATGAAAGATTGAGAGCAAACATCCCATTATCAGGA<br>TTTTTATCATTGTTAATGGGAATATCAAAGGCACCAGATGCATTTGATTGGACAACAGTATTAA

	CATTTTGTGATTCAATGCGAAAATTAAACTATGCTGAAACAATATCGGTCGAAGAAGCTCTAAC<br>CGTTGCTATTTCATCACATGATATAGATAAATCAGTATCAAAAAAAGACATTATAAAAGACGTA<br>TTGCACCCACCAACTTCAGCCGTAGCTGCGATTACAAAGGTTCCATCAGCGTCACTTTCTGCAT<br>TGCTATCAGATACGGTTTTAATTAACCTGGTAAAAAACTCAAAACCATTTAGGAAAATTACAGA<br>CATAGTGAAAGTTTTGAAAGCAGCTTTTCAACATTCACCGACTGCGAGTTATGGAGTAGTCAAA<br>GGCGCTCTATTGATGTCATCACCCCAAAATTTTAGGAGATCATCTCAATATGTTAAAAGAGATA<br>ATGTAATACACAATGTCGTAGATGGCATTCAAAAATTCAAAGCAGAAGATCTAGTTAAAGGCAA<br>TCACTTTCCTGGTCTAATTAATTCAATAAAGAACGGTGATGATATATATATAGAAGGACCTTTA<br>CCAGTAAGAACATCGCATTCATCGGAGGTCGCTACCGCTTCTTTCGCCTTTCTAACAATGAATT<br>CACCATATGACGCCTTCATTGATCCAACTGATTTACAACATCAAAGATTAATAAAAATTAGAGC<br>AGTTGACCATTTTTCAGACTCGTCAATTGACATGATACCATCGAAATTTGACAATTTGCTAGCT<br>AAGACATCAGTATTCGTACATGATGCTCAAAGCTTAATGGTACAGTCAAAATCAACAGTGAGAC<br>GGTTCAACTACCACGAATCACTATTACATATTGACATAGCAAATCTGATTAACTTCTCAGTTGC<br>TTTGCCAACTGAACTACAGCTATTCGATGGAACATTAGTGTATGATGTGTAG]GCGGCCGCGAT<br>C-3‘
Chimeric Flag-VP2/G-B	5’-GATCACGCGTGTCGACC**ATG**[GATTACAAGGATGACGACGATAAG][GATCCCGAAGGTTT<br>AATTTCAGATGCGATTAAAGCAATCGATGAAGCAAAGAATGATAAAAAGAAGATAATAGTAATA<br>GCTAAGCAGTTAGTCAACGATATTAAAGATACAGTTGAAGACTTGGATATTGATTTACTAAAAA<br>AATTGAAAGTACAATTTAGCGCAAAACGAATTGCAACAAAAGAAATTGATGTACTGATAAAAAA<br>TATTGAAGATAAAAAGAAAGAAAGTAAGAATGTGAGCGATAAAAAGGAGGAAAGCGGTAAAAAC<br>GATGATAATCAAAATCAGGAAAAAGGAAAGGATAATGAATCTGGAAAGAAAGAGAAAATCAATA<br>ACAGTAAAGGGGACGAAGGCGGACTAAGAGATAATGTACTTGGTAATTCAGATCCAGATAGTAA<br>ATTAACAAAAGAT][GTCTTCCAAATTAGAACAATTTTATCGAAAACGCTATTCGTTGATGTCG<br>AAAATGAAGATTATTCAGTATATATCCCTAATGAAACGACGAAATTGACTCCTGTGTCAATTGA<br>TGCAAGACCAATCCAGACTTACCATCCTAAAGCTTTGATGTACAAAGACACTGCGATTCTTCCC<br>TCTCATAGAGATGAGATATCAGATCAATATGGTACTGATGAGATATTATTTGATTCTCAC]ATG<br>TGATC-3’
VP2 OSU	5’-GATCCCCGGGTTAATACGACTCACTATA*GGCTATTAAAGGCTCA*[**ATG**GCGTACAGGAAGC<br>GCGGAGCTAAACGTGAAAACTCATCACAACAGAATGAACGTCTGCAAGAAAAAGAAGTTGAAAA<br>GAACATAGACGCATCTATGGAGAATAAAGCTAATAATAAAAAGCAACAATTATCTGATAAAGTG<br>TTATCACAAAAAGAAGAGATAATAACTGACGCACAAGATGATGTTAAGATAACTGATGAGGTTA<br>AGAAATCATCAAAAGAAGAATCGAAACAGCTTTTGGAGATATTGAAAACTAAAGAAGATCATCA<br>GAAAGAGATACAATATGAAATTTTACAGAAAACGATACCAACTTTTGAACCAAAAGAATCAATT<br>TTGAAAAAATTAGAAGACATAAAACCAGAACAGGCAAAAAAACAAACTAAATTGTTTAGAATAT<br>TTGAACCAAGACAATTACCAATTTATCGGGCAAATGGTGAGAGAGAATTGAGAAATAGATGGTA<br>TTGGAAATTGAAAAGAGACACGCTGCCAGATGGAGATTATGATGTCCGAGAATACTTCTTAAAT<br>TTATATGATCAGATATTGATAGAAATGCCAGATTATTTATTATTGAAAGATATGGCAGTGGAGA<br>ATAAAAATTCTAGGGATGCTGGTAAAGTCGTGGATTCCGAGACAGCAAGTATTTGTGATGCCAT<br>ATTTCAAGATGAGGAAACAGAGGGAGTCATTAGAAGGTTCATTGCAGATATGAGGCAACAAGTT<br>CAAGCTGACAGAAATGTTGTTAACTATCCATCAATTTTACATCCAATTGATCATGCATTCAATG<br>AATACTTTTTAAATCACCAATTAGTTGAACCATTAAATAATGAAATAATTTTTAATTATATACC<br>AGAAAGGATAAGAAACGATGTTAATTACATTCTAAACATGGATATGAATCTACCATCAACAGCT<br>AGATATATTAGACCAAATTTGCTGCAAGATAGACTAAATTTGCATGATAATTTTGAATCATTAT<br>GGGATACGATAACAACATCAAACTATATATTAGCTAGATCAGTTGTGCCTGATCTGAAGGAAAA<br>GGAGTTAGTGTCAACCGAAGCTCAAATACAGAAAATGTCCCAAGATTTGCAACTTGAAGCACTA

	ACTATACAATCTGAAACGCAGTTTCTGGCTGGTATAAATTCACAAGCAGCAAATGATTGTTTTA<br>AAACGCTAATAGCAGCCATGTTAAGCCAACGTACAATGTCATTAGAATTTGTGACTACAAATTA<br>CATGTCACTTATATCTGGTATGTGGTTACTAACAGTTATACCAAACGACATGTTTCTTCGTGAA<br>TCGCTAGTTGCATGCGAGTTGGCTATAATAAACACTATTGTTTATCCAGCGTTCGGAATGCAAA<br>GGATGCATTATAGAAATGGTGATCCACAGACTCCATTCCAAATAGCAGAACAGCAGATACAAAA<br>TTTTCAAGTAGCTAATTGGCTACACTTTATCAACAATAATAGATTTAGACAAGTTGTGATCGAC<br>GGAGTGTTGAATCAAACACTTAATGATAATATTAGAAATGGACAAGTTATTAATCAGTTGATGG<br>AAGCGTTAATGCAACTATCTAGACAACAGTTTCCAACTATGCCAGTTGATTATAAAAGATCAAT<br>ACAAAGAGGAATATTACTATTGTCAAACAGATTAGGTCAATTAGTTGACTTAACAAGACTATTA<br>TCATATAACTATGAAACACTGATGGCTTGCATAACCATGAATATGCAACATGTACAAACTCTTA<br>CTACTGAAAAATTACAATTAACTTCTGTTACATCTTTGTGTATGTTAATTGGAAATACTACAGT<br>TATTCCAAGTCCACAAACATTGTTTCACTATTACAATGTAAATGTAAATTTTCATTCAAACTAT<br>AACGAACGGATTAACGATGCAGTAGCAATTATTACAGCAGCCAATAGACTGAATTTATATCAGA<br>AGAAGATGAAGTCAATAGTTGAAGAATTTTTGAAGAGGCTGCAAATTTTCGATGTGCCACGAGT<br>GCCCGATGATCAAATGTATAGACTGAGAGATAGACTTAGATTGTTGCCGGTTGAAAGACGGAGA<br>CTTGATATATTCAATTTAATATTAATGAATATGGAGCAGATCGAGCGAGCTTCAGATAAAATTG<br>CACAAGGTGTAATAATTGCTTATAGAGATATGCAACTAGAGAGAGATGAAATGTATGGATACGT<br>CAATATTGCTAGAAATCTGGATGGATACCAACAAATTAATTTAGAAGAATTAATGAGGACAGGA<br>GATTATGGTCAAATTACTAACATGCTATTGAACAACCAGCCAGTGGCTCTAGTAGGAGCATTGC<br>CATTCGTAACAGACTCATCAGTTATATCACTCATTGCTAAGCTGGATGCTACAGTTTTCGCCCA<br>GATAGTCAAACTTAGGAAAGTAGATACCTTAAAACCGATATTGTATAAAATAAACTCTGATTCT<br>AATGATTTCTATTTAGTTGCAAATTACGATTGGATACCAACTTCCACTACAAAAGTGTACAAAC<br>AGGTACCGCAACCCTTTGACTTTAGAGCGTCAATGCATATGTTAACATCTAACCTCACTTTTAC<br>TGTTTATTCCGATTTGTTAGCTTTTGTTTCTGCAGATACAGTTGAACCGATCAACGCAGTTGCT<br>TTTGACAATATGCGCATTATGAACGAACTG**TAA**]*ACGCCAACCCCACTGTGGAGATATGACCGG*<br>*GTCGGCATGGCATCTCCAC*CTCCTCGATC-3'

* The restriction sites are underlined.

** Initiation and stop codons are labeled in bold.

*** Delimitation of tags and ORF regions are in between brackets.

**** 5'- and 3'-UTRs are label in italics.

Table S3: Oligonucleotides used in this study

Amplified DNA segment	Oligonucleotide sequence
V5-NSP5/D	fwd:5'-gatcacgcgtgccacc**atg**ggtaagcctatccctaaccc<br>tctcctcggtctcgattctacgatgatggatgatttagacttt-3'<br>rev:5'-gatcgcggccgc**tta**ataactttcagc-3'

V5-NSP5(15-195)/D	fwd:5′-gatcacgcgtgccacc**atg**ggtaagcctatccctaaccc tctcctcggtctcgattctacggaaatatctttaatttcgtca-3′<br>rev:5′-gatcgcggccgc**tta**ataactttcagc-3′
V5-NSP5/F	fwd:5′-gatcacgcgtgccacc**atg**ggtaagcctatccctaaccc tctcctcggtctcgattctacgatgagcatggatcttgatata-3′<br>rev:5′-gatcgcggccgc**tta**tagatcggatat-3′
V5-NSP5(19-218)/F	fwd:5′-gatcacgcgtgccacc**atg**ggtaagcctatccctaaccc tctcctcggtctcgattctacgataataggaggatctaatact-3′<br>rev:5′-gatcgcggccgc**tta**tagatcggatat-3′
Flag-VP2/A	fwd:5′-gatcacgcgtgtcgacc**atg**gattacaaggatgacgacga taaggcgtatcgaaaacgtggagcg-3′<br>rev:5′-gatcgcggccgc**tta**cagttcgttcatgatgcgcat-3′
Flag-VP2/B	fwd:5′-gatcacgcgtgtcgacc**atg**gattacaaggatgacgacga taaggattcaactgccttagtcgaa-3′<br>rev:5′-gatcgcggccgc**tta**atcttcataaaccaacat-3′
Flag-VP2/C	fwd:5′-gatcacgcgtgtcgacc**atg**gattacaaggatgacgacga taagataagcagaaacaggcgcaga-3′<br>rev:5′-gatcgcggccgc**tta**tgtttcttgcataattct-3′
Flag-VP2/F	fwd:5′-gatcacgcgtgtcgacc**atg**gattacaaggatgacgacga taagtccagtaataaggcaaaacaa-3′<br>rev:5′-gatcgcggccgc**tta**gagcgttctgacgattct-3′
Flag-VP2/I	fwd:5′-gatcacgcgtgtcgacc**atg**gattacaaggatgacgacga taagtcactaatagggacgctcgag-3′<br>rev: gatcgcggccgc**cta**cacatcatacactaatgt
NanoLuc-Flag-VP2/A	fwd:5′-gatcacgcgtgattacaaggatgacgacgat-3′<br>rev:5′-gatcgcggccgc**tta**cagttcgttcatgatgcgcat-3′
NanoLuc-Flag-VP2/B	fwd:5′-gatcacgcgtgattacaaggatgacgacgat-3′<br>rev:5′-gatcgcggccgc**tta**atcttcataaaccaacat-3′
NanoLuc-Flag-VP2/C	fwd:5′-gatcacgcgtgattacaaggatgacgacgat-3′<br>rev:5′-gatcgcggccgc**tta**tgtttcttgcataattct-3′

NanoLuc-Flag-VP2/D	fwd:5′-gactacgcgtgattacaaggatgacgacgataag-3′<br>rev:5′-gatcgcggccgc**cta**cacacttaagataatcct-3′
NanoLuc-Flag-VP2/F	fwd:5′-gatcacgcgtgattacaaggatgacgacgat-3′<br>rev:5′-gatcgcggccgc**tta**gagcgttctgacgattct-3′
NanoLuc-Flag-VP2/G	fwd:5′-gatcacgcgtgattacaaggatgacgacgat-3′<br>rev:5′-gatcgcggccgc**tca**cgaaggtttagcttcata-3′
NanoLuc-Flag-VP2/H	fwd:5′-gatcacgcgtgattacaaggatgacgacgat-3′<br>rev:5′-gatcgcggccgc**cta**ctgttcaaacactagtaa-3′
NanoLuc-Flag-VP2/I	fwd:5′-gatcacgcgtgattacaaggatgacgacgat-3′<br>rev:5′-gatcgcggccgc**cta**cacatcatacactaatgt-3′
NanoLuc-Flag-VP2/J	fwd:5′-gatcacgcgtgattacaaggatgacgacgat-3′<br>rev:5′-gatcgcggccgcttactgctcaaaaaccaaaag-3′

* Restriction sites are underlined.

** Initiation and stop codons are labeled in bold.

Supplemental Materials and Methods

Plasmid constructs. pcDNA-VP2 SA11 was described previously (2). pT7-VP2 (OSU) was obtained by ligation of a synthetic DNA segment (Genscript) containing genome segment 2 of rotavirus porcine strain OSU (GenBank: MT0066201.1) flanked downstream by a hepatitis delta virus ribozyme (Table S2) in *Xma*I and *BseR*I restriction sites in pT7-VP4(SA11)(3).

Co-immunoprecipitation and immunoblotting. 1.2×10^6 MA/cytBirA cells were infected with vvT7.3 at a multiplicity of infection (MOI) of 3 PFU/cell. Then, the cells were transfected with Lipofectamine 2000 (Thermo Fisher Scientific) in a ratio 1:2 of NSP5-BAP and Flag-VP2 following the manufacturer's instructions. After adding the transfection mixture, the cells were immediately supplemented with 100 µM biotin. At 16 hpt, the cells were lysed in 180 µL of TNN buffer [100 mM Tris-HCl, pH 8.0, 250 mM NaCl, 0.5% Nonidet P-40, and cOmplete protease inhibitor cocktail (Roche, Switzerland)] for 10 min on ice. For NSP5-BAP and Flag-

VP2 assays, the cells were crosslinked with 300 µM DSP prior to lysis as described in detail by Eichwald et al., 2004(4). The cell lysates were clarified by centrifugation at 17,000 × *g* for 7 min and 4°C and then transferred to a new 1.5 mL tube. The input corresponded to 15 µL of cell lysate. For immunoprecipitation, the cell lysate was split into equal volumes and combined with 2 µg mouse mAb anti-Flag (clone M2, Merck) or 2 µg mouse IgG2a kappa isotype control (clone eBM2a; eBioscience, 14–4724-82), and incubated at 4°C for 30 min with rotation. The cell lysates were combined with 50 µL of Protein G Dynabeads (ThermoFisher, 10004D), equilibrated in TNN, and re-incubated at 4°C for 30 min with rotation. The bead-bound antibody-antigen complexes were washed four times with 500 µL TNN, eluted with SDS sample buffer, and resolved by SDS-PAGE. The proteins were detected by immunoblotting, as described by Lee et al, 2024 (5).

Proteinase K assay. MA104 cells at a density of 2x10^5 cells per well in a 12-multiwell plate were infected with vvT7.3(MOI 1 PFU/cell). At 1 h post-infection, the cells were transfected with 2 µg of DNA plasmid encoding Flag-VP2 using 3 µl of Lipofectamine 2000 (ThermoFisher Scientific) according to the manufacturer's instructions. At 16 hpt, the cells were lysed in 30 µl of Laemmli sample buffer (8% sodium dodecyl sulfate, 200 mM Tris pH 6.8, 40% glycerol, 572 mM 2-mercaptoethanol) and divided in two identical volumes. Then, the samples were untreated or treated with 0.25 µg (12.5 mU) of proteinase K diluted in TNN buffer (100 mM Tris-HCl, pH 8.0, 250 mM NaCl, 0.5% Nonidet P-40) for 2 min in ice. The reaction was stopped by the addition of 5 µl of cOmplete protease inhibitor cocktail (Roche, Switzerland) (1 tablet in 1 ml TNN buffer). The samples were heated for 5 min at 95ºC and sonicated for 5 sec at 14 Hz. The samples were resolved in 12% SDS-PAGE and digested proteins were detected by immunoblotting (5) using mAb anti-Flag.

Pairwise comparison of RV strains within their RV species. The accession numbers shown in the tree were collected, and the nucleic acid sequence files were downloaded from GenBank.

The sequences were labelled according to their branches on the tree. The pairwise alignments were performed against each sequence. From the 59 sequences of NSP5, 3481 pairwise alignments were performed. From the 133 sequences of VP2, 17556 pairwise alignments were performed. Thereof, the unique alignments were selected and the self was deleted.

Supplemental References

1. McClain B, Settembre E, Temple BR, Bellamy AR, Harrison SC. 2010. X-ray crystal structure of the rotavirus inner capsid particle at 3.8 A resolution. J Mol Biol 397:587-99.
2. Arnoldi F, Campagna M, Eichwald C, Desselberger U, Burrone OR. 2007. Interaction of rotavirus polymerase VP1 with nonstructural protein NSP5 is stronger than that with NSP2. J Virol 81:2128-37.
3. Kanai Y, Komoto S, Kawagishi T, Nouda R, Nagasawa N, Onishi M, Matsuura Y, Taniguchi K, Kobayashi T. 2017. Entirely plasmid-based reverse genetics system for rotaviruses. Proc Natl Acad Sci U S A 114:2349-2354.
4. Eichwald C, Rodriguez JF, Burrone OR. 2004. Characterization of rotavirus NSP2/NSP5 interactions and the dynamics of viroplasm formation. Journal of General Virology 85:625-634.
5. Lee M, Cosic A, Tobler K, Aguilar C, Fraefel C, Eichwald C. 2024. Characterization of viroplasm-like structures by co-expression of NSP5 and NSP2 across rotavirus species A to J. J Virol 98:e0097524.
